# Argymekosides A‐C, Three Undescribed 21*α*‐Hydroxyoleanolic Acid Glycosides From *Argyreia mekongensis* and Their Cytotoxic Activity

**DOI:** 10.1002/cbdv.71814

**Published:** 2026-10-12

**Authors:** Tran Thi Ngoc Hanh, Nguyen Huu Toan Phan, Nguyen Thi Thu Hien, Pham Van Huyen, Nguyen Huu Huong Duyen, Nguyen Thi Dieu Thuan, Duong Thi Hai Yen, Bui Huu Tai, Phan Van Kiem

**Affiliations:** ^1^ Institute of Life Sciences Vietnam Academy of Science and Technology (VAST) Ho Chi Minh City Vietnam; ^2^ Faculty of Chemistry Graduate University of Science and Technology, VAST Hanoi Vietnam; ^3^ Institute of Chemistry VAST Hanoi Vietnam

**Keywords:** Argymekoside, *Argyreia mekongensis*, Convolvulaceae, cytotoxic activity, oleanolic acid glycoside

## Abstract

Three previously undescribed 21*α*‐hydroxyoleanolic acid glycosides, designated Argymekosides A–C (**1–3**), together with eight known compounds (**4–11**), were isolated from the aerial parts of *Argyreia mekongensis* Gagnep. & Courchet. Their structures were elucidated by comprehensive spectroscopic analyses, including HRESIMS and 1D and 2D nuclear magnetic resonance (NMR) spectroscopy, as well as comparison with published data. The absolute configurations of three new compounds were determined by electronic circular dichroism (ECD) calculations. The isolated compounds were evaluated for their cytotoxic activities against HepG2, MCF‐7, and SK‐LU‐1 human cancer cell lines. Compound **5** exhibited potent cytotoxicity toward all three cell lines, with IC_50_ values of 5.06, 5.70, and 5.79 µM, respectively. Compound **4** displayed moderate cytotoxic activity against all tested cell lines (IC_50_ = 26.73–35.53 µM), whereas compound **6** showed weak activity against HepG2 (IC_50_ = 72.03 µM) and MCF‐7 cells (IC_50_ = 91.51 µM). The remaining compounds were inactive, with IC_50_ values exceeding 100 µM.

## Graphical abstract

Isolation and determination of three new and eight known compounds from the aerial parts of *Argyreia mekongensis* with their cytotoxic activity against HepG2, MCF‐7, and SK‐LU‐1 cell lines.

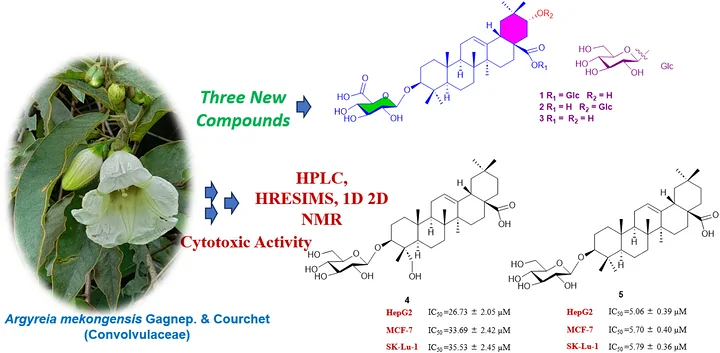

## 1 Introduction

The genus *Argyreia* (family Convolvulaceae) comprises approximately 130–150 species distributed throughout tropical and subtropical Asia, with centers of diversity in India, Southeast Asia, and southern China [1, 2]. Several species have long been used in traditional medicine for the treatment of neurological disorders, inflammatory diseases, diabetes, gastrointestinal ailments, skin diseases, and sexual dysfunction. Phytochemical investigations have revealed that *Argyreia* is a valuable source of chemically diverse natural products, particularly ergoline alkaloids, flavonoids, phenolic compounds, triterpenoids, phytosterols, and coumarins [3, 4, 5, 6, 7, 8, 9, 10, 11, 12]. Vietnam records approximately 20–25 species of *Argyreia*, with many distributed across mountainous regions in Cambodia, Laos, and Vietnam [13]. Plant *Argyreia mekongensis* remains an underexplored species from both phytochemical and pharmacological perspectives. At present, no peer‐reviewed publications have reported its chemical constituents or biological activities. During the screening of biological activity in plants from the Lam Dong region (Project TN3/T14 under the Tay Nguyen 3 Program), we discovered that this plant exhibited significant activity against Hep‐G2 and RD cancer cell lines and, therefore, was selected for further study. The present paper reports the isolation and structure elucidation of 11 compounds, including three undescribed 21*α*‐hydroxyoleanolic acid glycosides, designated argymekosides A–C (**1–3**), and their cytotoxicity against three human cancer cell lines, hepatocellular carcinoma (HepG2), breast adenocarcinoma (MCF‐7), and lung adenocarcinoma cell line (SK‐LU‐1).

## 2 Results and Discussion

Comprehensive chromatographic separation of the methanolic extract of the aerial parts of *A. mekongensis* led to the isolation of 11 compounds (**1–11**), including three previously undescribed compounds (**1–3**).

Compound **1** (Figure 1) was obtained as a white solid, which showed a pseudo‐molecular ion peak at *m/z* 809.4327 [M − H]^−^ on its HRESIMS (calculated for [C_42_H_65_O_15_]^−^: 809.4329), determining the molecular formula of C_42_H_66_O_15_ and 10° of unsaturation. The ^1^H NMR spectrum of **1** exhibited seven methyl singlets [*δ*_H_ 1.03 (H_3_‐23), 0.84 (H_3_‐24), 0.95 (H_3_‐25), 0.79 (H_3_‐26), 1.17 (H_3_‐27), 0.93 (H_3_‐29), 0.95 (H_3_‐30), each 3H], one olefinic proton at *δ*_H_ 5.27 (1H, t, *J* = 3.6 Hz, H‐12), and two anomeric protons at *δ*_H_ 4.33 (1H, d, *J* = 7.8 Hz, H‐1′) and *δ*_H_ 5.38 (d, *J* = 8.4 Hz, H‐1′′). In addition, two methine carbinol protons were identified at *δ*_H_ 3.20 (1H, dd, *J* = 11.4, 3.0 Hz, H‐3) and 3.42 (H‐21, overlapped). These suggested that **1** was a Δ^12^‐oleanane triterpene bearing two sugar moieties [14]. Combined analysis of the ^13^C NMR and HSQC of **1** has determined 42 carbon atoms, which were divided into 7 methyls, 10 methylenes, 16 methines, and 9 quaternary carbons. Of these, 30C were assigned to an oleanane triterpene aglycone and 12C were assigned to two hexose sugar moieties. In the aglycone part, a carbonyl group (*δ*_C_ 177.8), a Δ^12^ double bond [*δ*_C_ 123,9 (C‐12) and 144.8 (C‐13)], and two methine carbinol groups [*δ*_C_ 90.7 (C‐3) and 74.9 (C‐21)] were identified. The hydroxy group at C‐21 was indicated by HMBC correlations from H_3_‐29 (*δ*_H_ 0.93)/H_3_‐30 (*δ*_H_ 0.95) to C‐19 (*δ*_C_ 42.2)/C‐20 (*δ*_C_ 36.1)/C‐21 (*δ*_C_ 74.9), and from COSY correlations of H‐18/H‐19 and H‐21/H‐22. In the sugar moieties, two anomeric carbons at *δ*_C_ 106.7 (C‐1′) and 95.8 (C‐1′′), one glucose oxygenated methylene carbon at *δ*_C_ 62.8 (C‐6′′), and one carbonyl carbon at *δ*_C_ 177.0 (C‐6′). Proton H‐5′ appeared as a doublet at *δ*_H_ 3.54 (*J* = 9.6 Hz), which had an HSQC correlation with C‐5′ (*δ*_C_ 76.5) and showed an HMBC correlation with C‐6′ suggested the presence of a glucuronic acid moiety [14]. This sugar was linked to C‐3, as evident by HMBC correlation from H‐1′ (*δ*_H_ 4.33) to C‐3 (*δ*_C_ 90.7). The higher‐field signal of the anomeric carbon at *δ*_C_ 95.8, together with HMBC correlation from H‐1′′ (*δ*_H_ 5.38) to C‐28 (*δ*_C_ 177.8), confirmed the glucose unit linked to C‐28 by an ester linkage (Figure 2). The nuclear magnetic resonance (NMR) data of **1** were similar to those of chikusetsusaponin IVa [14] except the C‐21 methylene signals were replaced by a methine carbinol signal. It is precisely this substitution that altered the chemical shift of the ring F of **1** relative to chikusetsusaponin IVa [14] (Table 1). The large ^3^ *J*_2,3_ value (11.4 Hz) of proton H‐3 (*δ*_H_ 3.20), together with the NOESY correlation between H‐3 and H‐5 (*δ*_H_ 0.78), suggested an *α/axial* orientation for H‐3 (Figure 3). The carbon chemical shift of C‐19 (*δ*_C_ 42.2) of **1** was similar to that of 3*β*‐[(O‐*β*‐D‐glucopyranosyl‐(1→4)‐*O*‐*β*‐D‐ribopyranosyl‐(1→3)‐*O*‐*α*‐L‐rhamnopyranosyl‐(1→2)‐*α*‐L‐arabinopyranosyl)oxy]‐21*α*‐hydroxyolean‐12‐en‐28‐oic acid *O*‐*α*‐L‐rhamnopyranosyl‐(1→4)‐*O*‐*β*‐D‐glucopyranosyl‐(1→6)‐*β*‐D‐glucopyranosyl ester (21*α*‐OH, *δ*_C‐19 =_ 41.3) [15], clematangoside B (21*α*‐OH, *δ*_C‐19 =_ 41.3) [16], and difference from that of *β*‐D‐glucopyranosyl 3*β*‐[*O*‐*α*‐L‐rhamnopyranosyl‐(1→3)‐*O*‐*β*‐D‐glucopyranuronosyloxy]machaerinate (21*β*‐OH, *δ*_C‐19 =_ 48.3) [17], and some 21*β*‐hydroxyoleanolic acid derivatives (*δ*_C‐19_ 46.3∼47.1) [18, 19], suggested *α/axial*‐orientation for the hydroxy group at C‐21. This was further confirmed by NOESY correlations observed between H‐21_β/equatorial_ (*δ*_H_ 3.42) and both H_3_‐29 (*δ*_H_ 0.93) and H_3_‐30 (*δ*_H_ 0.95), as well as by the small ^3^ *J*_21,22_ value (3.6 Hz) of two protons at *δ*_H_ 1.79 and 1.91 (H_2_‐22) (Table 1). The large ^3^ *J*_1,2_ values of the anomeric protons at *δ*_H_ 4.33 (7.8 Hz) and 5.38 (8.4 Hz) suggested the *β*‐form of the glycosidic linkages. Acid hydrolysis of **1** yielded monosaccharides (D‐glucose and D‐glucuronic acid) identified by comparison with standard monosaccharides using TLC and confirmed by the positive signs of optical rotations [20, 21] (Supporting Information). The absolute configuration of **1** was determined as (3*S*,5*R*,8*R*,9*R*,10*R*,13*S*,17*R*,18*S*,21*R*) by comparing its experimental and calculated ECD spectra (Figure 4). Consequently, compound **1** was determined as (21*R*)‐hydroxyoleanolic acid 3‐*O*‐*β*‐D‐glucuronopyranoside‐28‐*O*‐*β*‐D‐glucopyranoside, a new compound named argymekoside A.

**FIGURE 1:**
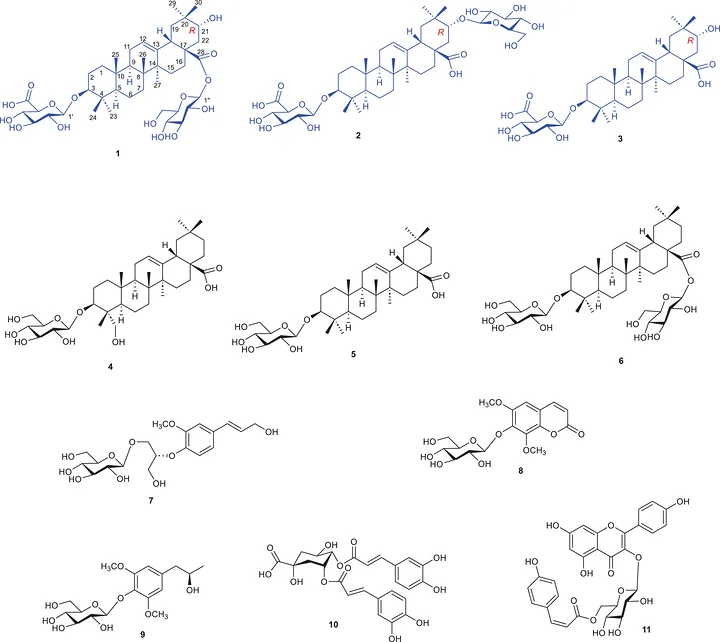
Chemical structures of compounds **1–11**.

**FIGURE 2:**
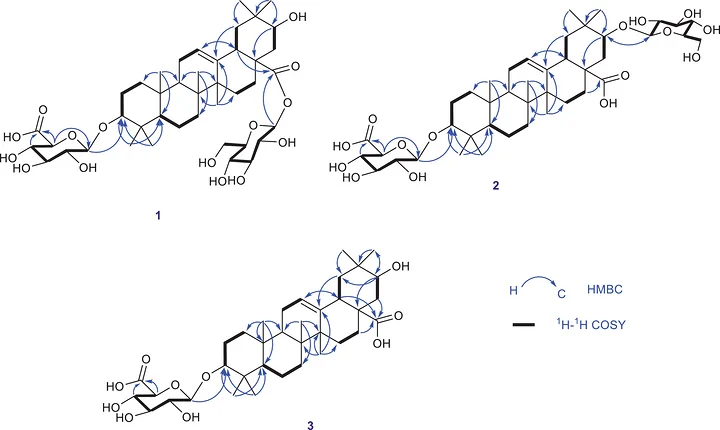
Key HMBC and COSY correlations of compounds **1–3**.

**TABLE 1:** NMR spectral data of compounds **1**–**3**.

No.	1 (CD_3_OD)		2 (CD_3_OD)		3 (DMSO‐d_6_)	
	*δ*_C_ ^a^	*δ*_H_ ^b^ (*J* in Hz)	*δ*_C_ ^a^	*δ*_H_ ^b^ (*J* in Hz)	*δ*_C_ ^a^	*δ*_H_ ^b^ (*J* in Hz)
1	39.9	1.00 (m)/1.60 (m)	39.9	1.00 (m)/1.60 (m)	38.2	0.88 (m)/1.50 (m)
2	26.9	1.69 (m)/1.99 (m)	26.9	1.68 (m)/2.00 (m)	25.3	1.50 (m)/1.91 (m)
3	90.7	3.20 (dd, 11.4, 3.0)	90.7	3.19 (dd, 11.4, 3.0)	87.9	3.02 (dd, 11.4, 3.6)
4	40.0	—	40.2	—	38.7	—
5	57.1	0.78 (br d, 10.2)	57.1	0.78 (br d, 11.4)	55.0	0.71^c^
6	19.3	1.38 (td, 10.2, 3.0) 1.53 (br d, 10.2)	19.4	1.40 (td, 10.2, 3.0) 1.53 (br d, 10.2)	17.8	1.30 (m) 1.45 (m)
7	34.1	1.48 (dd, 10.2, 3.6)/1.30 (m)	34.3	1.47 (dd, 12.6, 3.0)/1.32 (m)	32.6	1.14 (m)/1.39 (m)
8	40.6	—	40.5	—	38.6	—
9	49.0	1.59^c^	49.1	1.59^c^	47.1	1.50^c^
10	37.9	—	37.9	—	36.3	—
11	24.6	1.86‐1.91 (m)	24.6	1.86‐1.90 (m)	22.9	1.78‐1.82 (m)
12	123.9	5.27 (t, 3.6)	123.1	5.25 (t, 3.0)	121.3	5.17 (br s)
13	144.8	—	145.9	—	143.9	—
14	43.2	—	43.2	—	41.6	—
15	29.2	1.04 (m)/1.65 (m)	29.3	1.00 (m)/1.66 (m)	27.6	0.92 (m)/1.50 (m)
16	27.4	2.45 (td, 13.8, 3.6) 1.86 (m)	27.7	2.33 (td, 13.8, 2.4) 1.75 (m)	26.0	2.34 (dd, 12.6, 11.4) 1.76 (m)
17	48.1	—	48.3	—	45.6	—
18	42.4	2.92 (dd, 14.4, 3.0)	42.6	2.98 (dd, 14.4, 2.4)	40.7	2.81 (dd, 13.8, 3.0)
19	42.2	2.13 (t, 14.4) 0.98 (dd, 14.4, 4.2)	43.5	2.09 (t, 13.8) 0.97 (dd, 13.8, 2.4)	40.9	2.00 (t, 13.8) 0.96 (dd, 13.8, 2.4)
20	36.1	—	35.8	—	34.8	—
21	74.9	3.42^c^	80.5	3.61 (t, 3.0)	72.1	3.28 (br s)
22	39.6	1.79 (dd, 15.0, 3.6) 1.91 (dd, 15.0, 3.6)	35.8	1.75 (dd, 15.0, 3.6) 1.91 (dd, 15.0, 3.6)	38.8	1.73 (dd, 14.4, 2.4) 1.59 (dd, 15.0, 3.6)
23	28.5	1.03 (s)	28.6	1.04 (s)	27.7	0.97 (s)
24	17.0	0.84 (s)	17.0	0.84 (s)	16.5	0.76 (s)
25	16.1	0.95 (s)	16.1	0.95 (s)	15.2	0.87 (s)
26	17.8	0.79 (s)	18.0	0.84 (s)	16.9	0.72 (s)
27	25.7	1.17 (s)	25.8	1.16 (s)	25.3	1.09 (s)
28	177.8	—	184.0^d^	—	178.8^d^	—
29	27.9	0.93 (s)	28.3	0.95 (s)	27.6	0.84 (s)
30	25.4	0.95 (s)	25.8	1.01 (s)	24.6	0.87 (s)
3‐O‐GluA			3‐O‐GluA		3‐O‐GluA	
1′	106.7	4.33 (d, 7.8)	106.7	4.33 (d, 7.8)	105.3	4.13 (d, 7.8)
2′	75.6	3.23 (dd, 9.0, 7.8)	75.6	3.23 (dd, 9.0, 7.8)	73.9	2.95 (dd, 9.0, 7.8)
3′	78.0	3.36 (t, 9.0)	78.1	3.36 (t, 9.0)	76.8	3.10 (t, 9.0)
4′	73.8	3.43^c^	73.8	3.43 (t, 9.6)	72.1	3.09 (t, 9.0)
5′	76.5	3.54 (d, 9.6)	76.6	3.53 (d, 9.6)	73.9	3.22 (d, 9.0)
6′	177.0	—	177.0	—	173.0	—
28‐O‐Glc			21‐O‐Glc			
1′′	95.8	5.38 (d, 8.4)	101.4	4.37 (d, 7.8)	—	—
2′′	74.0	3.32 (dd, 9.0, 8.4)	75.5	3.18 (dd, 9.0, 7.8)	—	—
3′′	78.3	3.42^c^	78.5	3.37^c^	—	—
4′′	71.2	3.34 (t, 9.0)	72.1	3.28 (t, 9.0)	—	—
5′′	78.7	3.34 (m)	77.8	3.22 (m)	—	—
6′′	62.8	3.67 (dd, 11.4, 4.8) 3.81 (br d, 11.4)	63.0	3.66 (dd, 12.0, 5.4) 3.84 (dd, 12.0, 2.4)	—	—

^a^ 50 MHz.

^b^ 600 MHz.

^c^ Ovelapped signals.

^d^ Signal identified by HMBC spectrum, GluA: glucuronic acid, Glc: glucose.

**FIGURE 3:**
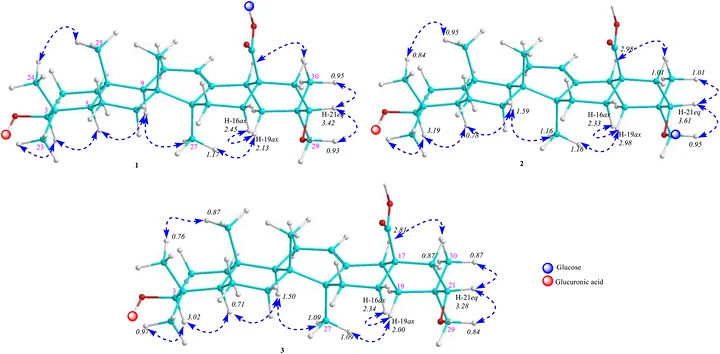
NOE correlations of compounds **1–3**.

**FIGURE 4:**
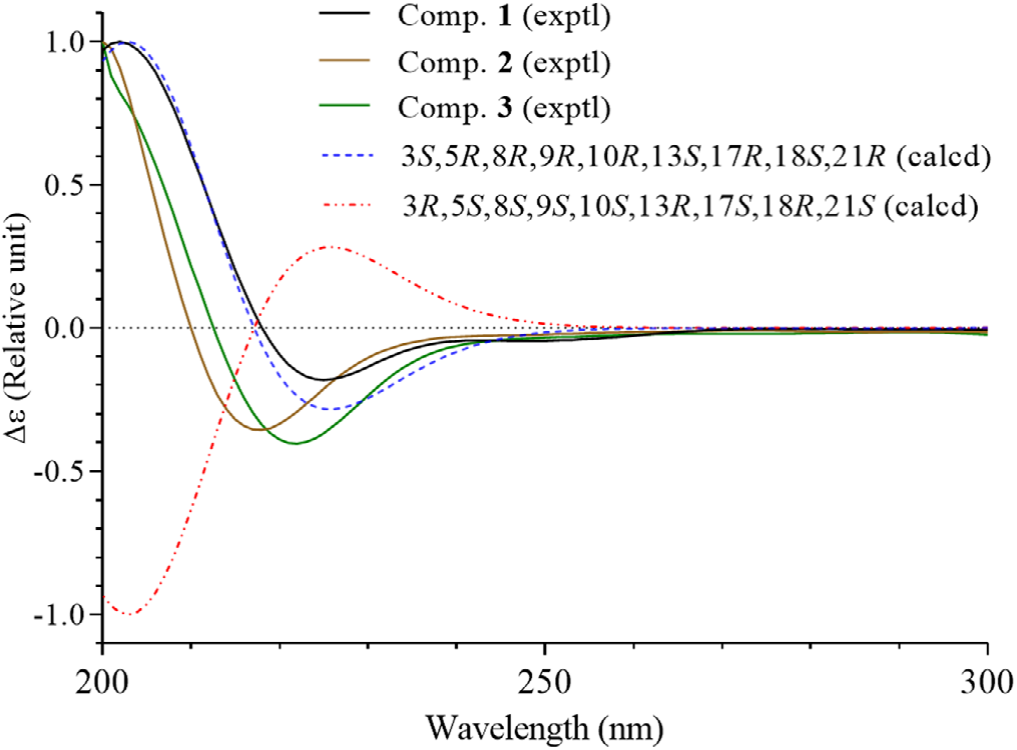
Experimental and calculated ECD spectra of compounds **1–3**.

Compound (**2**) was obtained as a white solid. The HRESIMS of **2** exhibited a pseudo‐molecular ion peak at *m/z* 811.4478 [M+H]^+^ (calculated for [C_42_H_67_O_15_]^+^: 811.44785), determining the molecular formula of C_42_H_66_O_15_ and 10° of unsaturation, similar to that of **1**. The ^1^H and ^13^C NMR spectra of **2** were very similar to the corresponding spectra of **1,** suggesting a Δ^12^‐oleanane triterpene glycoside (Table 1). The observable differences were the significant downfield shifts of C‐28 (from *δ*_C_ 177.8 to 184.0), C‐21 (from *δ*_C_ 74.9 to 80.5), and C‐1'' (from *δ*_C_ 95.8 to 101.4) [15]. These suggested that the glucose moiety was linked to C‐21 in **2** instead of C‐28 in **1**. This was further confirmed by HMBC correlation from H‐1ʹʹ (*δ*_H_ 4.37) to C‐21 (*δ*_C_ 80.5). The stereochemistry of **2** was shown to be identical to that of **1**, as evidenced by the NOESY spectrum analysis (Figure 3). Especially, H‐21 appeared at *δ*_H_ 3.61 as a triplet with a small proton–proton coupling constant (3.0 Hz), confirming a *β/equatorial* orientation for this proton. The glucuronic acid unit was linked to C‐3 as indicated by HMBC correlation from H‐1ʹ (*δ*_H_ 4.33) to C‐3 (*δ*_C_ 90.7). The large ^3^ *J*_1,2_ values (7.8 Hz) of the two anomeric protons suggested the *β*‐form of both glycosidic linkages. Acid hydrolysis of **2** yielded monosaccharides (D‐glucose and D‐glucuronic acid) identified by comparison with standard monosaccharides using TLC and confirmed by the positive signs of optical rotations [20, 21] (Supporting Information). The absolute configuration of **2** was determined as (3*S*,5*R*,8*R*,9*R*,10*R*,13*S*,17*R*,18*S*,21*R*) by comparing its experimental and calculated ECD spectra (Figure 4). From the above evidence, compound **2** was determined as (21*R*)‐hydroxyoleanolic acid 3‐*O*‐*β*‐D‐glucuronopyranoside‐21‐*O*‐*β*‐D‐glucopyranoside, a new compound named argymekoside B.

Compound (**3**) was obtained as a white solid. Its molecular formula was C_36_H_56_O_10_ as determined by HRESIMS (found *m/z* 649.3956 [M + H]^+^, calcd for [C_36_H_57_O_10_]^+^: 649.3946). The NMR data of **3** were similar to the corresponding data of **2**, except that the signals characteristic of the glucose moiety attached at C‐21 were no longer observed (Table 1). This structural change was accompanied by pronounced chemical‐shift changes, with the C‐21 signal moving upfield from *δ*_C_ 80.5 to 72.1, whereas the C‐22 signal shifted downfield from *δ*_C_ 35.8 to 38.8. The broad singlet signal at *δ*_H_ 3.28 (H‐21) correlated with carbon at *δ*_C_ 72.1 (C‐21) on the HSQC spectrum, as well as H_3_‐29/H_3_‐30 correlated to C‐21 on the HMBC spectrum, further confirming the 21*α*‐OH group. The NOESY cross peak between H‐3 (*δ*_H_ 3.02) and H‐5 (*δ*_H_ 0.71) indicated *anpha/axial* orientation for H‐3. The proton H‐5′ at *δ*_H_ 3.22 appeared as a doublet (*J* = 9.0 Hz), which showed HMBC correlations to C‐6′ (*δ*_C_ 173.0) and C‐1′ (*δ*_C_ 105.3), suggesting the presence of a glucuronic acid, which was linked to C‐3 as determined by HMBC correlation from H‐1ʹ (*δ*_H_ 4.13) to C‐3 (*δ*_C_ 87.9). This sugar was identified as a D‐glucuronic acid by comparison with standard monosaccharides using TLC and confirmed by the positive signs of optical rotation after acid hydrolysis [20, 21] (Supporting Information). The large proton coupling constant of the anomeric proton at 4.13 (7.8 Hz) revealed the *β*‐form of glycosidic linkage. The absolute configuration of **3** was determined as (3*S*,5*R*,8*R*,9*R*,10*R*,13*S*,17*R*,18*S*,21*R*) by comparing its experimental and calculated ECD spectra (Figure 4). Thus, compound **3** was elucidated as (21*R*)‐hydroxyoleanolic acid 3‐*O*‐*β*‐D‐glucuronopyranoside, a new compound named argymekoside C.

The known compounds were identified as HN‐saponin D1 (**4**) [22], androseptoside A (**5**) [23], lucyoside H (**6**) [24], 3‐Hydroxy‐2‐[4‐[(1E)‐3‐hydroxy‐1‐propen‐1‐yl]‐2‐methoxyphenoxy]propyl *β*‐D‐glucopyranoside (**7**) [25], isofraxidin 7‐O‐*β*‐D‐glucopyranoside (**8**) [26], pipercroside A (**9**) [27], 3,4‐di‐O‐caffeoylquinic acid (**10**) [28], *cis*‐tiliroside (**11**) [29], based on comprehensive analyses of their NMR and MS data and comparison with published literature values. The ^1^H and ^13^C NMR spectra of compounds **4**–**11** were shown in Figures S31–S38, and their ^1^H and ^13^C NMR data were shown in Tables S2–S9.

Interestingly, 21*α*‐hydroxy derivatives of oleanolic acid are exceptionally rare in nature and, to the best of our knowledge, have not previously been reported from the Convolvulaceae family. The occurrence of these uncommon derivatives in *Argyreia* therefore represents a potentially valuable chemotaxonomic marker for the genus.

Compounds **1–11** were evaluated for their cytotoxic effects against three human cancer cell lines, HepG2, MCF‐7, and SK‐LU‐1 (Table S1) [30, 31], with the results summarized in Table 2. Among the tested compounds, compound **5** emerged as the most potent, exhibiting strong growth‐inhibitory activity against all three cell lines, with IC_50_ values ranging from 5.06 to 5.79 µM. Compound **4** also displayed appreciable cytotoxicity, although considerably weaker than that of **5**, with IC_50_ values of 26.73–35.53 µM. In contrast, compound **6** showed only weak inhibitory activity against HepG2 and MCF‐7 cells, with IC_50_ values of 72.03–91.51 µM.

**TABLE 2:** Cytotoxic effects of compounds **1–11** on the cell lines.

Compounds	The cell lines (IC_50_ µM)		
	HepG2	MCF‐7	SK‐LU‐1
**1**	> 100	> 100	> 100
**2**	> 100	> 100	> 100
**3**	> 100	> 100	> 100
**4**	26.73 ± 2.05	33.69 ± 2.42	35.53 ± 2.45
**5**	5.06 ± 0.39	5.70 ± 0.40	5.79 ± 0.36
**6**	72.03 ± 4.69	91.51 ± 3.95	> 100
**7**	> 100	> 100	> 100
**8**	> 100	> 100	> 100
**9**	> 100	> 100	> 100
**10**	> 100	> 100	> 100
**11**	> 100	> 100	> 100
**Ellipticine**	0.32 ± 0.03	0.35 ± 0.02	0.39 ± 0.02

**Ellipticine** was used as a positive control compound.

A preliminary structure‐activity relationship analysis suggested that the glucuronic acid sugar moiety may reduce the cytotoxic activity. Among these triterpene derivatives, compound **5** exhibited the best possible activity, which may be partly attributable to its structural similarity to oleanolic acid, a triterpenoid widely reported to exhibit cytotoxic potential [32, 33, 34].

## 3 Conclusions

In conclusion, three new 21*α*‐hydroxyoleanolic acid glycosides, Argymekosides A–C (**1–3**), together with eight known compounds (**4–11**), were isolated from the aerial parts of *A. mekongensis*. Their structures were elucidated by comprehensive spectroscopic analyses and comparison with published data. Among the isolated compounds, compound **5** exhibited potent cytotoxicity against HepG2, MCF‐7, and SK‐LU‐1 cells, with IC_50_ values of 5.06, 5.70, and 5.79 µM, respectively. Compound **4** showed moderate activity against all three cell lines, whereas compound **6** exhibited weak activity against HepG2 and MCF‐7 cells. The remaining compounds were inactive at concentrations below 100 µM. These findings highlight the cytotoxic potential of the triterpenoid constituents of *A. mekongensis* and provide further insight into the phytochemical and biological significance of this species.

## 4 Experimental Section

### 4.1 General Experimental Procedures

The equipment used for this study is the same as in previous reports [21, 30] and described in the Supporting Information.

### 4.2 Plant Material

The Aerial parts of *A. mekongensis *Gagnep. & Courchet collected in Ngoan Muc Pass, Lam Son, Khanh Hoa during October 2025 and were identified by Dr. Nong Van Duy at the Institute of Life Sciences, VAST. The voucher specimen (No. TN3/633) was deposited at the Institute of Life Sciences, VAST.

### 4.3 Extraction and Isolation

The dried aerial parts of *A. mekongensis* (5.0 kg) were pulverized and exhaustively extracted with methanol under ultrasonic irradiation at room temperature (3 × 17 L, 2 h per extraction). Concentration of the combined extracts under reduced pressure afforded 258 g of a crude methanol extract. The extract was suspended in water (5 L) and partitioned with CH_2_Cl_2_ to afford a CH_2_Cl_2_‐soluble fraction (AM1, 130 g) and an aqueous fraction (AM2). The latter was subjected to Diaion HP‐20 column chromatography, employing a stepwise MeOH/H_2_O gradient. Sugars and salts were first removed by elution with water, followed by successive elution with 25%, 50%, 75%, and 100% MeOH, yielding four fractions (AM2A–AM2D). Among fractions AM1 and AM2A–AM2D, only fraction AM2C exhibited significant cytotoxicity against the HepG2, MCF‐7, and SK‐Lu‐1 cell lines and was therefore selected for further investigation. Fractionation of AM2C (the 75% MeOH) by normal‐phase silica gel column chromatography using CH_2_Cl_2_/MeOH (7:1, v/v) afforded four subfractions (AM2C1–AM2C4). Subsequent purification of AM2C2 by reversed‐phase YMC RP‐18 column chromatography with MeOH/H_2_O (1:3, v/v) yielded three subfractions (AM2C2A–AM2C2C).

Further purification of the subfractions by preparative HPLC enabled the isolation of compounds **2**, **3**, and **7–11**. Specifically, subfraction AM2C2A was purified using 35% ACN in water as the mobile phase to afford compound **2** (14.2 mg, *t*_R_ = 38.9 min). Purification of subfraction AM2C2B with 30% ACN yielded compounds **9** (8.3 mg, *t*_R_ = 44.3 min), **7** (8.9 mg, *t*_R_ = 51.1 min), and **3** (10.4 mg, *t*_R_ = 65.2 min). Similarly, HPLC purification of subfraction AM2C2C using 28% ACN afforded compounds **8** (8.6 mg, *t*_R_ = 51.3 min), **10** (11.4 mg, *t*_R_ = 56.5 min), and **11** (6.4 mg, *t*_R_ = 61.2 min). Fraction AM2C4 was subsequently fractionated by normal‐phase silica gel column chromatography using CH_2_Cl_2_/MeOH (10:1, v/v), yielding four subfractions (AM2C4A–AM2C4D). Further separation of AM2C4C under similar conditions with CH_2_Cl_2_/MeOH (8:1, v/v) afforded four subfractions (AM2C4C1–AM2C4C4). Subfraction AM2C4C3 was then subjected to reversed‐phase YMC RP‐18 column chromatography using MeOH/H_2_O (1:4, v/v), yielding three subfractions (AM2C4C3A–AM2C4C3C). Final purification of subfraction AM2C4C3B by HPLC with 27% ACN afforded compounds **1** (12.4 mg, *t*_R_ = 43.2 min) and **4** (10.1 mg, *t*_R_ = 48.7 min), whereas purification of subfraction AM2C4C3C with 25% ACN yielded compounds **6** (9.7 mg, *t*_R_ = 43.5 min) and **5** (7.8 mg, *t*_R_ = 49.9 min).

#### 4.3.1 Argymekoside A (**1**)

A white solid; mp. 221°C–223°C, ${\left[\alpha \right]}_{D}^{25}$: +36.0 (*c* 0.1, MeOH); IR (KBr) *γ*_max_: 3400, 2944, 1733, 1604, 1419, 1075, 1030 cm^−1^; HRESIMS *m/z* 833.4293 [M + Na]^+^, calculated for [C_42_H_66_O_15_Na]^+^: 833.4294, *Δ* = −0.1 ppm; *m/z* 809.4327 [M − H]^−^, calculated for [C_42_H_65_O_15_]^−^: 809.4329, *Δ* = −0.2 ppm. ^1^H NMR (CD_3_OD, 600 MHz) and ^13^C NMR (CD_3_OD, 150 MHz) data are shown in Table 1 (Figures S1–S10); ECD (MeOH) *θ*_(λ nm)_: +33.6_(202)_, −6.1_(225)_ mdeg.

#### 4.3.2 Argymekoside B (**2**)

A white solid; mp. 217°C–219°C, ${\left[\alpha \right]}_{D}^{25}$: +41.0 (*c* 0.1, MeOH); IR (KBr) *γ*_max_: 3447, 2944, 1691, 1638, 1463, 1077, 1021 cm^−1^; HRESIMS *m/z* z 811.4478 [M + H]^+^, calcd for [C_42_H_67_O_15_]^+^: 811.4475, *Δ* = +0.4 ppm; ^1^H NMR (CD_3_OD, 600 MHz) and ^13^C NMR (CD_3_OD, 150 MHz) data are shown in Table 1 (Figures S11–S20); ECD (MeOH) *θ*_(λ nm)_: +23.5_(200)_, −8.3_(218)_ mdeg.

#### 4.3.3 Argymekoside C (**3**)

A white solid; mp. 228°C–230°C, ${\left[\alpha \right]}_{D}^{25}$: +38.0 (*c* 0.1, MeOH); IR (KBr) *γ*_max_: 3429, 2942, 1692, 1605, 1416, 1076, 1030 cm^−1^; HRESIMS *m/z* 649.3956 [M + H]^+^, calcd for [C_36_H_57_O_10_]^+^: 649.3946, *Δ* = +1.5 ppm. ^1^H NMR (DMSO‐d_6_, 600 MHz) and ^13^C NMR (DMSO‐d_6_, 150 MHz) data are shown in Table 1 (Figures S21–S30); ECD (MeOH) *θ*_(λ nm)_: +20.5_(200)_, −8.3_(222)_ mdeg.

#### 4.3.4 Theoretical Calculation of ECD Spectra for Compounds **1–3**

Conformational searches and geometric equilibrium were carried out using the Spartan18 program (Wavefunction Inc., Irvine, CA, USA). The isomer (3*S*,5*R*,8*R*,9*R*,10*R*,13*S*,17*R*,18*S*,21*R*) was generated and submitted to conformational searches using the Molecular Mechanics MMFF, and then equilibrium geometry was performed with the semi‐empirical PM3 set. The initial stable conformers with a Boltzmann distribution over 1% were selected and optimized by DFT calculations at the B3LYP/6‐31G(d,p) basic set and IEFPCM models for the solvent of methanol using the Gaussian 16 program (Gaussian Inc., Wallingford, CT, USA). The optimized conformers were then subjected to TDDFT calculations at the B3LYP/6‐31G(d,p) level in the presence of methanol with IEFPCM models. The ECD spectra at 30 excited states for each conformer were collected and summed to obtain theoretical ECD spectra of the calculated stereoisomer using SpecDis v1.71 software (University of Wuerzburg, Wuerzburg, Germany). The half‐band was taken at *ζ* = 0.35 eV with a blueshifted UV correction by 12 nm. The calculated ECD spectrum of its enantiomer was composed of a mirror‐image curve

#### 4.3.5 Acid Hydrolysis and Confirmation of Monosaccharide

Compounds **1–3** (5.0 mg each) were separately hydrolyzed with 1.0 M HCl in dioxane–H_2_O (1:1, v/v, 1.0 mL) at 80°C for 3 h. After cooling, the solutions were neutralized with Ag_2_CO_3_, and the precipitated AgCl was removed. The filtrates were concentrated under N_2_, redissolved in H_2_O (1.0 mL), and extracted with CHCl_3_ (3 × 1.0 mL). The aqueous layers were evaporated to dryness, and the monosaccharides were purified by preparative TLC on silica gel 60 F_254_ using EtOAc/i‐PrOH/acetone/H_2_O (20:10:7:6, v/v/v/v). Comparison with authentic standards identified glucose (*R*_f_ = 0.41) and glucuronic acid (*R*_f_ = 0.08). Optical rotation further confirmed D‐glucose (${\left[\alpha \right]}_{D}^{25}$: +46.8, *c* 0.08, H_2_O) in **1** and **2**, and D‐glucuronic acid (${\left[\alpha \right]}_{D}^{25}$: +14.8, *c* 0.07, H_2_O) in **1–3**.

#### 4.3.6 Cytotoxicity Assay

SK‐LU‐1 (human lung adenocarcinoma cell line; RRID:CVCL_0629), HepG2 (human hepatocellular carcinoma cell line; RRID: CVCL_0027), and MCF‐7 (human breast cancer cell line; RRID: CVCL_0031) cells were obtained from Milan University (Italy) and Long Island University (USA), respectively, and cultured in DMEM supplemented with FBS, trypsin‐EDTA, L‐glutamine, sodium pyruvate, NaHCO_3_, and penicillin/streptomycin at 37°C under 5% CO_2_. Cytotoxicity was evaluated by the Sulforhodamine B (SRB) assay according to a previously reported method [30, 31]. Briefly, cells were treated with or without the test compounds in 96‐well plates for 72 h, followed by SRB staining and OD measurement at 540 nm. Cell viability was calculated relative to the vehicle control, and results were expressed as percentage cell death. **Ellipticine** was used as a positive control. All experiments were performed in triplicate, and IC_50_ values were calculated using TableCurve 2D v4.

#### 4.3.7 Statistical Analyses

Values for cytotoxic activities are presented as mean±SD (*n* = 3). Data analysis were performed by Microsoft Excel using Student's *t*‐test and one‐way analysis of variance (ANOVA). Significance was detected between the treated samples versus control at the level of *p* < 0.05.

## Author Contribution

Methodology: Tran Thi Ngoc Hanh. Investigation: Tran Thi Ngoc Hanh, Nguyen Thi Thu Hien, Pham Van Huyen and Duong Thi Hai Yen. Methodology: Nguyen Thi Thu Hien and Duong Thi Hai Yen. Validation: Pham Van Huyen. Formal analysis: Nguyen Huu Huong Duyen and Nguyen Thi Dieu Thuan. Data curation: Bui Huu Tai and Phan Van Kiem. Writing – review & editing: Nguyen Huu Toan Phan, Nguyen Thi Dieu Thuan, Bui Huu Tai and Phan Van Kiem. Writing – original draft: Bui Huu Tai and Phan Van Kiem. Supervision: Nguyen Huu Toan Phan and Phan Van Kiem. Project administration: Nguyen Huu Toan Phan.

## Funding

This research is funded by Vietnam National Foundation for Science and Technology Development (NAFOSTED) under grant number 104.01‐2023.96.

## Conflicts of Interest

The authors declare no conflicts of interest.

## Supporting information




**Supporting File**: cbdv71814‐sup‐0001‐SuppMat.pdf
HR‐ESI‐MS, IR, NMR, and ECD spectra of new compounds (**1**–**3**) and NMR data of compounds (**4**–**11**) are available in the Supporting Information.
Supporting Information for this article is available on the WWW under https://doi.org/10.1002/8694950.
Additional references cited within the Supporting Information [20, 31].

## Data Availability

The data that support the findings of this study are available in the Supporting Information of this article.
