# Supplementary material for: Argymekosides A‐C, Three Undescribed 21α‐Hydroxyoleanolic Acid Glycosides From Argyreia mekongensis and Their Cytotoxic Activity
Source: Chem Biodivers. 2026 Oct 12;23(10):e71814. doi: 10.1002/cbdv.71814 (PMC13659019; doi:10.1002/cbdv.71814)
Supplement: Supplementary file 1 [file CBDV-23-e71814-s001.pdf]

SUPPLEMENTARY MATERIAL

Argymekosides A-C, Three Undescribed 21*α*-Hydroxyoleanolic Acid Glycosides from *Argyreia mekongensis* and Their Cytotoxic Activity

Tran Thi Ngoc Hanh[1,2], Nguyen Huu Toan Phan*[,1,2], Nguyen Thi Thu Hien[1], Pham Van Huyen[1], Nguyen Huu Huong Duyen[1], Nguyen Thi Dieu Thuan[1], Duong Thi Hai Yen[3], Bui Huu Tai[3], and Phan Van Kiem*[,2,3]

[1] Institute of Life Sciences, Vietnam Academy of Science and Technology (VAST), 9/621 Vo Nguyen Giap, Linh Xuan Ward, Ho Chi Minh City, Vietnam.

[2] Faculty of Chemistry, Graduate University of Science and technology, VAST, 18 Hoang Quoc Viet, Nghia Do, Hanoi 10072, Vietnam.

[3] Department of Struture Research, Institute of Chemistry, VAST, 18 Hoang Quoc Viet, Nghia Do, Hanoi 10072, Viet Nam

*Corresponding authors:

Phan Van Kiem, Prof. Ph.D.

Department of Struture Research, Institute of Chemistry, VAST, 18 Hoang Quoc Viet, Nghia Do, Hanoi 10072, Viet Nam

Tel.: +84-4-3791-7053 Fax: +84-4-3791-7054 E-mail: phankiem@yahoo.com

Content

Experimental		4
General		4
Table S1	Table S1. The cytotoxic activity of compounds **1-11** against three cancer cell lines	5
Figure S1	HR-ESI-MS of compound **1**	7
Figure S2	IR spectrum of compound **1**	7
Figure S3	^{1}H NMR spectra of compound **1** in CD_3OD	8
Figure S4	^{13}C NMR spectra of compound **1** in CD_3OD	9
Figure S5	HSQC spectra of compound **1**in CD_3OD	10
Figure S6	HMBC spectra of compound **1** in CD_3OD	11
Figure S7	Expanded HMBC spectra of compound **1** in CD_3OD	12
Figure S8	COSY spectrum of compound **1** in CD_3OD	13
Figure S9	NOESY spectrum of compound **1** in CD_3OD	14
Figure S10	ECD spectrum of compound **1**	14
Figure S11	HR-ESI-MS of compound **2**	15
Figure S12	IR spectrum of compound **2**	15
Figure S13	^{1}H NMR spectra of compound **2** in CD_3OD	16
Figure S14	^{13}C NMR spectra of compound **2** in CD_3OD	17
Figure S15	HSQC spectra of compound **2** in CD_3OD	18
Figure S16	HMBC spectra of compound **2** in CD_3OD	19
Figure S17	Expanded HMBC spectra of compound **2** in CD_3OD	20
Figure S18	COSY spectrum of compound **2** in CD_3OD	21
Figure S19	NOESY spectrum of compound **2** in CD_3OD	22
Figure S20	ECD spectrum of compound **2**	22
Figure S21	HR-ESI-MS of compound **3**	23
Figure S22	IR spectrum of compound **3**	23
Figure S23	^{1}H NMR spectra of compound **3** in DMSO-d_6	24
Figure S24	^{13}C NMR spectra of compound **3** in DMSO-d_6	25
Figure S25	HSQC spectra of compound **3** in DMSO-d_6	26
Figure S26	HMBC spectra of compound **3** in DMSO-d_6	27
Figure S27	Expanded HMBC spectra of compound **3** in DMSO-d_6	28
Figure S28	COSY spectrum of compound **3** in DMSO-d_6	29
Figure S29	NOESY spectrum of compound **3** in DMSO-d_6	30
Figure S30	ECD spectrum of compound **3**	30
Figure S31	^{1}H and ^{13}C NMR spectra for compound **4** in CD_3OD	31
Figure S32	^{1}H and ^{13}C NMR spectra for compound **5** in CD_3OD	32
Figure S33	^{1}H and ^{13}C NMR spectra for compound **6** in CD_3OD	33
Figure S34	^{1}H and ^{13}C NMR spectra for compound **7** in CD_3OD	34
Figure S35	^{1}H and ^{13}C NMR spectra for compound **8** in CD_3OD	35
Figure S36	^{1}H and ^{13}C NMR spectra for compound **9** in CD_3OD	36
Figure S37	^{1}H and ^{13}C NMR spectra for compound **10** in CD_3OD	37
Figure S38	^{1}H and ^{13}C NMR spectra for compound **11** in CD_3OD	38
Table S2	^{1}H and ^{13}C NMR spectroscopic data for compound **4** in CD_3OD	39
Table S3	^{1}H and ^{13}C NMR spectroscopic data for compound **5** in CD_3OD	40
Table S4	^{1}H and ^{13}C NMR spectroscopic data for compound **6** in CD_3OD	41
Table S5	^{1}H and ^{13}C NMR spectroscopic data for compound **7** in CD_3OD	42

Table S6	^1H and ^{13}C NMR spectroscopic data for compound **8** in CD_3OD	43
Table S7	^1H and ^{13}C NMR spectroscopic data for compound **9** in CD_3OD	44
Table S8	^1H and ^{13}C NMR spectroscopic data for compound **10** in CD_3OD	45
Table S9	^1H and ^{13}C NMR spectroscopic data for compound **11** in CD_3OD	46

Experimental
General

The optical rotations were measured on a Jasco P2000-polarimeter. The HR-ESI-MS was measured on an Agilent 6530 Accurate Mass Q-TOF LC/MS. The NMR spectra were recorded on a Bruker 600 MHz spectrometer. The preparative HPLC were run on an Agilent-1100 system including quaternary pump, DAD detector, autosampler, and preparative HPLC column YMC J'sphere ODS-H80 (4 µm, 20 × 250 mm). Isocratic mobile phase with the flow rate of 3 mL/min was used in pre-HPLC. The compound was monitored at wavelengths of 205, 230, 254, and 280 nm. Flash column chromatography was run using silica gel, reversed phase C-18, and diaion HP-20 resins as stationary phase. Thin layer chromatography was carried out on pre-coated silica gel 60 F_{254} and RP-18 F_{254S} plates. The spots were detected by spraying with aqueous solution of H_2SO_4 5% followed by heating with a heat gun.

Table S1. The cytotoxic activity of compounds **1-11** against three cancer cell lines

Compounds	Concentration (μM)	HepG2		MCF-7		SK-LU-1	
		Inhibition (%)	SD	Inhibition (%)	SD	Inhibition (%)	SD
**1**	100	35.49	1.34	34.68	1.64	34.67	1.87
	20	14.58	1.01	13.47	1.23	16.55	1.41
	4	12.27	1.08	10.89	0.96	11.01	1.12
	0.8	7.64	0.32	6.73	0.28	7.98	0.30
	**IC_{50}**	**>100**	-	**>100**	-	**>100**	-
**2**	100	35.49	1.34	34.68	1.64	34.67	1.87
	20	14.58	1.01	13.47	1.23	16.55	1.41
	4	12.27	1.08	10.89	0.96	11.01	1.12
	0.8	7.64	0.32	6.73	0.28	7.98	0.30
	**IC_{50}**	**>100**	-	**>100**	-	**>100**	-
**3**	100	24.88	1.84	23.17	2.21	24.69	2.16
	20	17.48	1.55	15.91	1.39	17.02	1.24
	4	11.69	0.95	11.29	1.03	12.03	1.06
	0.8	6.37	0.47	5.61	0.57	6.10	0.51
	**IC_{50}**	**>100**	-	**>100**	-	**>100**	-
**4**	100	99.77	1.98	95.51	1.82	99.06	2.19
	20	43.02	1.82	35.70	1.88	31.99	1.69
	4	18.17	1.17	17.43	1.08	21.65	1.45
	0.8	7.75	0.23	5.54	0.46	4.36	0.49
	**IC_{50}**	**26.73**	**2.05**	**33.69**	**2.42**	**35.53**	**2.45**
**5**	100	99.50	1.60	98.01	1.89	98.09	2.15
	20	95.81	1.37	96.16	1.69	95.02	1.23
	4	35.88	1.67	35.51	1.46	33.45	1.26
	0.8	14.39	1.34	8.24	0.89	9.81	0.78
	**IC_{50}**	**5.06**	**0.39**	**5.70**	**0.40**	**5.79**	**0.36**
**6**	100	61.81	1.81	53.28	1.40	48.35	1.69
	20	21.64	1.80	17.23	1.56	15.52	1.12
	4	9.72	0.98	8.12	0.76	3.06	0.34
	0.8	2.97	0.17	2.58	0.14	2.01	0.22
	**IC_{50}**	**72.03**	**4.69**	**91.51**	**3.95**	**>100**	-
**7**	100	22.57	1.04	21.46	1.62	20.67	1.80
	20	12.38	1.28	13.92	1.60	16.44	1.24
	4	6.71	0.57	6.89	0.61	10.15	1.04
	0.8	2.43	0.26	3.69	0.34	1.92	0.15
	**IC_{50}**	**>100**	-	**>100**	-	**>100**	-
**8**	100	21.41	2.47	20.40	1.97	26.42	1.96
	20	18.06	1.38	15.51	1.64	19.77	1.44
	4	8.80	0.78	6.80	0.67	11.01	1.03
	0.8	5.09	0.57	2.97	0.23	6.10	0.68
	**IC_{50}**	**>100**	-	**>100**	-	**>100**	-
	100	24.23	2.02	25.64	1.51	25.90	1.58

**9**	20	9.26	0.82	9.87	0.90	14.40	0.93
	4	6.56	0.58	8.38	0.82	6.99	0.58
	0.8	4.48	0.31	4.76	0.11	3.35	0.35
	**IC_{50}**	**>100**	**-**	**>100**	**-**	**>100**	**-**
**10**	100	29.55	1.24	24.75	2.10	24.84	1.74
	20	12.02	1.24	16.30	1.57	17.08	1.71
	4	5.28	0.79	7.13	0.68	6.28	0.65
	0.8	1.69	0.11	1.39	0.15	3.88	0.29
	**IC_{50}**	**>100**	**-**	**>100**	**-**	**>100**	**-**
**11**	100	18.04	1.81	15.13	1.51	17.16	1.54
	20	7.10	0.35	6.46	0.59	11.52	0.94
	4	4.17	0.32	4.12	0.23	6.60	0.63
	0.8	2.47	0.25	1.71	0.15	2.98	0.27
	**IC_{50}**	**>100**	**-**	**>100**	**-**	**>100**	**-**
**Ellipticine***	100	98.58	1.98	96.45	2.10	89.95	2.35
	20	86.46	1.74	80.09	1.56	75.77	1.99
	4	51.86	1.19	51.61	0.98	49.58	1.09
	0.8	22.92	1.26	21.97	1.03	21.22	0.95
	**IC_{50}**	**0.32**	**0.03**	**0.35**	**0.02**	**0.39**	**0.02**

*Ellipticine was used as positive control

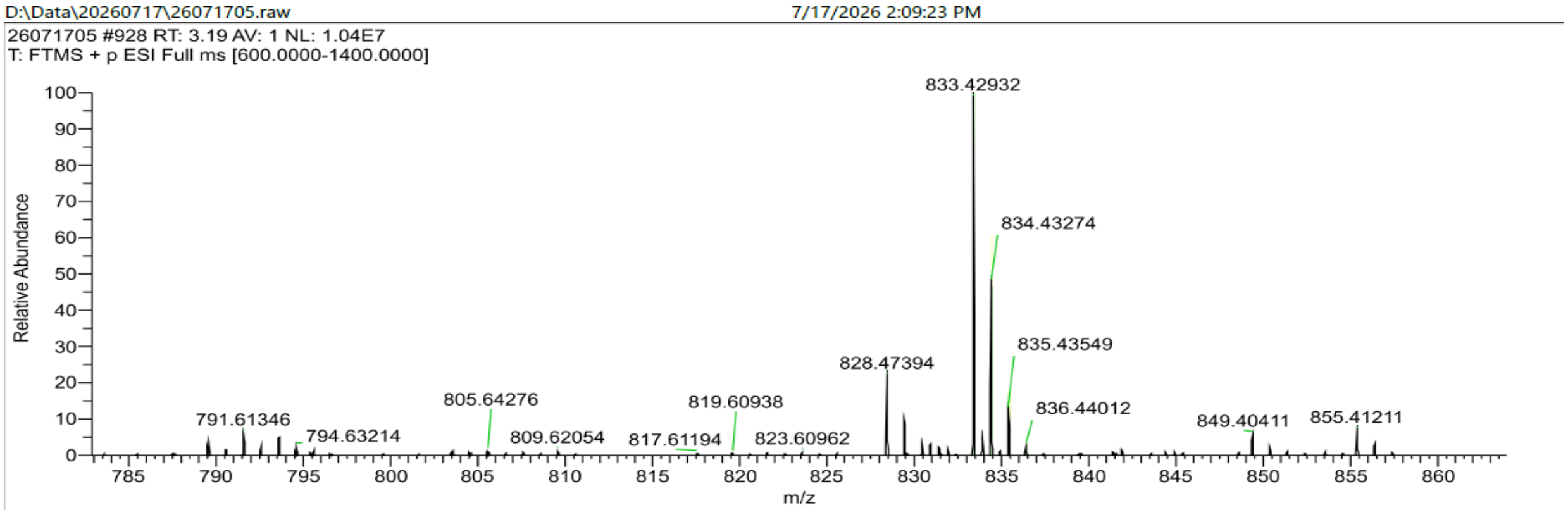

Peak Mass	Display For...	S Fit	RDB	Delta [ppm]	Theo. mass	Rank	Combined S...	# Matched I...	# Missed Iso.	MS Cov. [%]	Pattern Cov....	MSMS Matc.
833.42932	$C_{42}H_{66}O_{15}{}^{23}Na$	66.8209256842492	9.50	-0.09	833.42939	1	94.45	5	0	95.98	100	(Collection)

(+)-HRESIMS *m/z* 833.4293 $[M+Na]^+$, calculated for $[C_{42}H_{66}O_{15}Na]^+$: 833.4294, Δ = -0.1 ppm

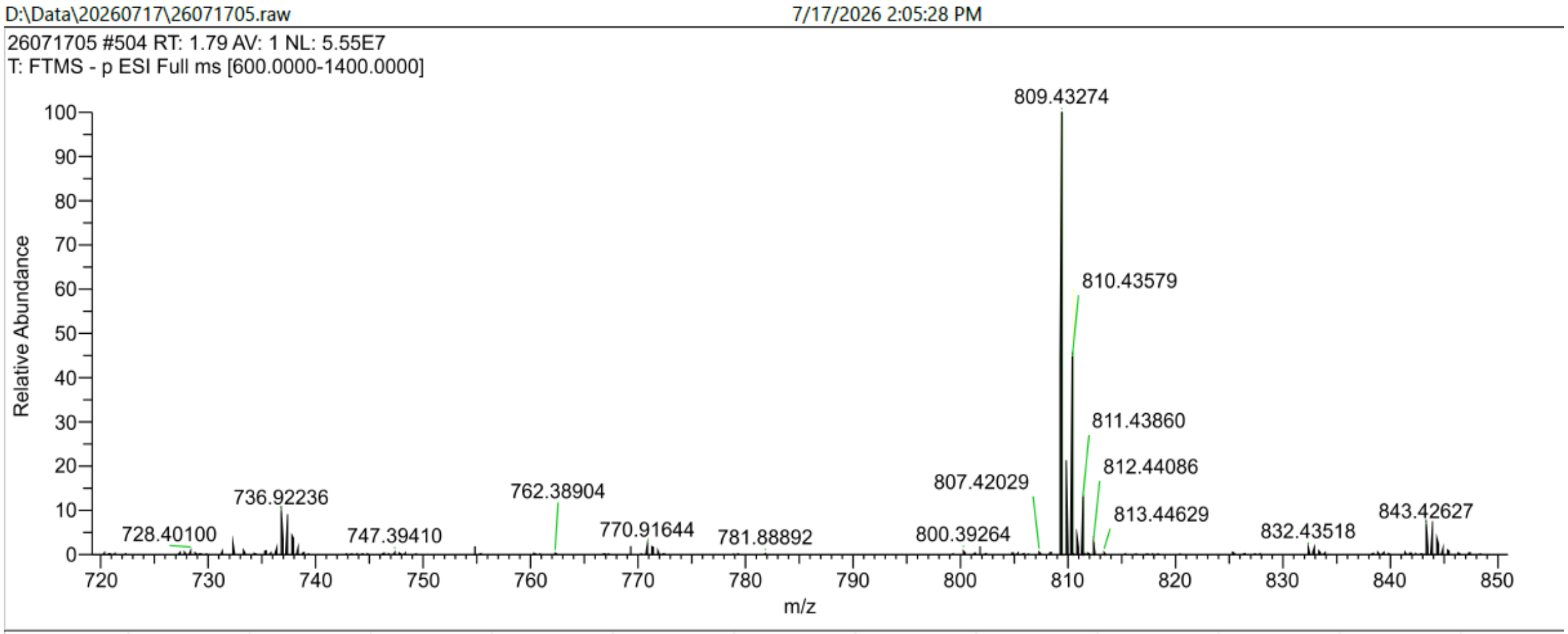

Peak Mass	Display For...	S Fit	RDB	Delta [ppm]	Theo. mass	Rank	Combined S...	# Matched I...	# Missed Iso.	MS Cov. [%]	Pattern Cov....	MSMS Matc.
809.43274	$C_{42}H_{65}O_{15}$	67.00759658334	10.50	-0.19	809.43289	1	85.74	5	0	86.78	100	(Collection)

(-)-HRESIMS *m/z* 809.4327 $[M-H]^-$, calculated for $[C_{42}H_{65}O_{15}]^-$: 809.4329, Δ = -0.2 ppm.

Figure S1. HR-ESI-MS of compound **1**

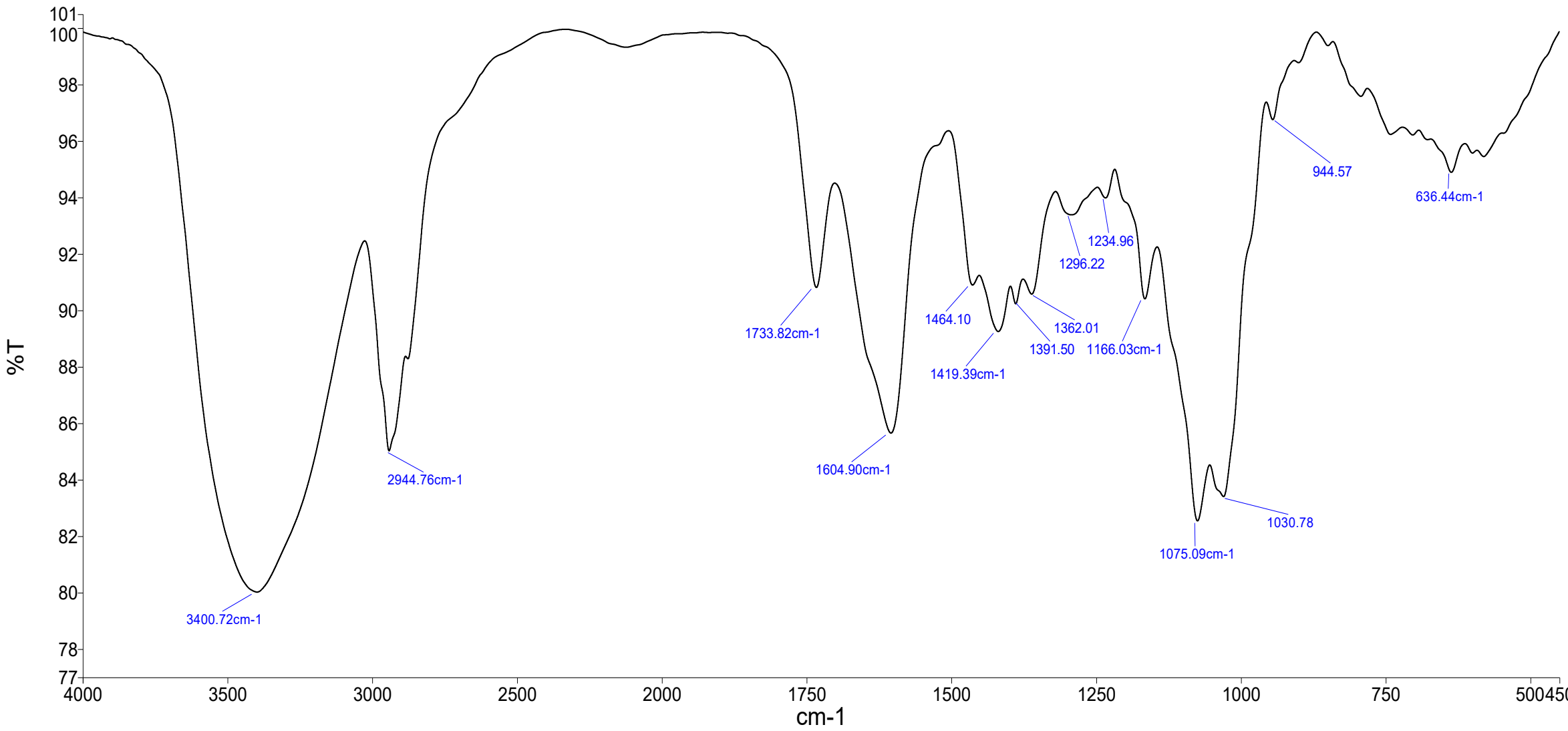

Figure S2. IR spectrum of compound **1**

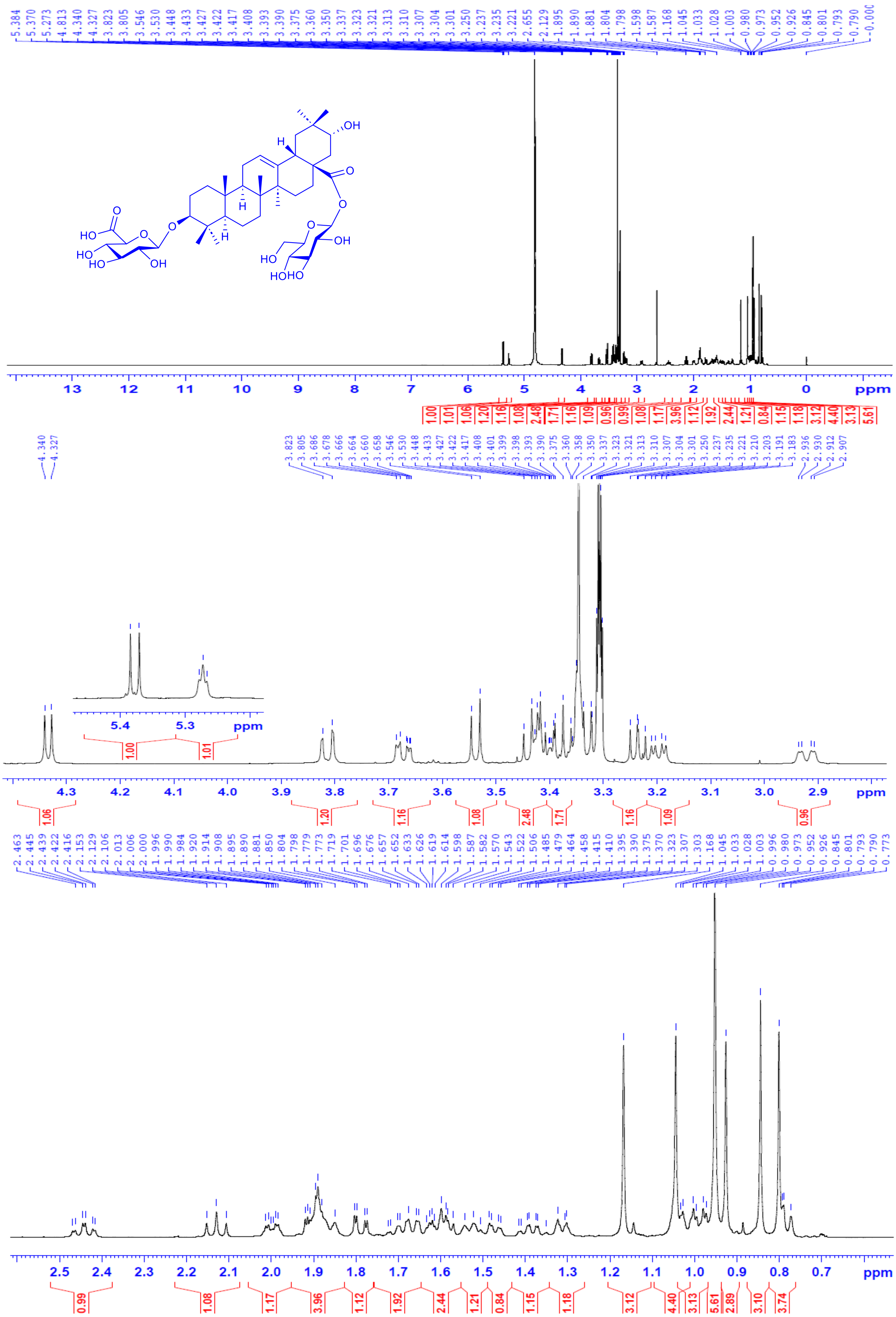

Figure S3. ^{1}H-NMR spectra of compound **1** in CD_3OD

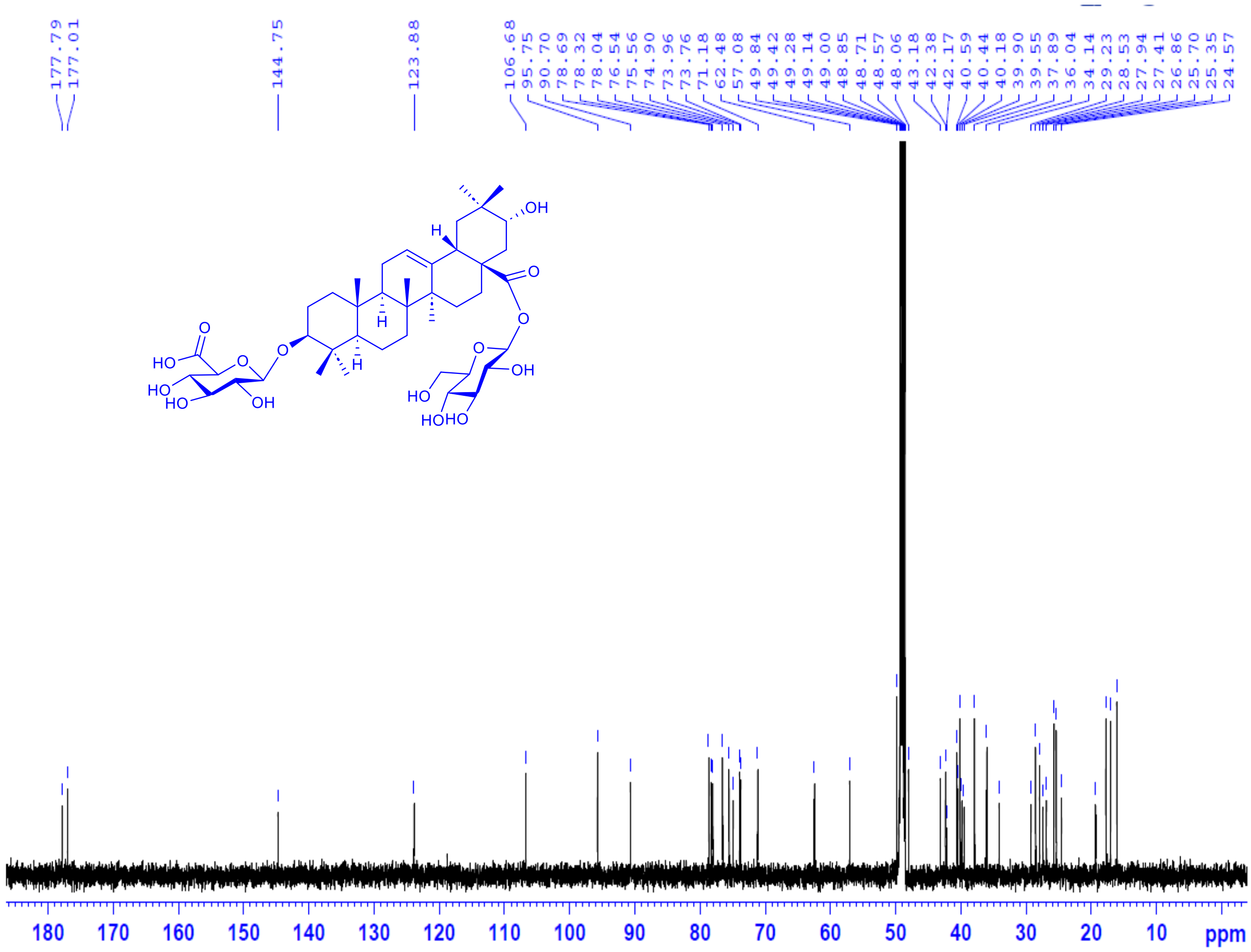

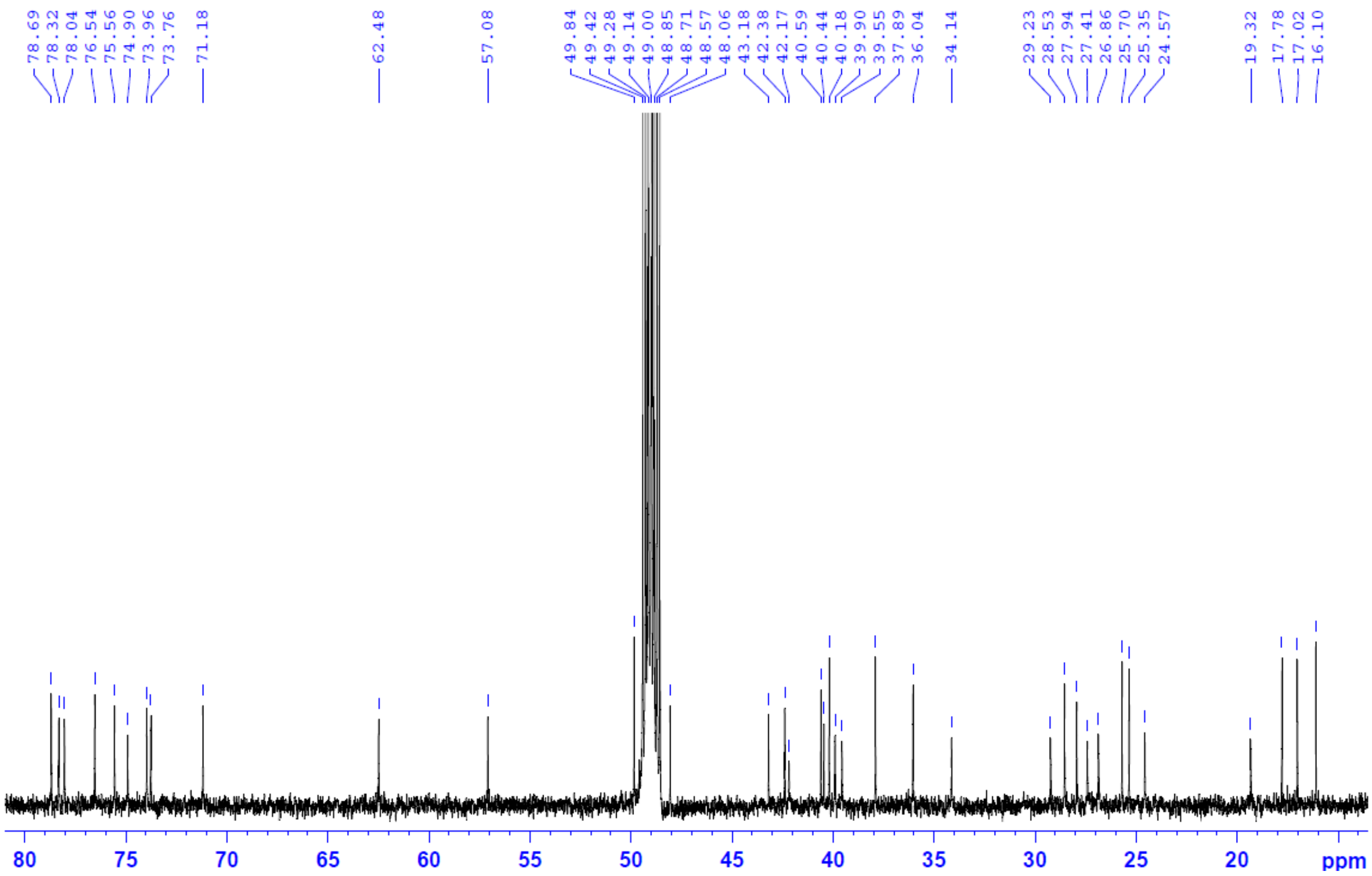

Figure S4. ^{13}C-NMR spectra of compound **1** in CD_3OD

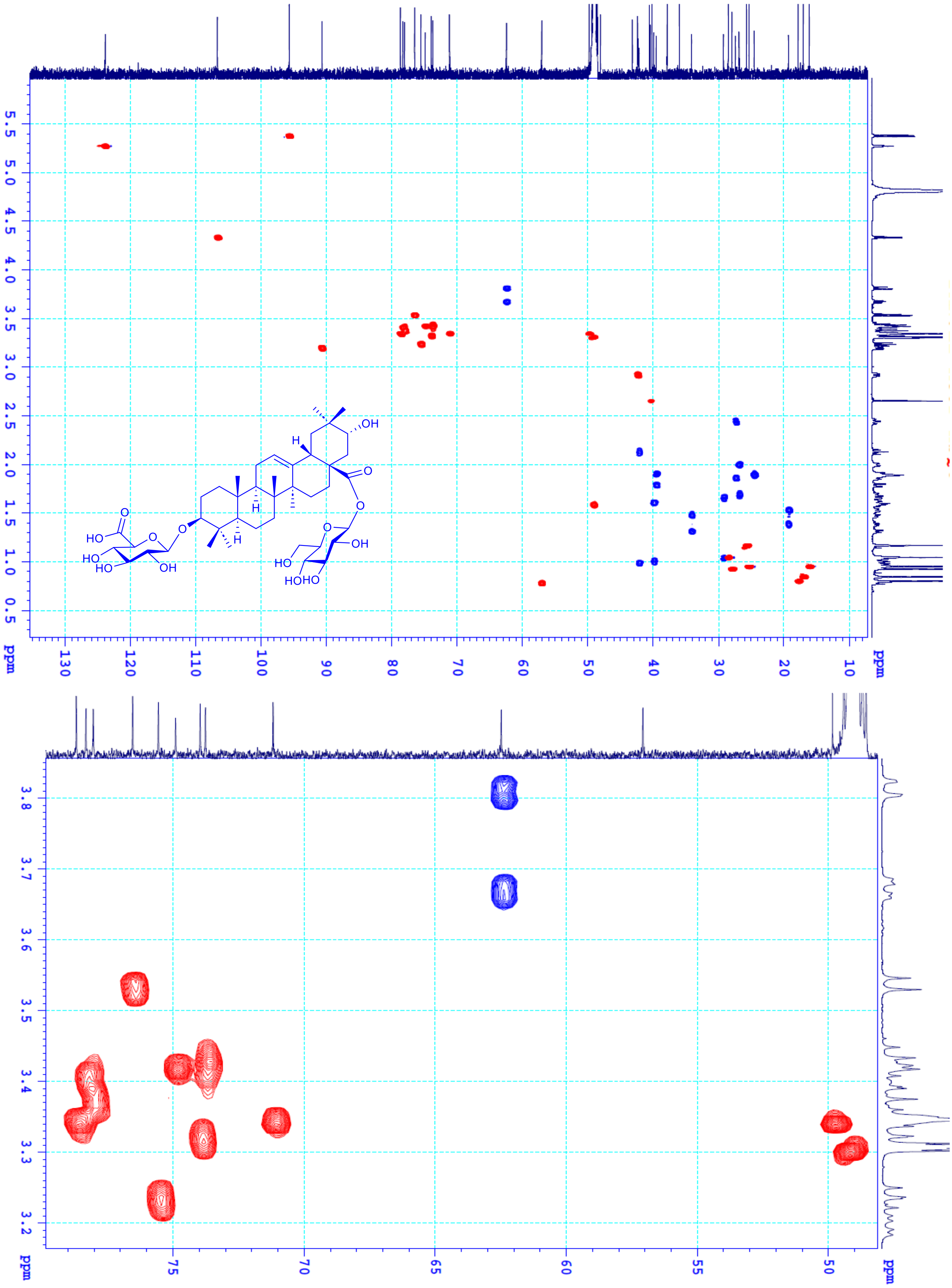

Figure S5. HSQC spectra of compound **1**

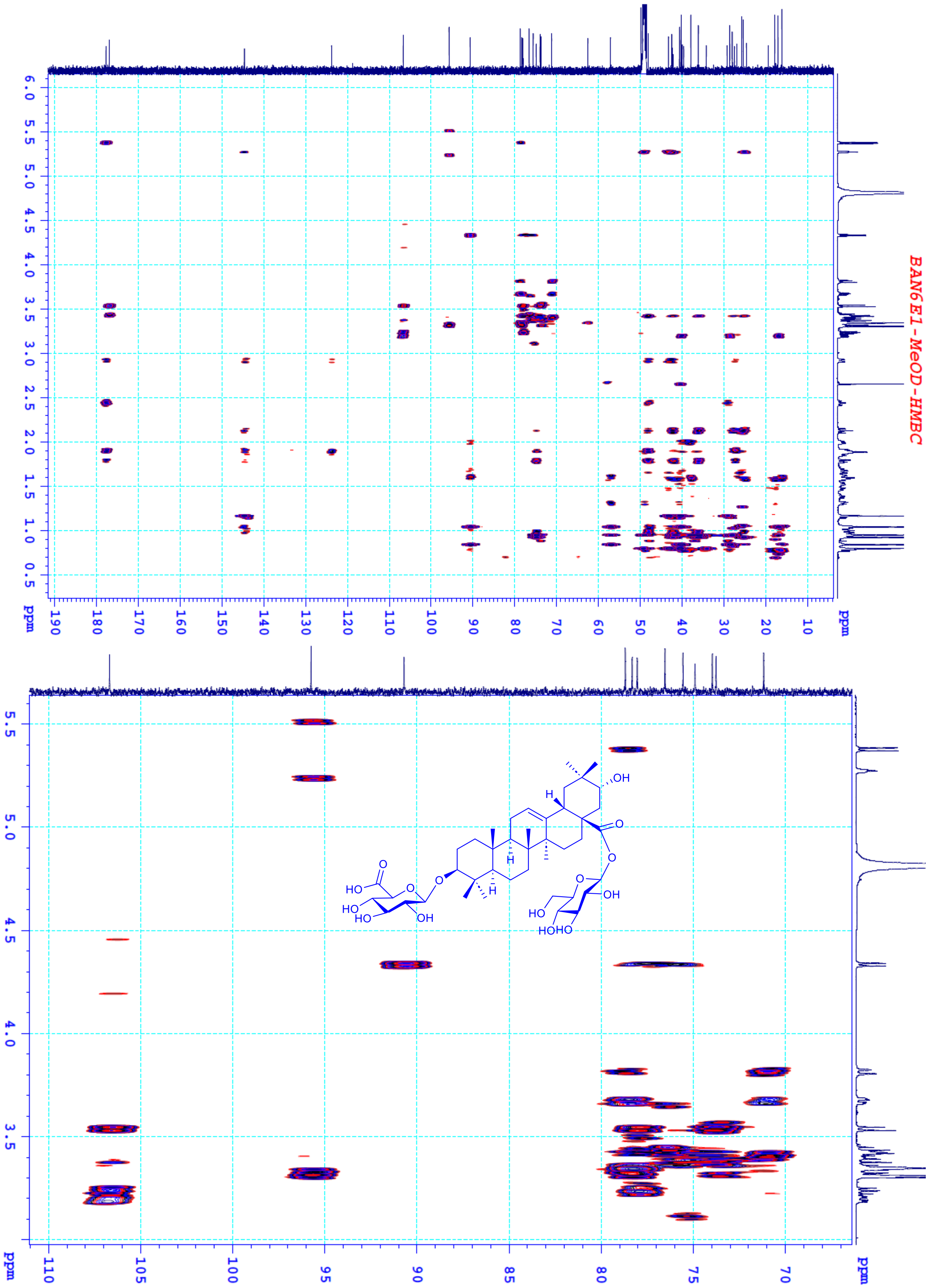

Figure S6. HMBC spectra of compound **1**

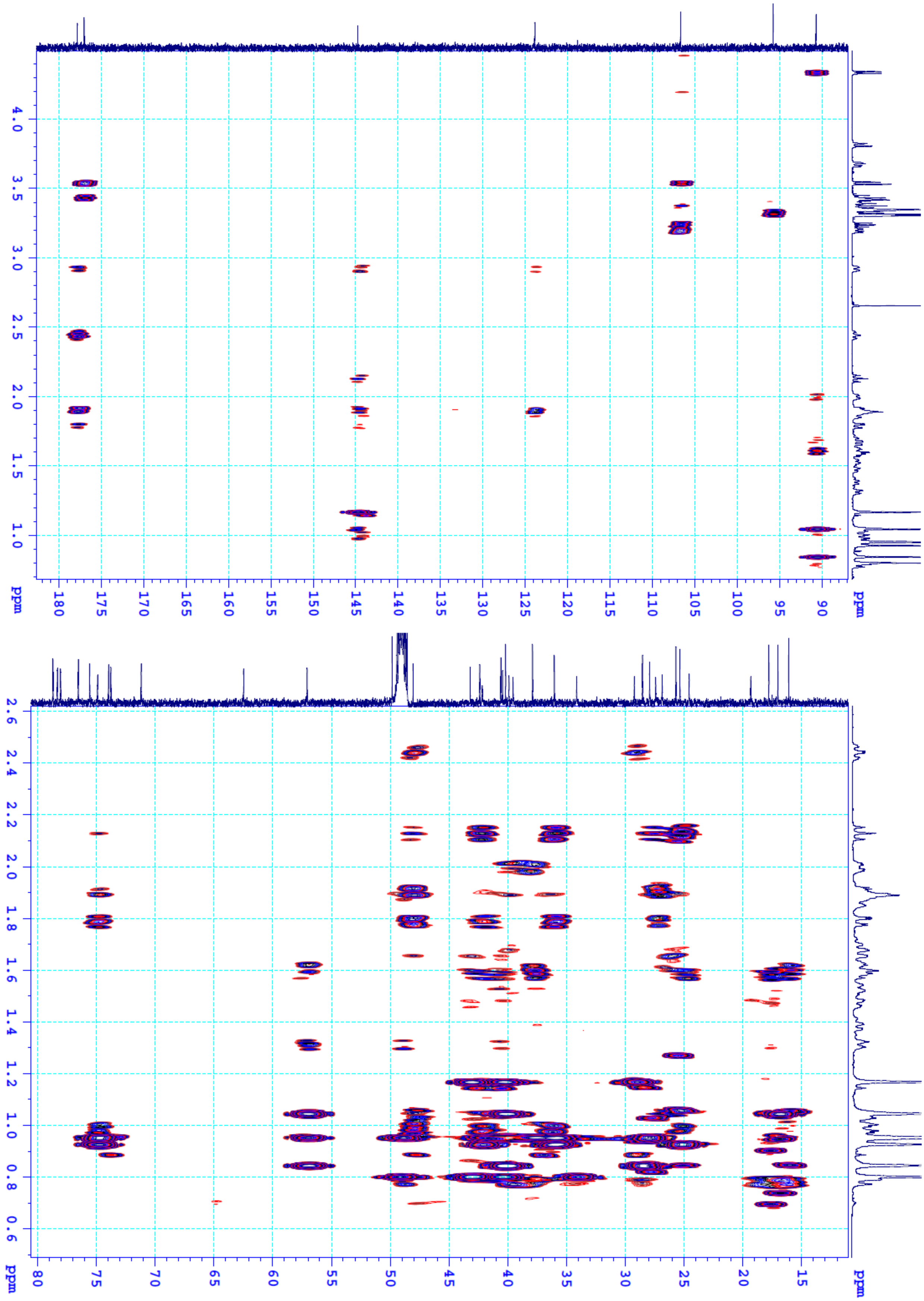

Figure S7. Expanded HMBC spectra of compound **1**

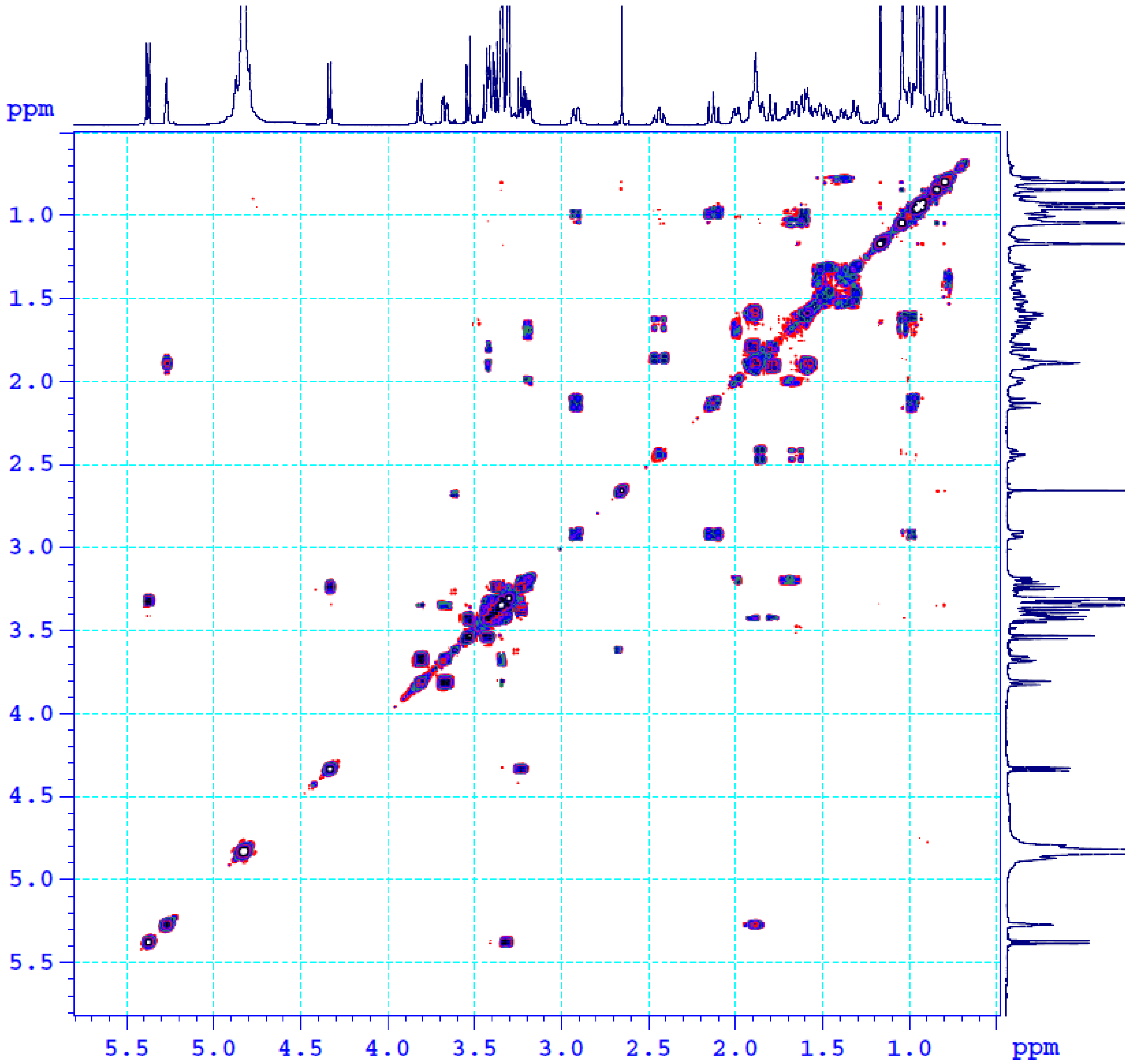

Figure S8. COSY spectrum of compound **1**

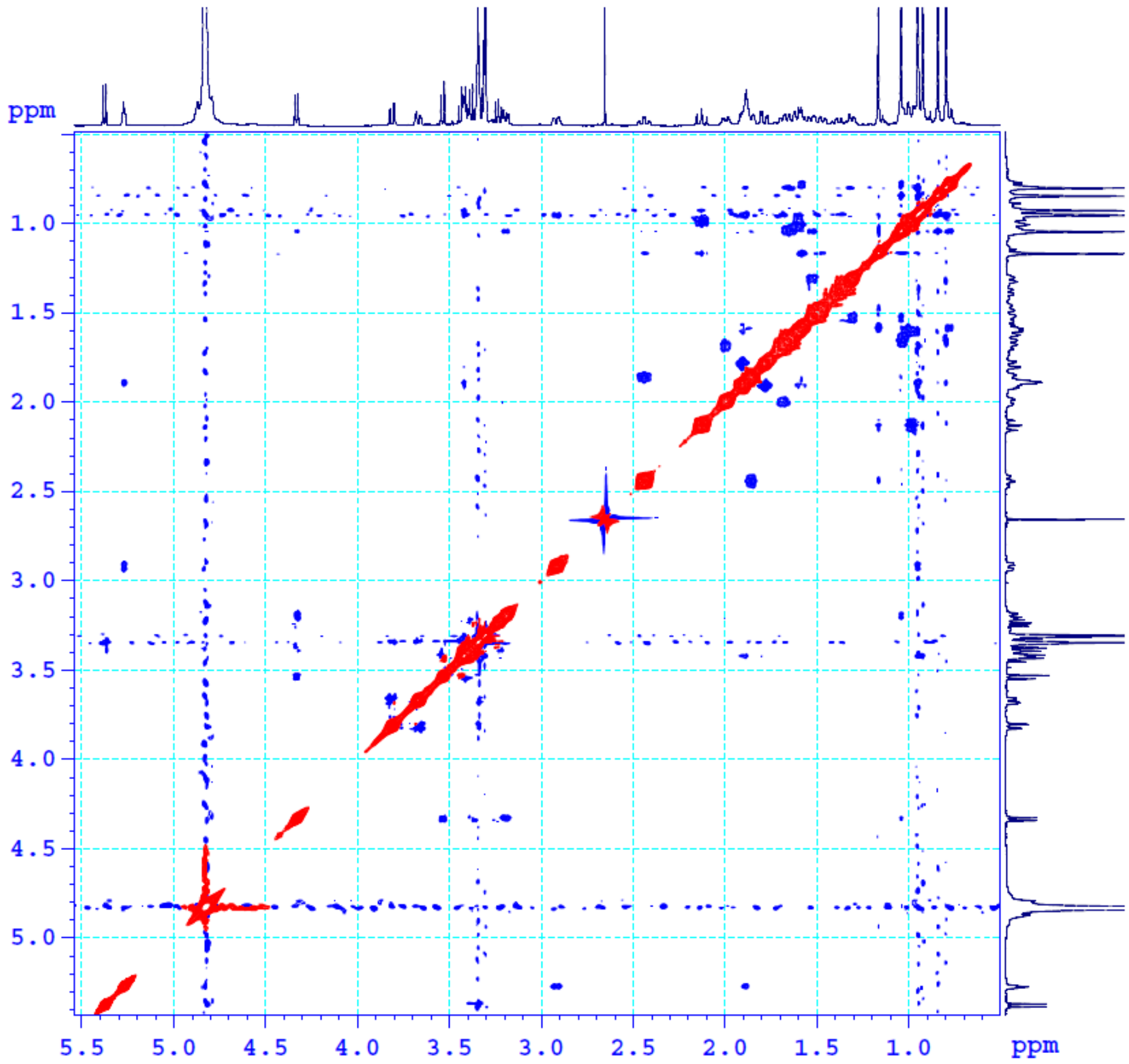

Figure S9. NOESY spectrum of compound **1**

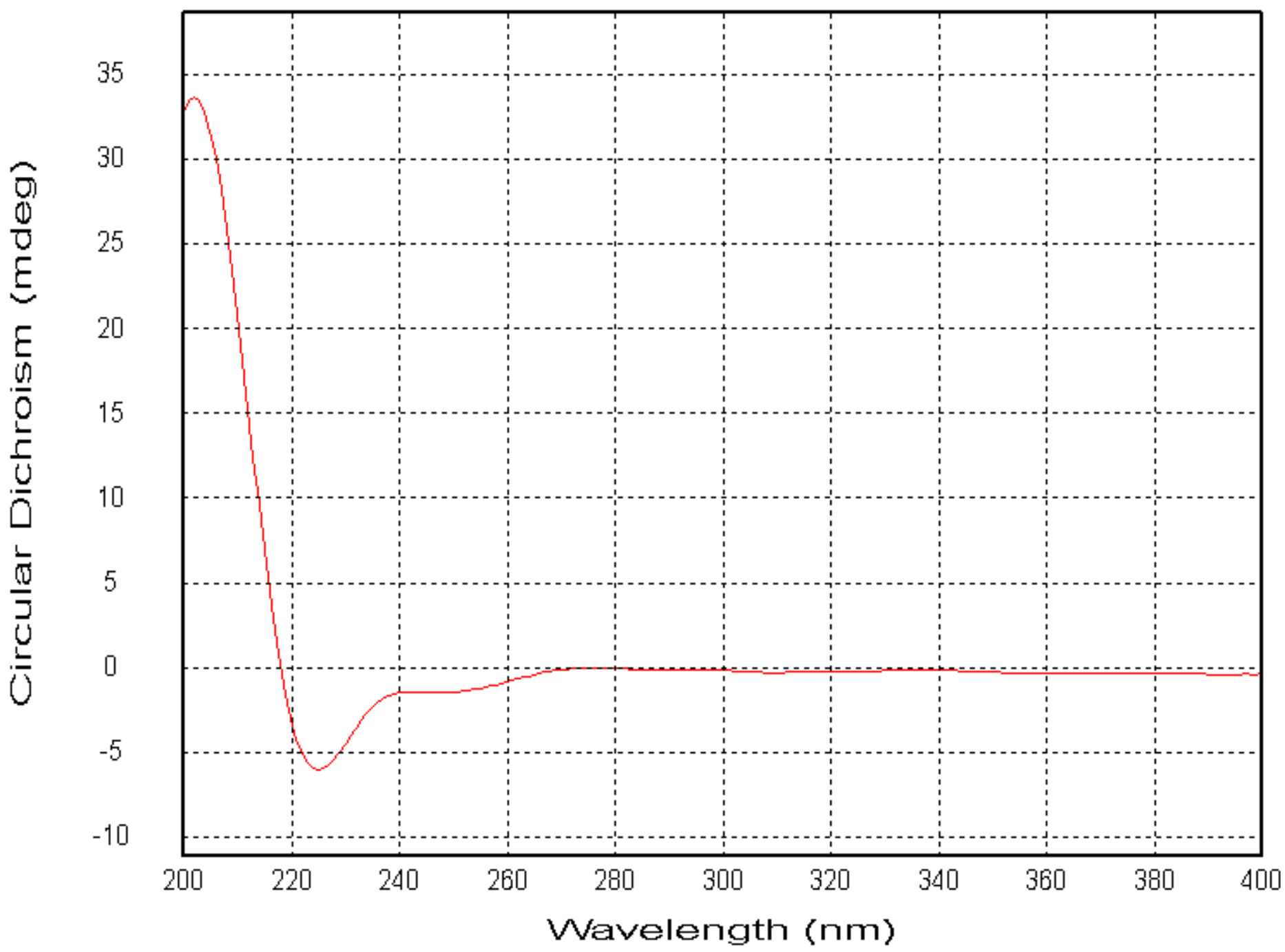

Figure S10. ECD spectrum of compound **1**

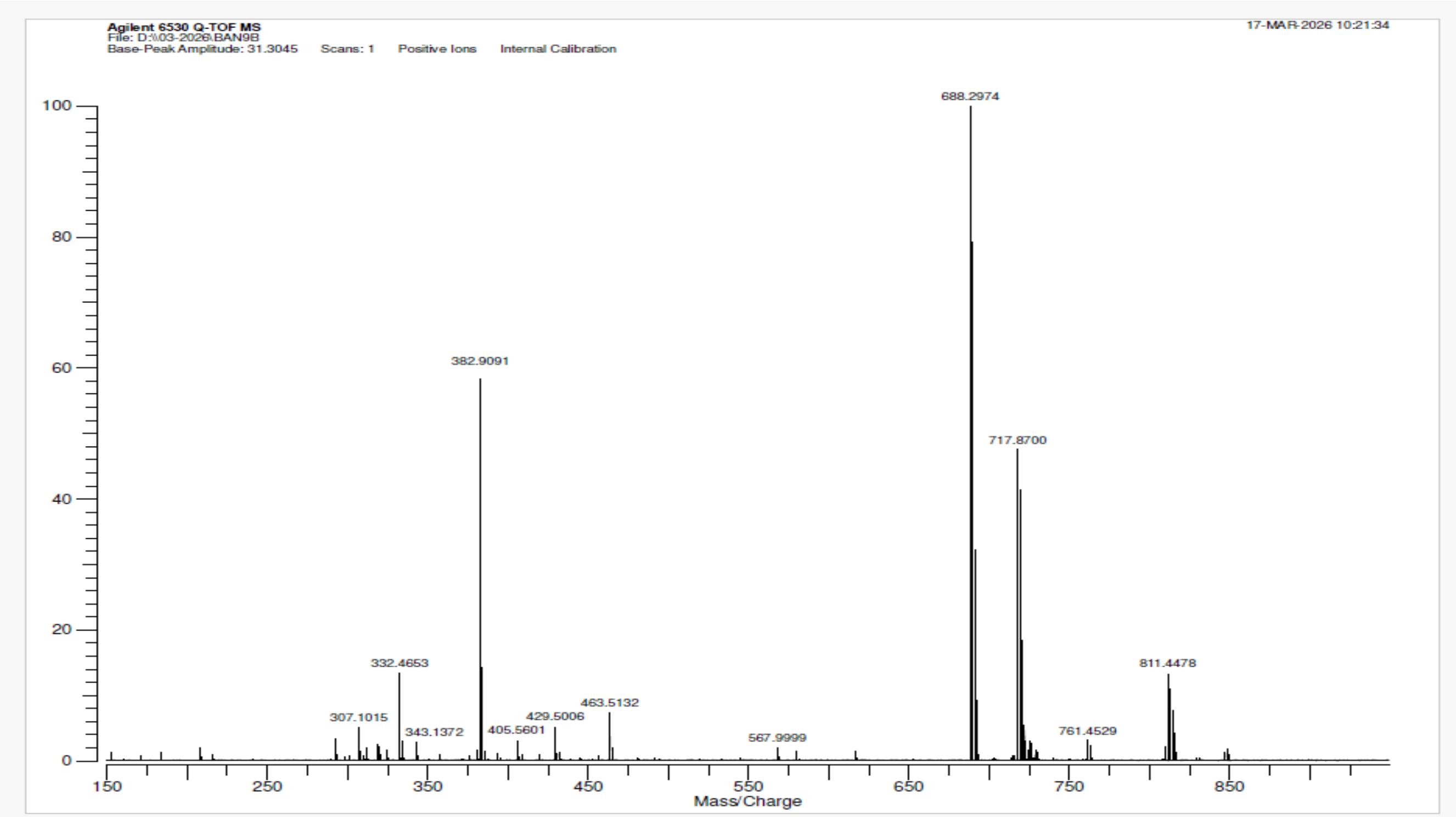

HRESIMS *m/z* z 811.4478 $[M+H]^+$, calcd for $[C_{42}H_{67}O_{15}]^+$: 811.4475, Δ = +0.4 ppm

Figure S11. HR-ESI-MS of compound **2**

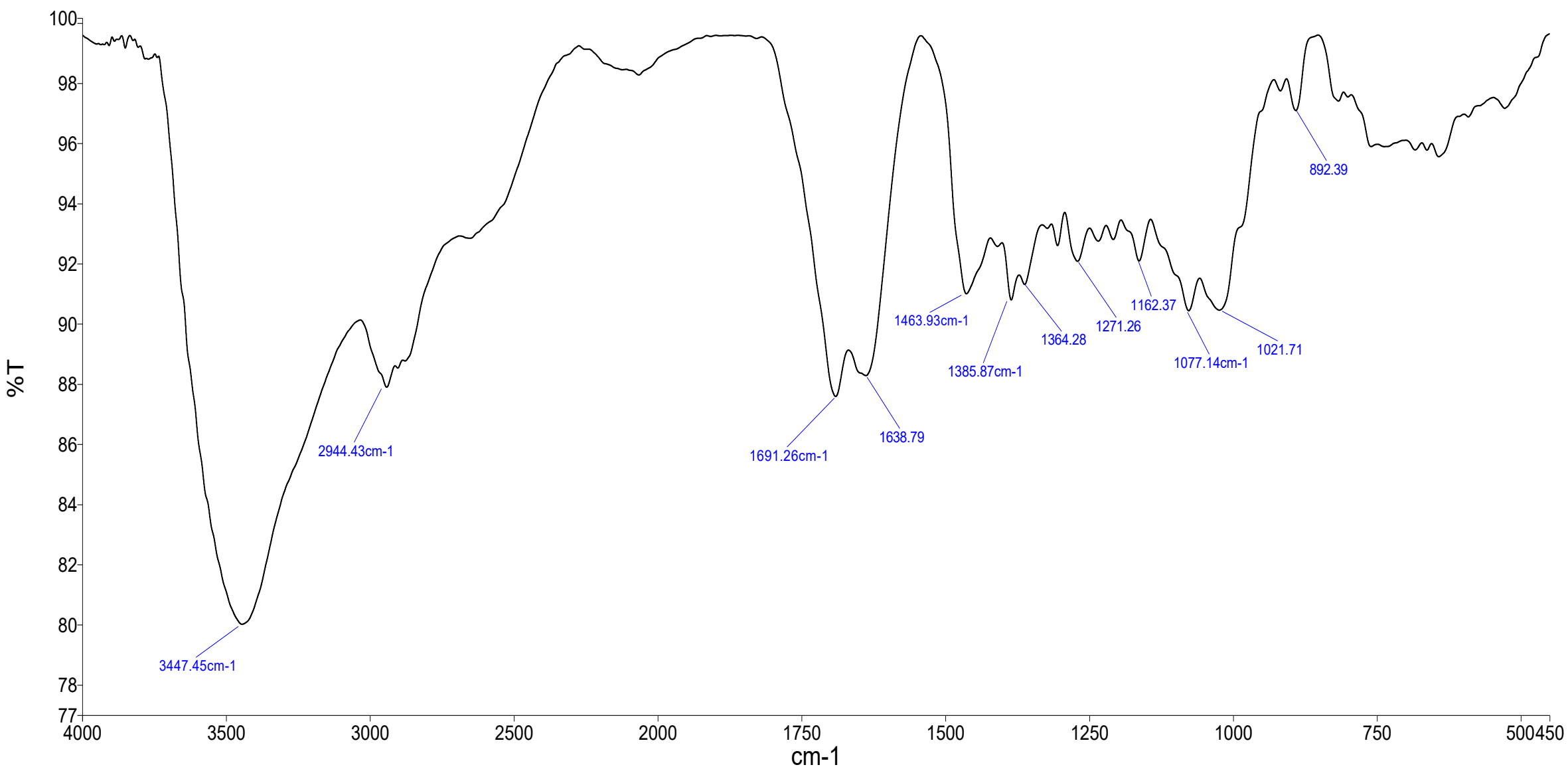

Figure S12. IR spectrum of compound **2**

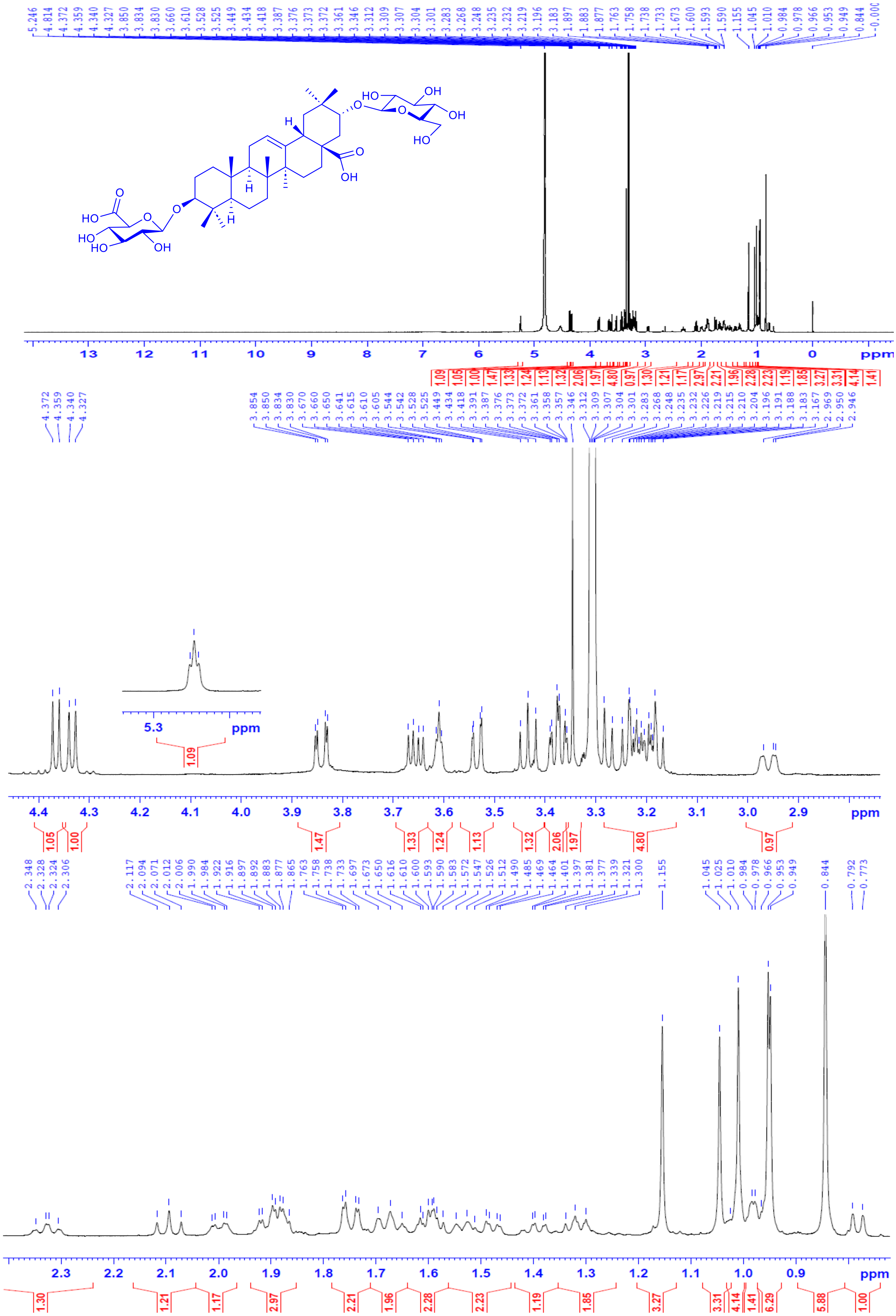

Figure S13. ^{1}H-NMR spectra of compound **2**

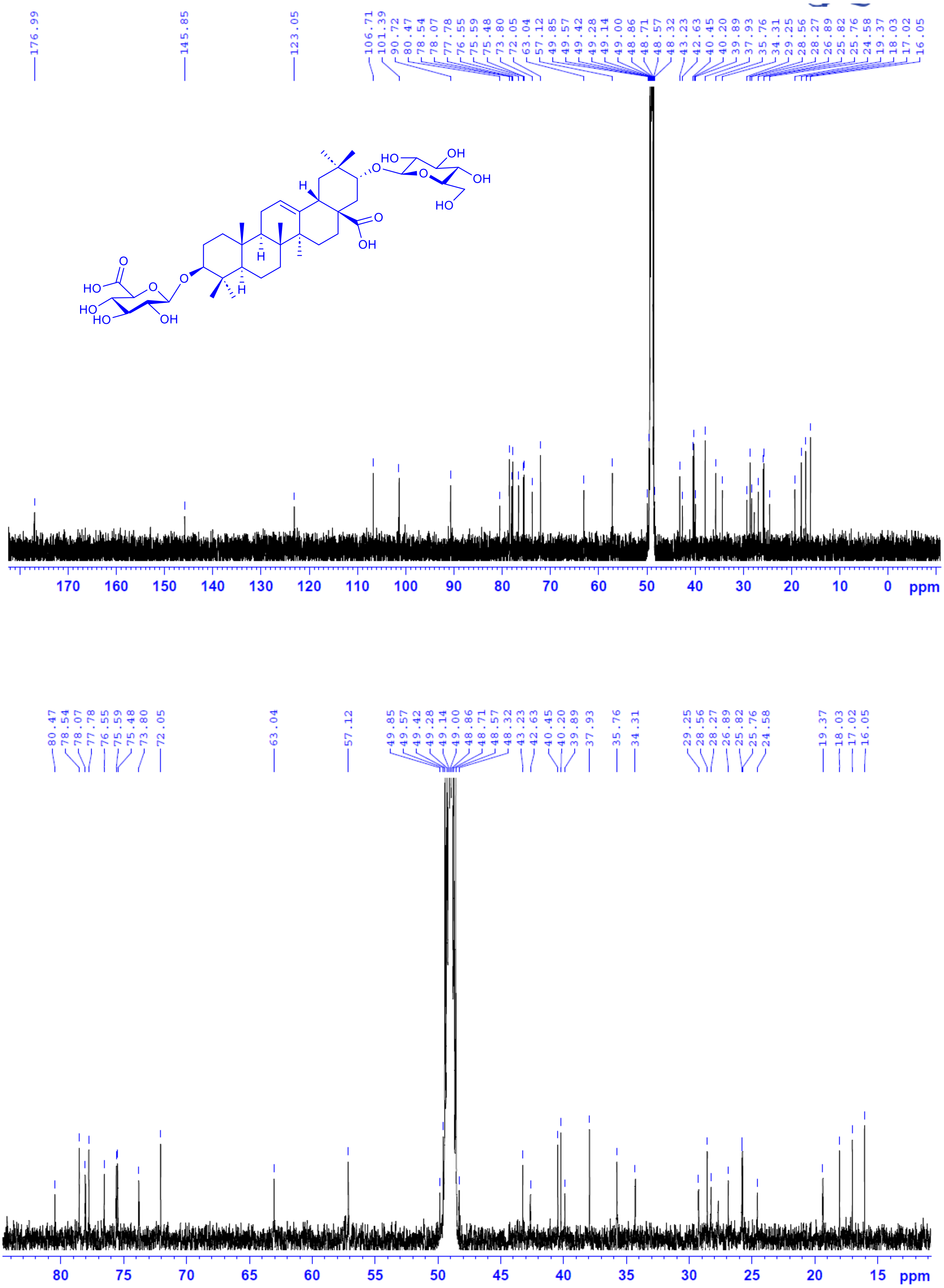

Figure S14. ^{13}C-NMR spectra of compound **2**

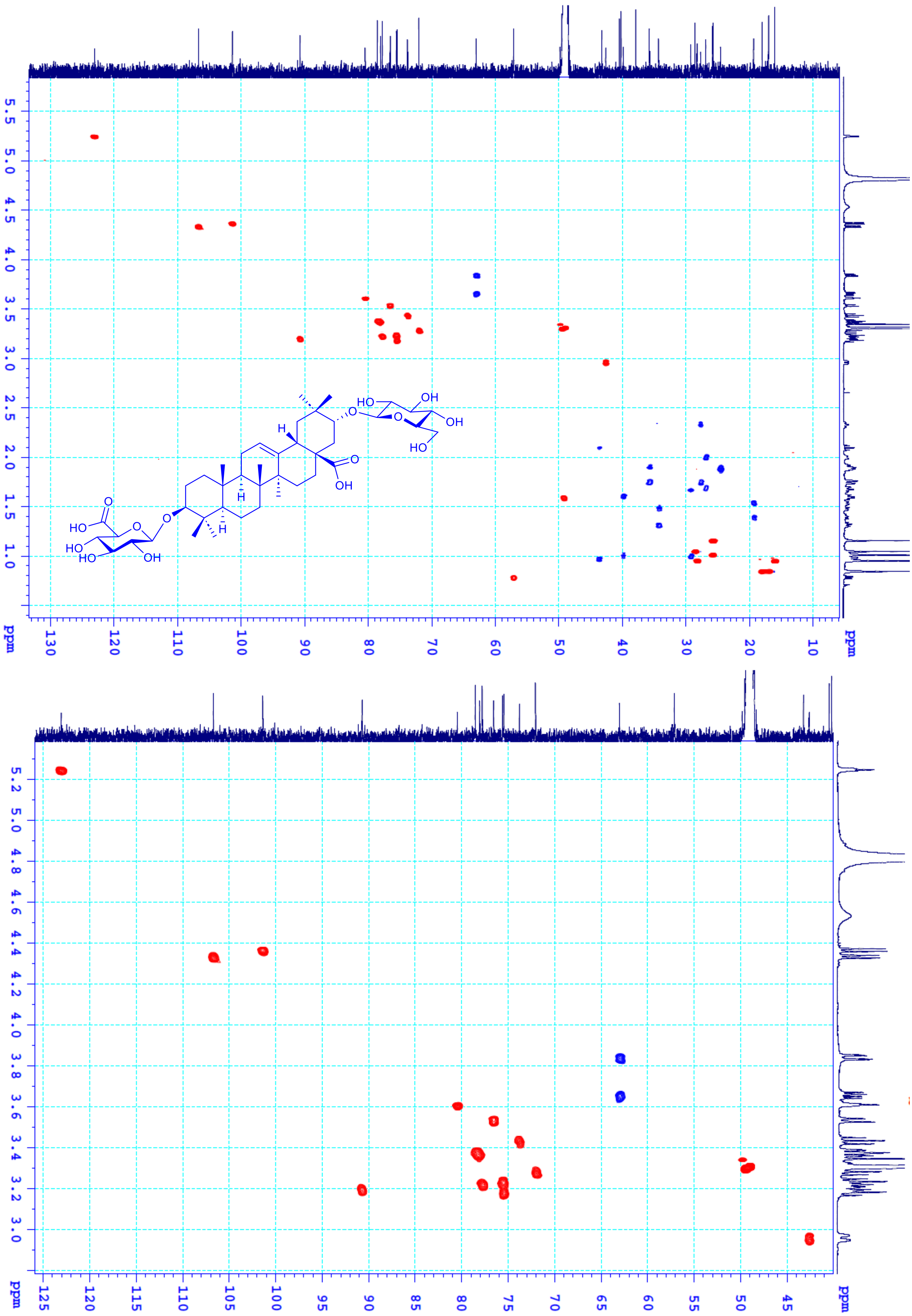

Figure S15. HSQC spectra of compound **2**

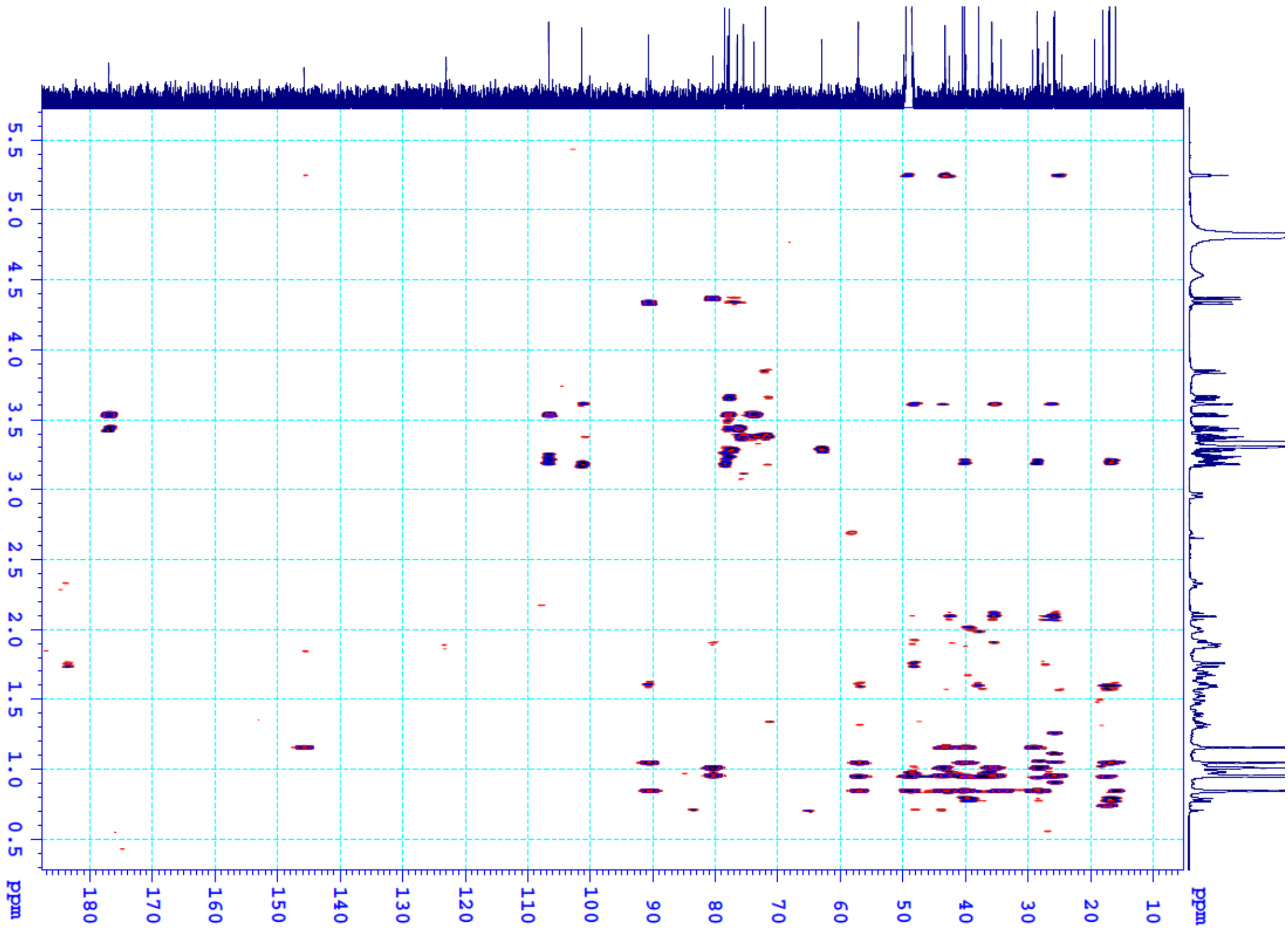

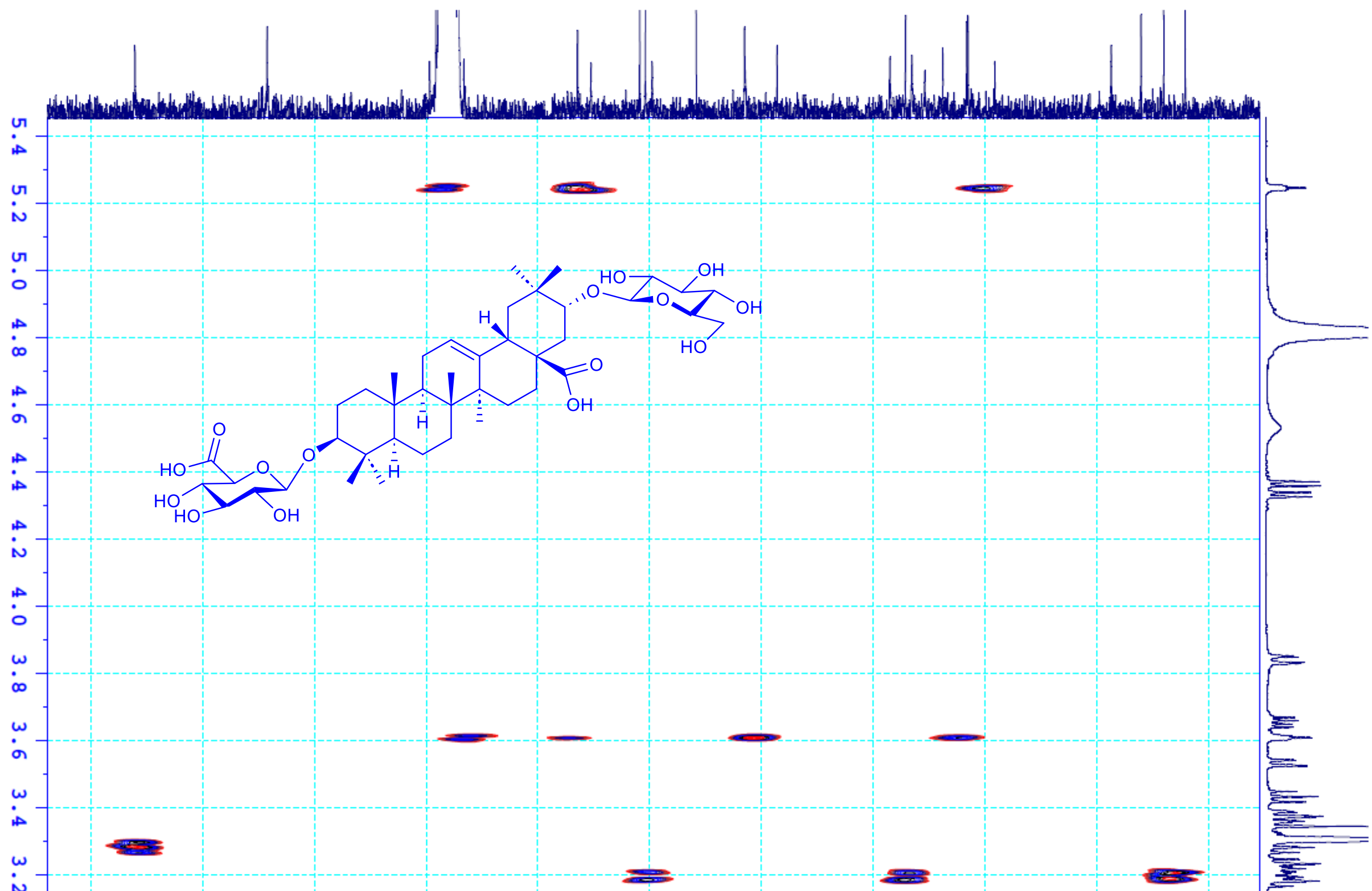

Figure S16. HMBC spectra of compound **2**

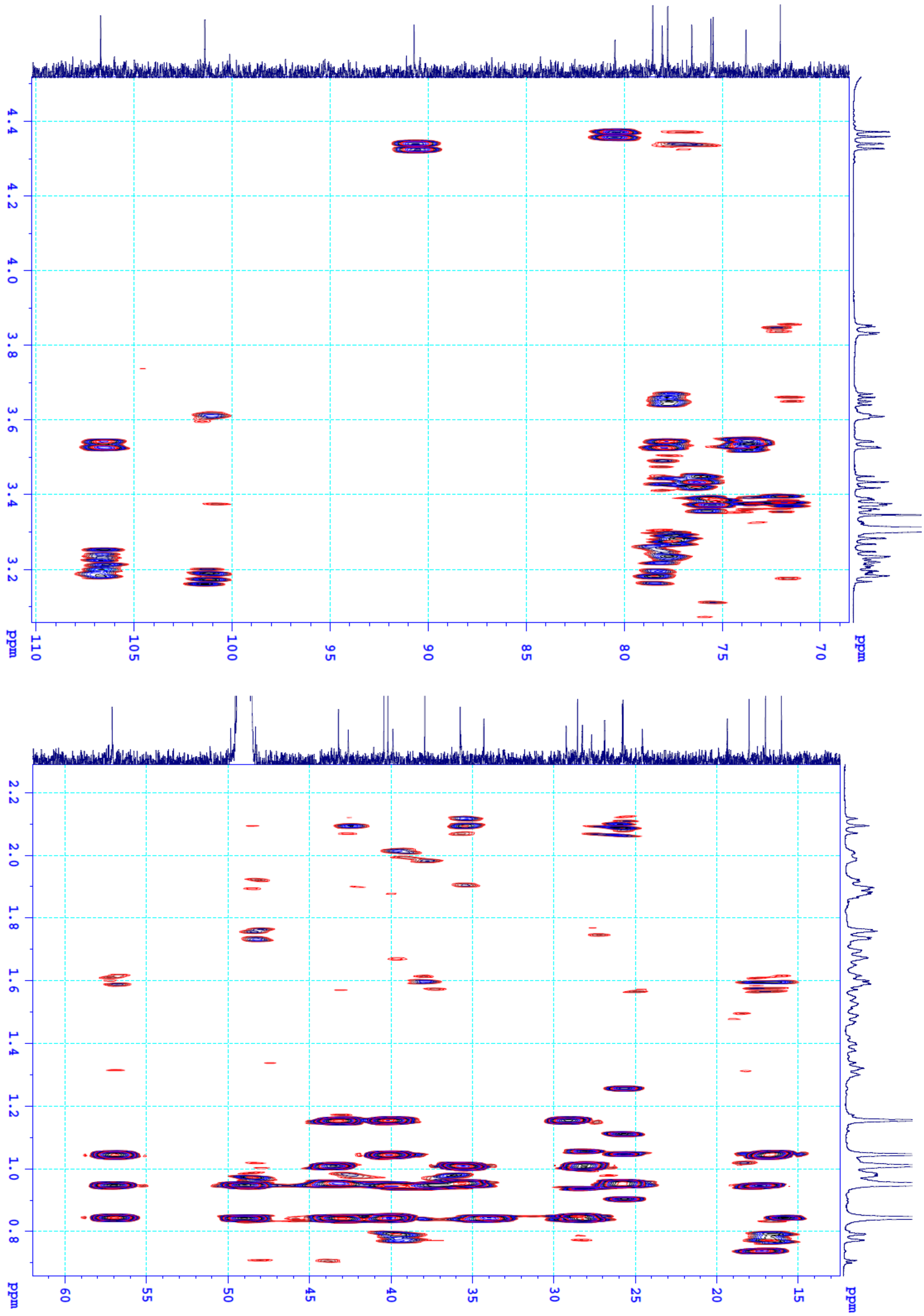

Figure S17. Expanded HMBC spectra of compound **2**

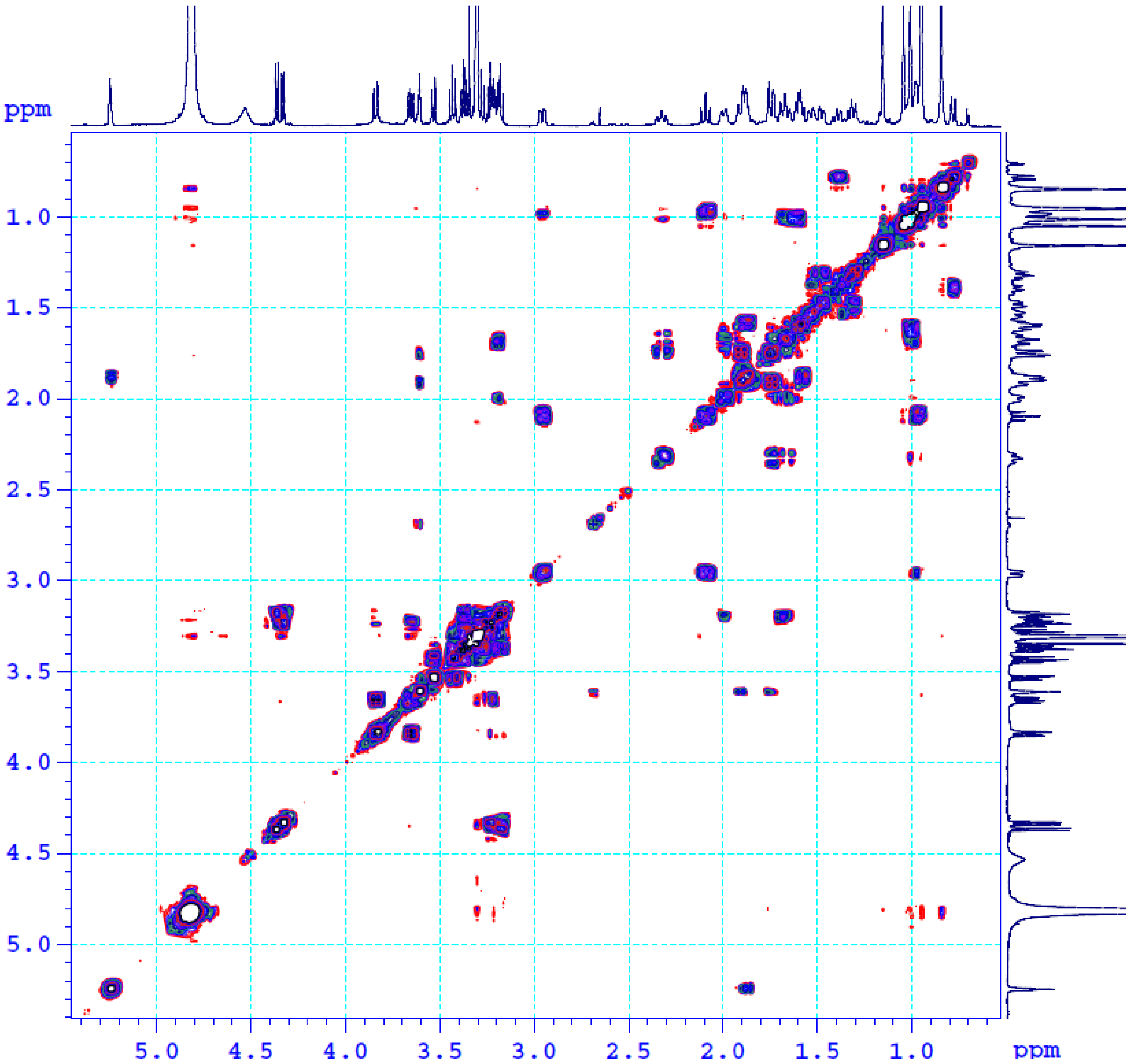

Figure S18. COSY spectrum of compound **2**

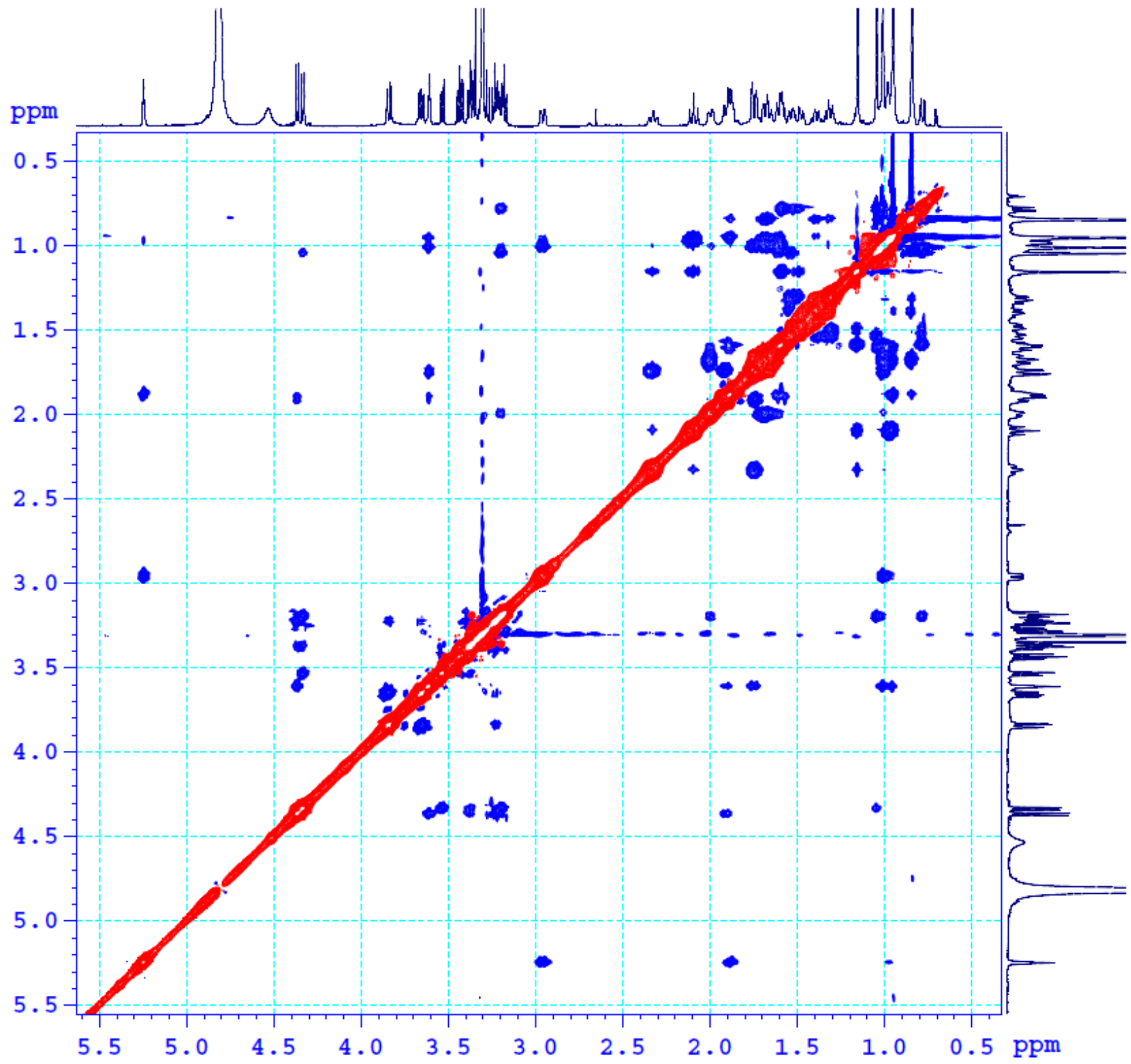

Figure S19. NOESY spectrum of compound **2**

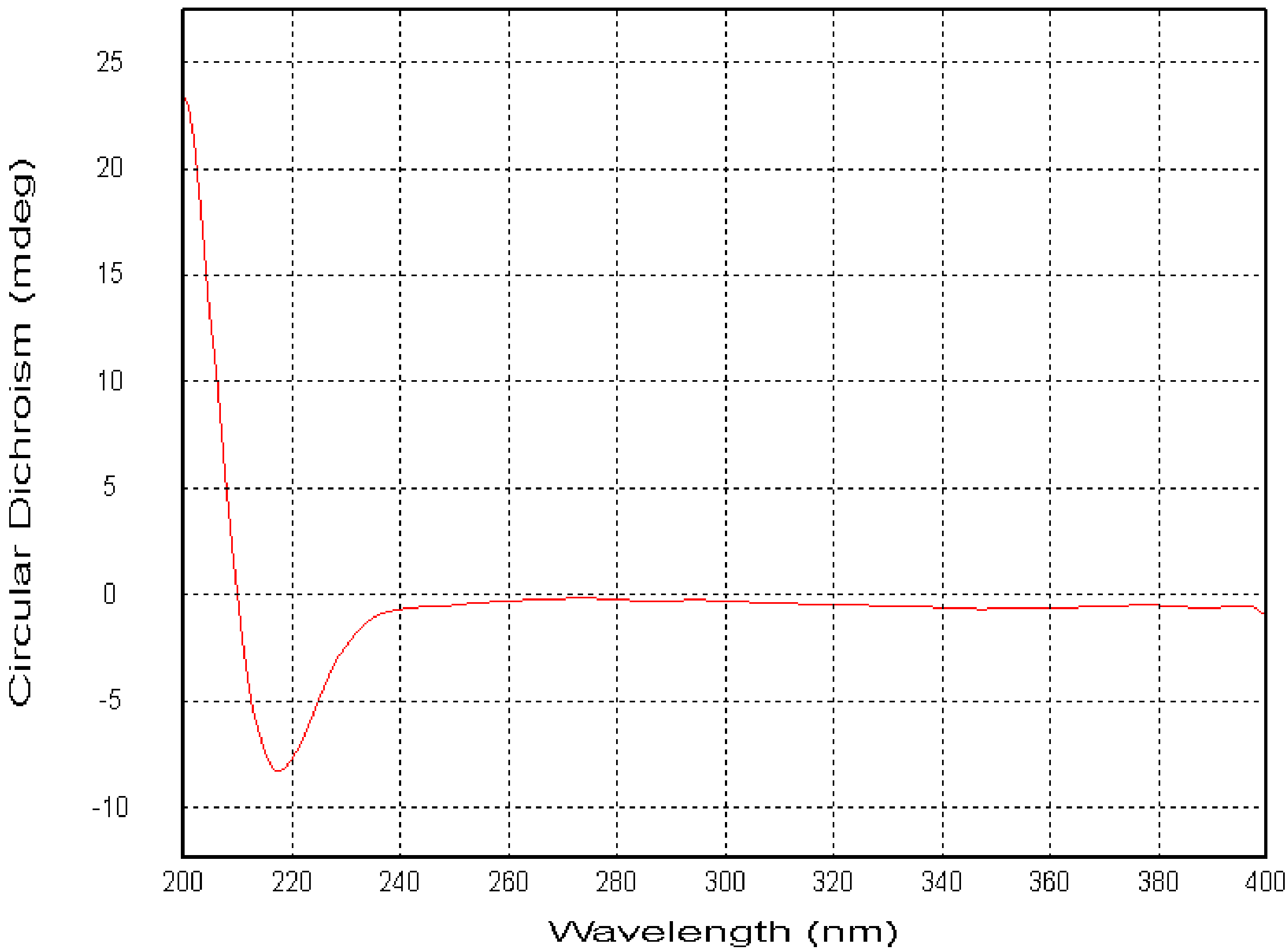

Figure S20. ECD spectrum of compound **2**

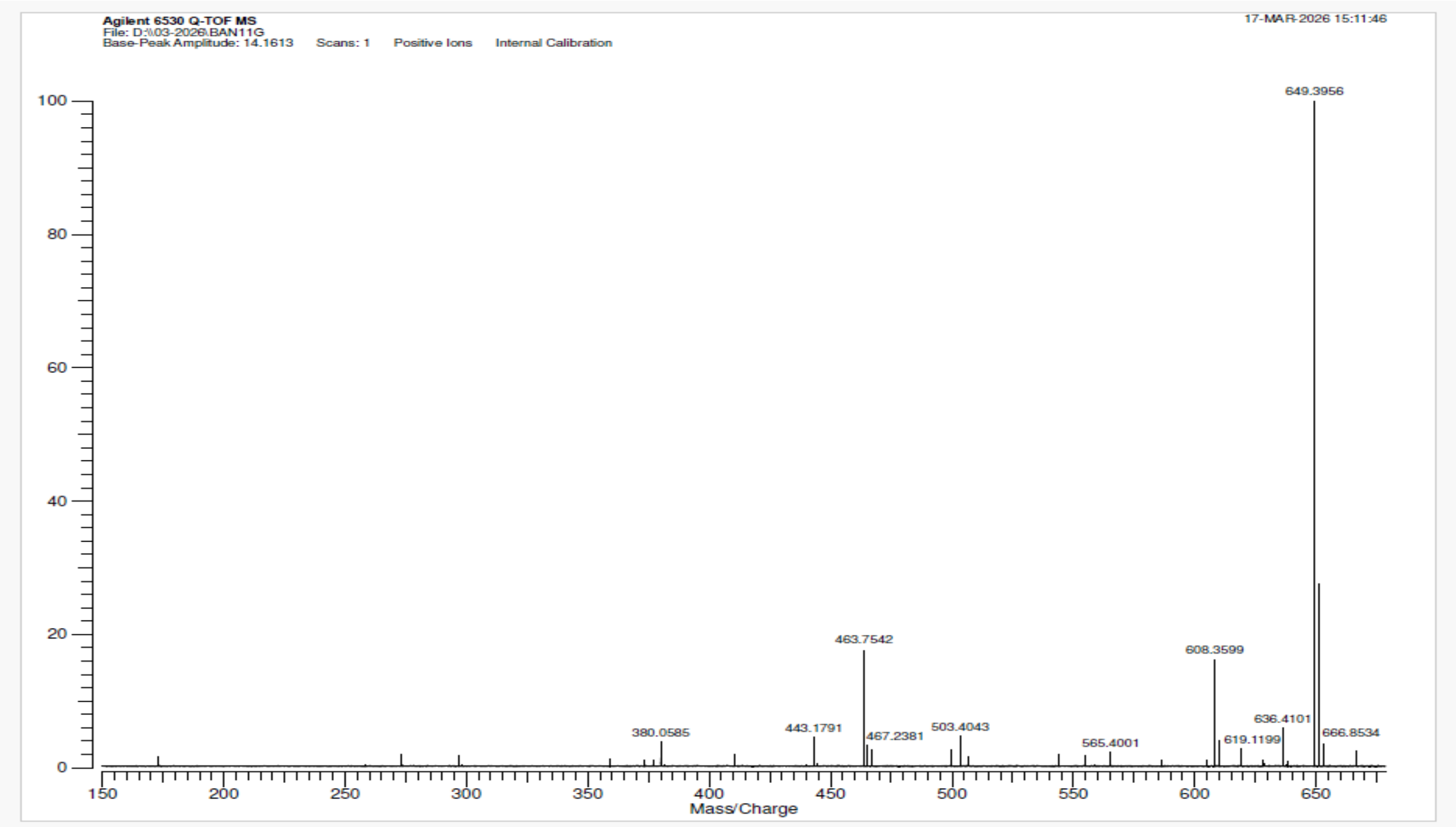

HRESIMS *m/z* 649.3956 $[M+H]^+$, calcd for $[C_{36}H_{57}O_{10}]^+$: 649.3946, Δ = +1.5 ppm

Figure S21. HRESIMS spectrum of compound **3**

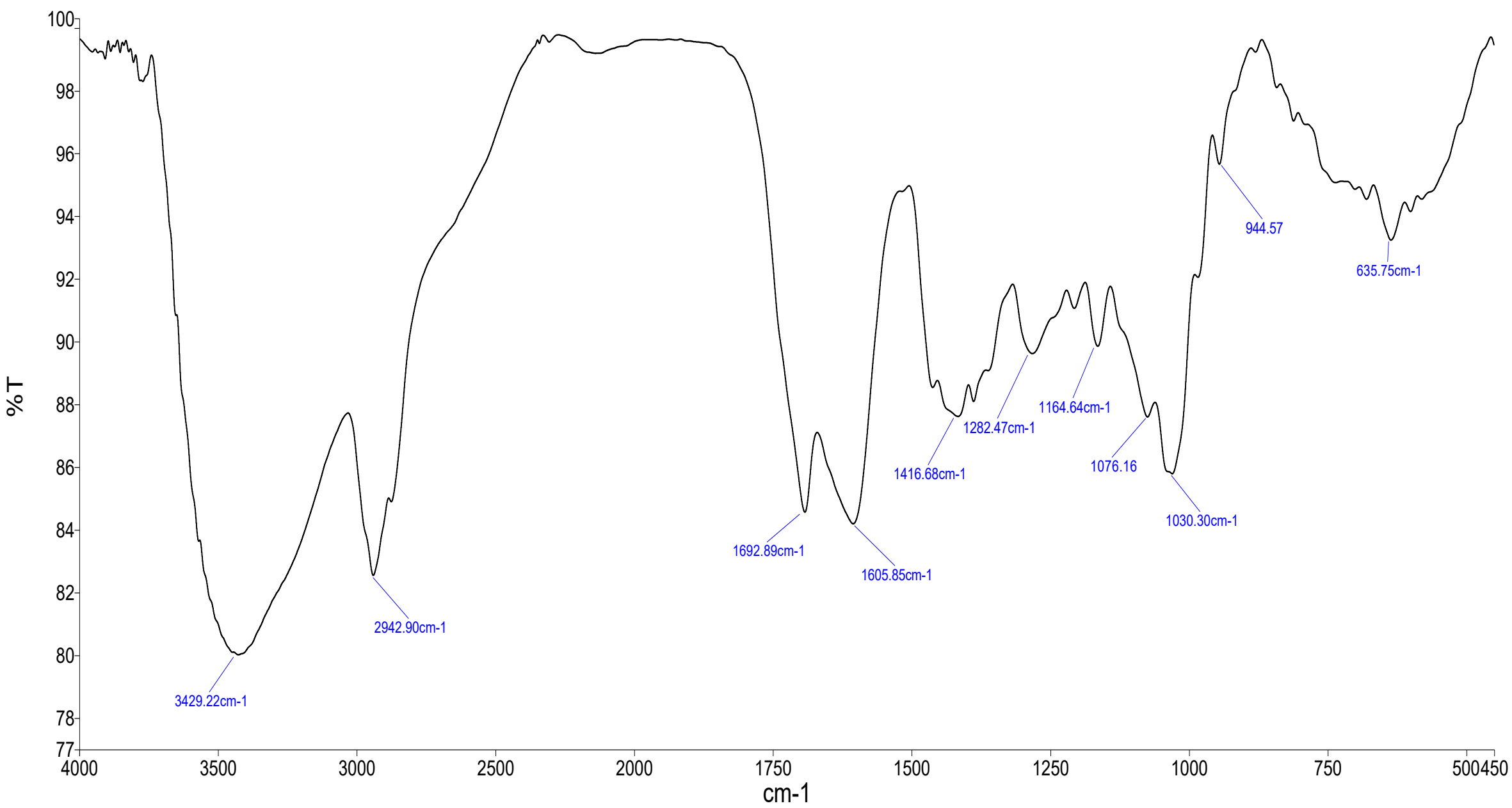

Figure S22. IR spectrum of compound **3**

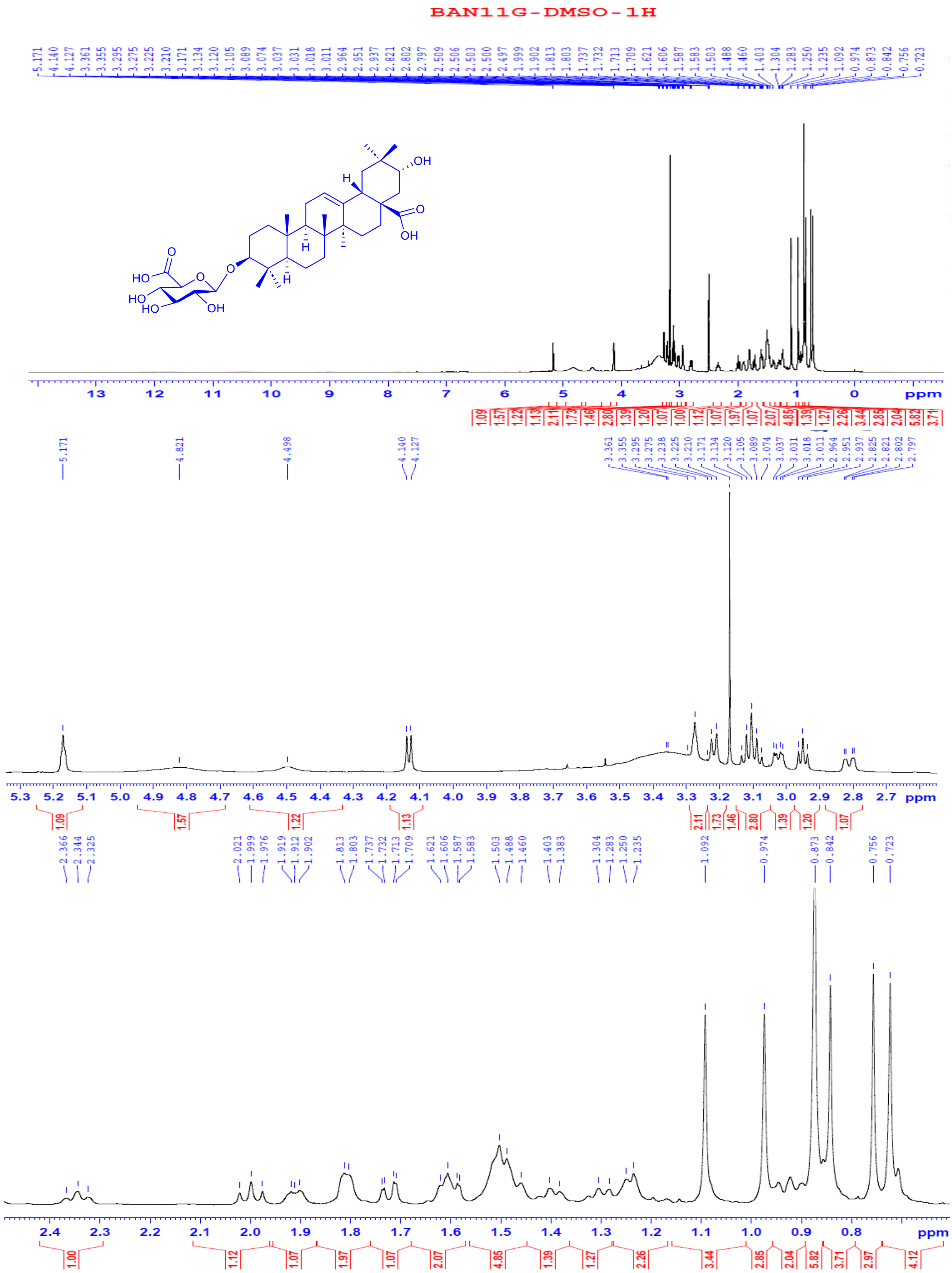

(peaks δ_H 3.17: MeOH in DMSO-d$_6$, Organometallics 2010, 29, 2176–2179. DOI: 10.1021/om100106e)

Figure S23. ^{1}H NMR spectra of compound **3** in DMSO-d$_6$

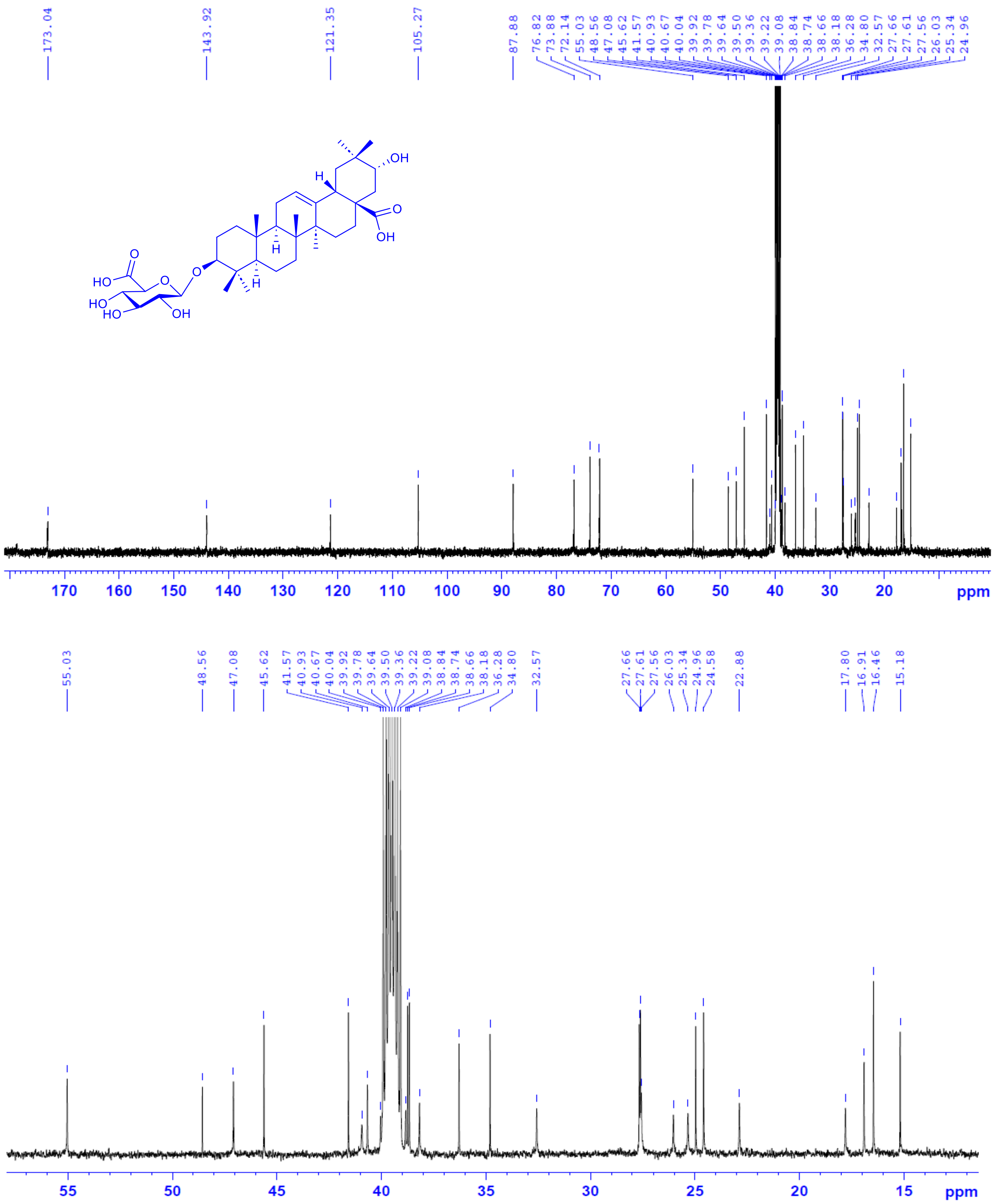

(*peaks δ_C 48.56, MeOH in DMSO-d$_6$, Organometallics 2010, 29, 2176–2179. DOI: 10.1021/om100106e*)

Figure S24. ^{13}C NMR spectra of compound **3** in DMSO-d$_6$

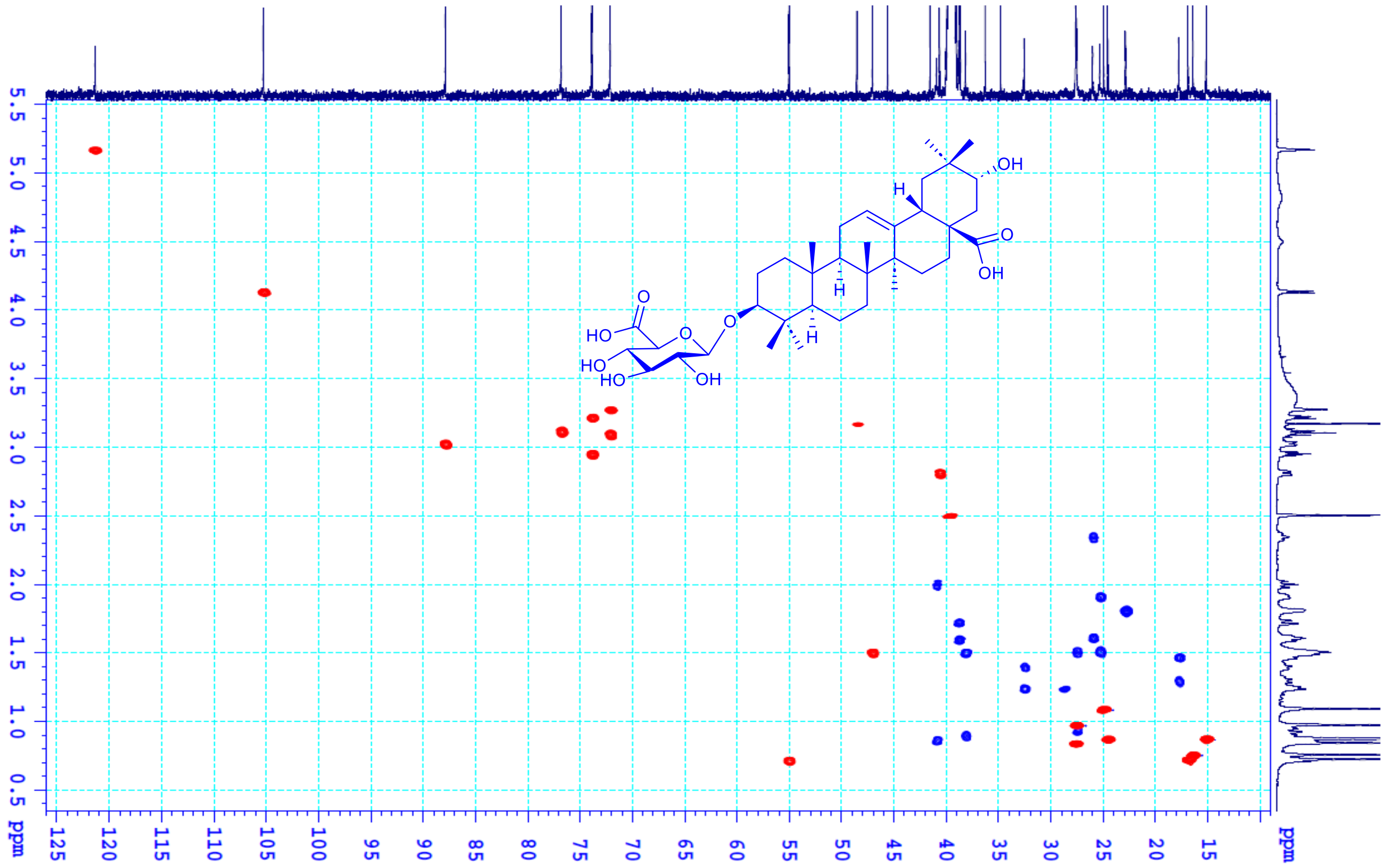

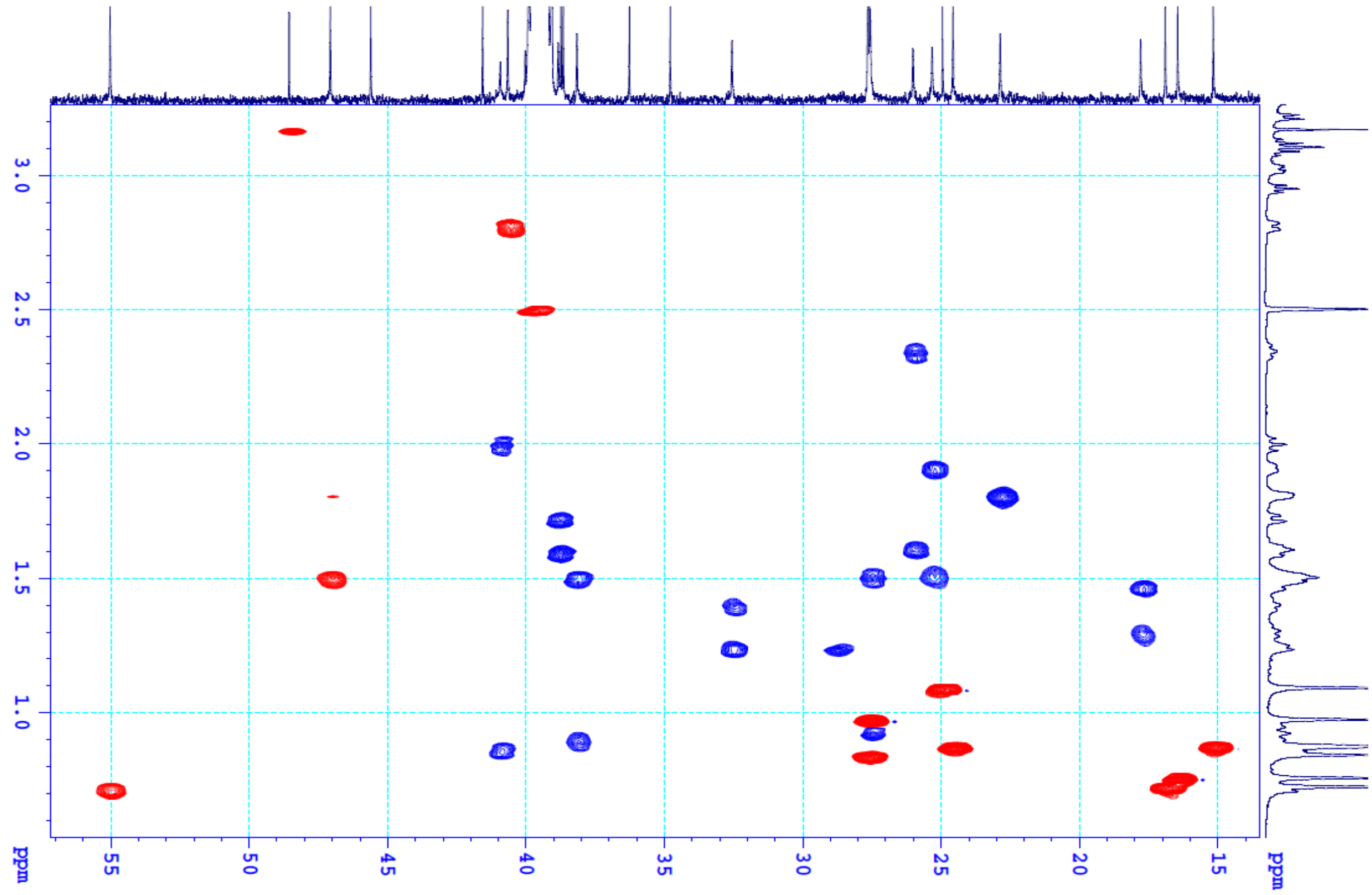

(peaks δ_C 48.56/ δ_H 3.17: MeOH in DMSO-d_6, Organometallics 2010, 29, 2176−2179. DOI: 10.1021/om100106e)

Figure S25. HSQC spectra of compound **3** in DMSO-d_6

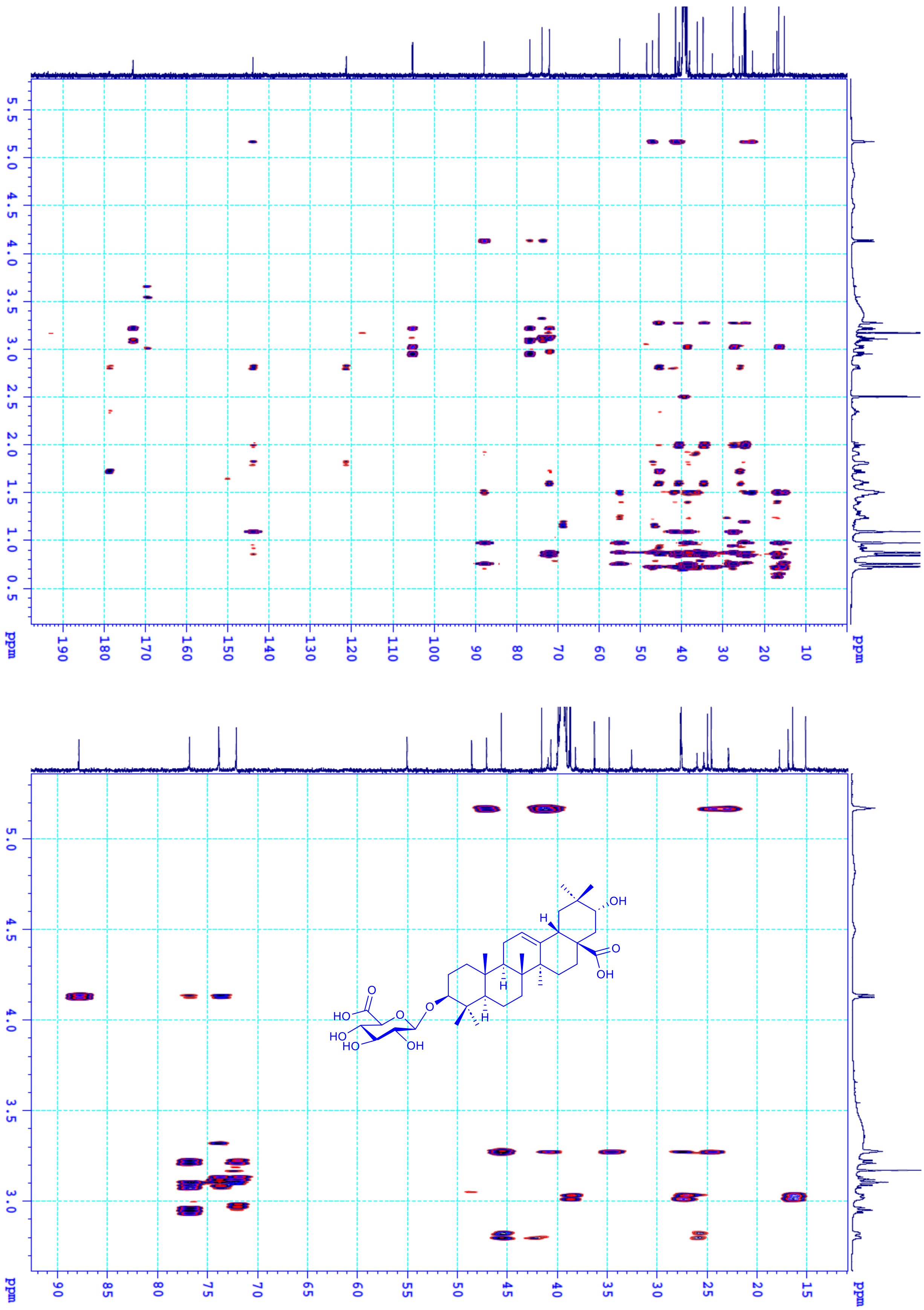

Figure S26. HMBC spectra of compound **3** in DMSO-d_6

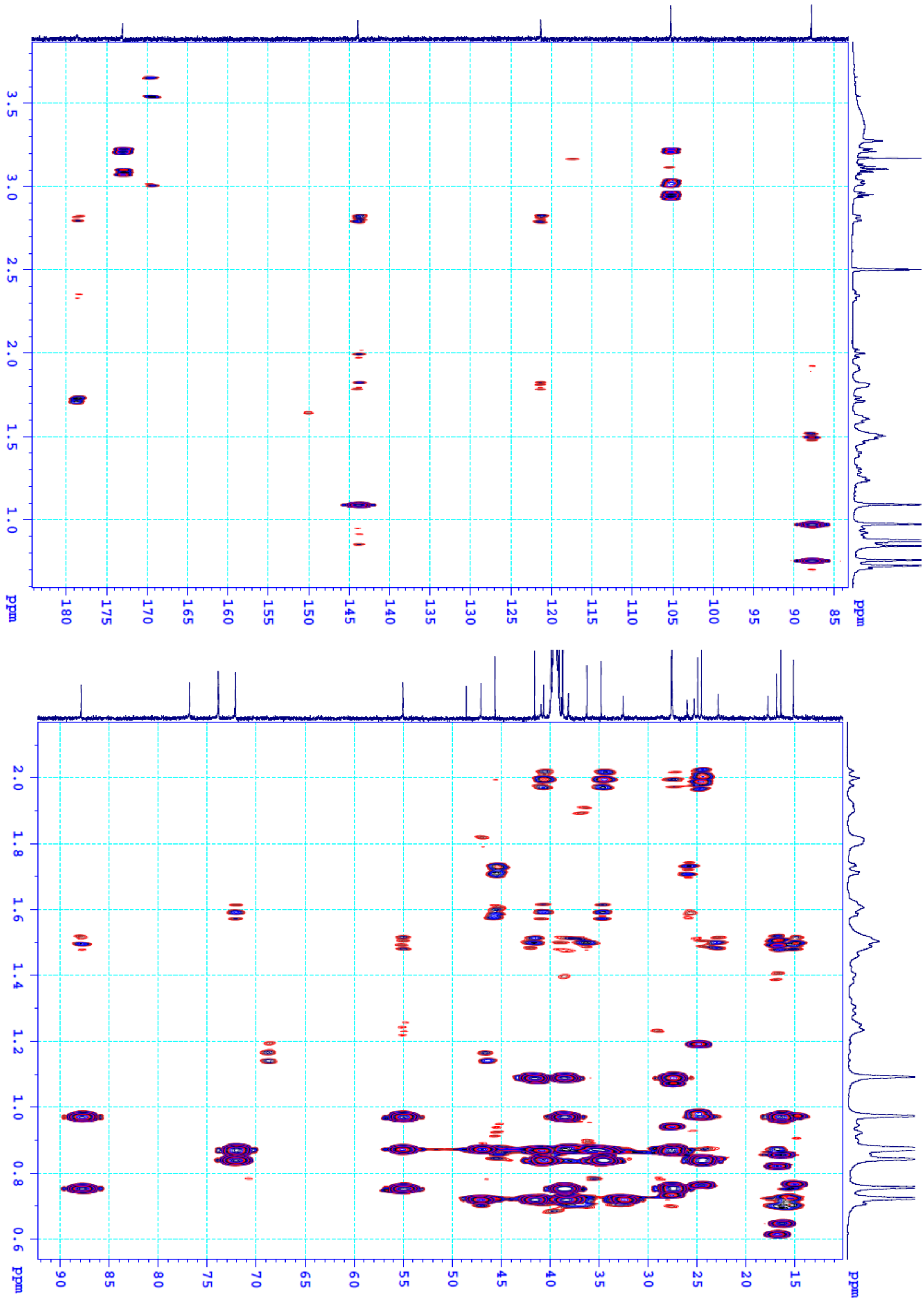

Figure S27. Expanded HMBC spectra of compound **3** in DMSO-d_6

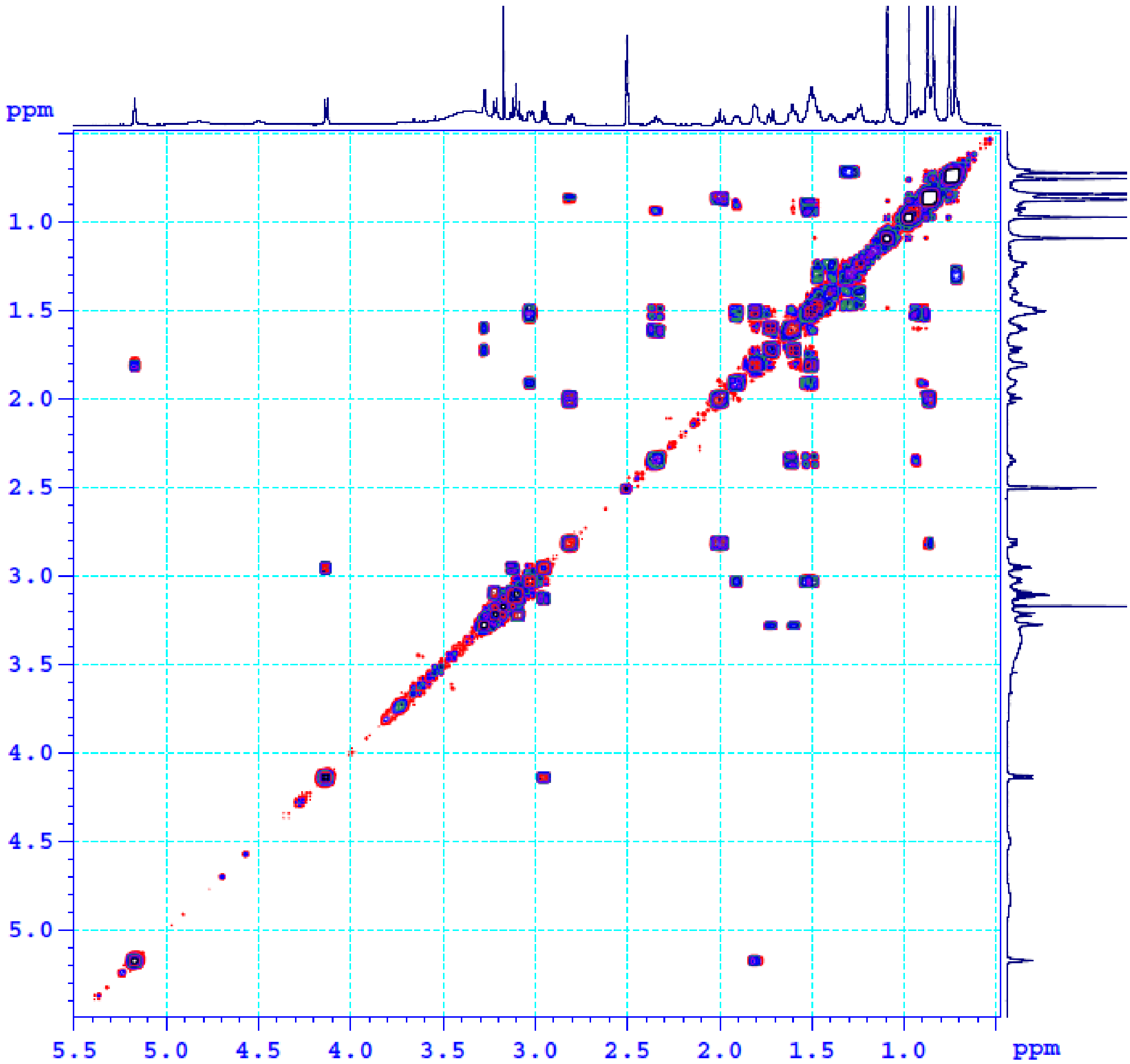

Figure S28. COSY spectrum of compound **3** in DMSO-d_6

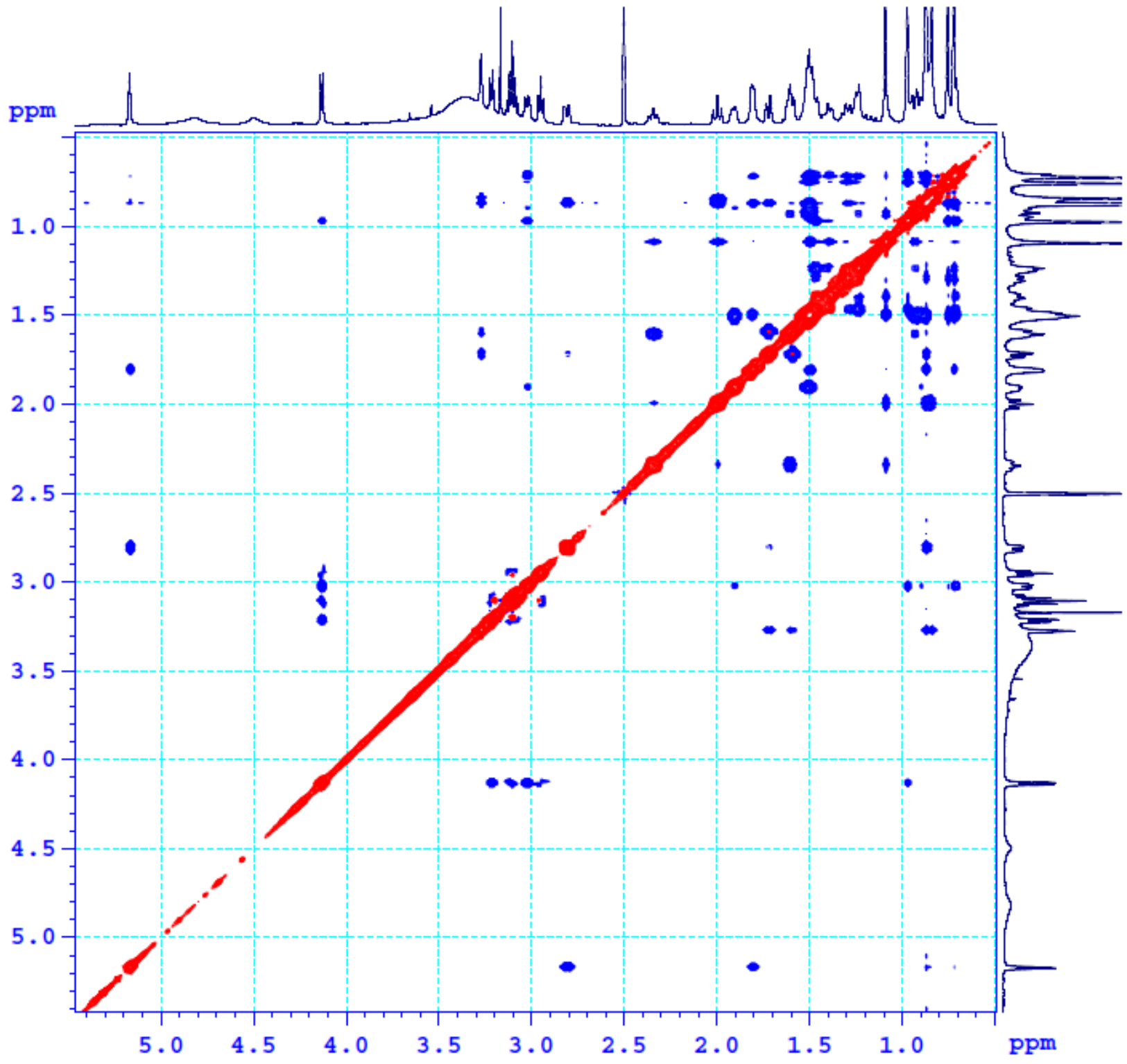

Figure S29. NOESY spectrum of compound **3** in DMSO-d_6

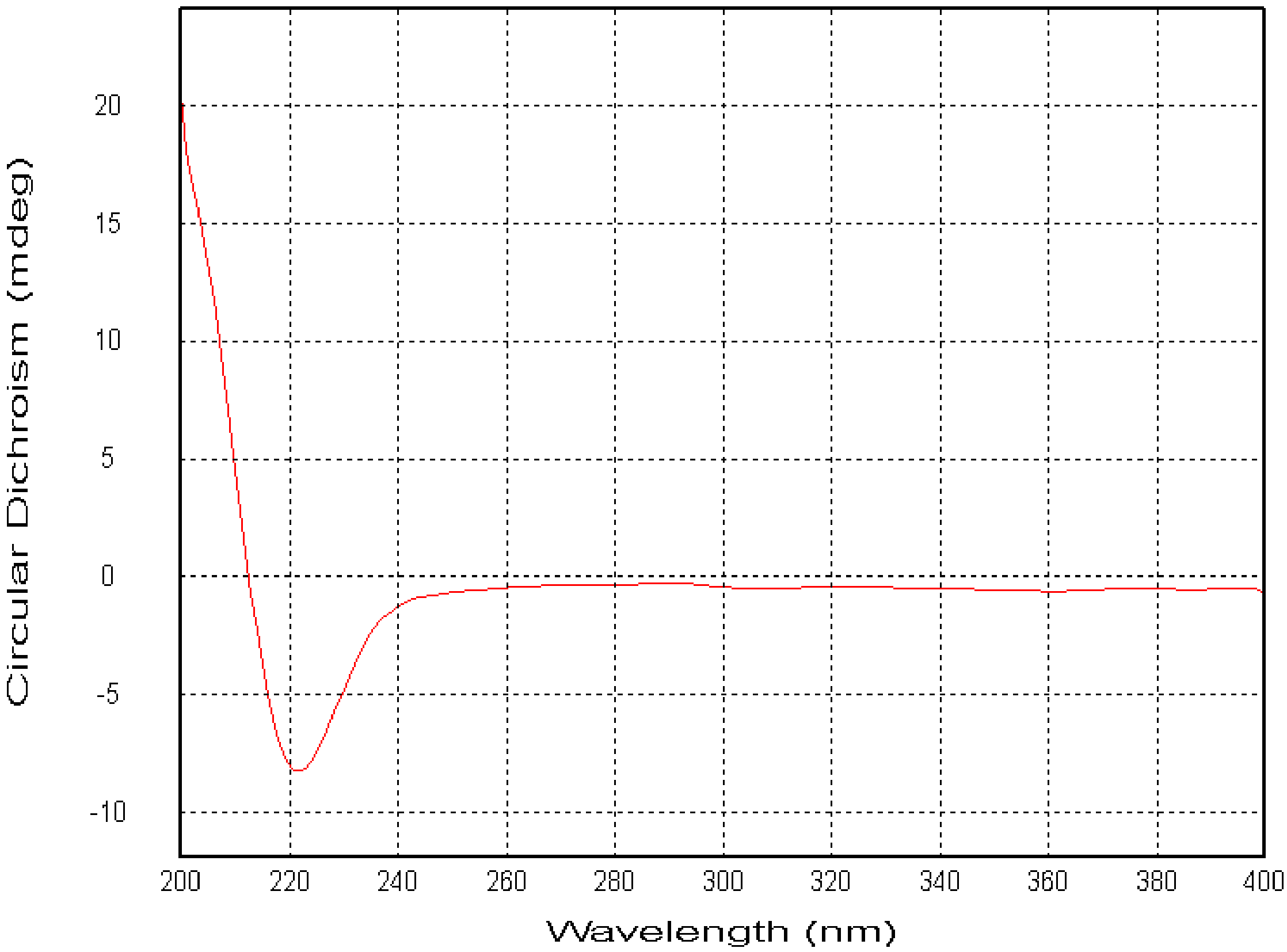

Figure S30. ECD spectrum of compound **3**

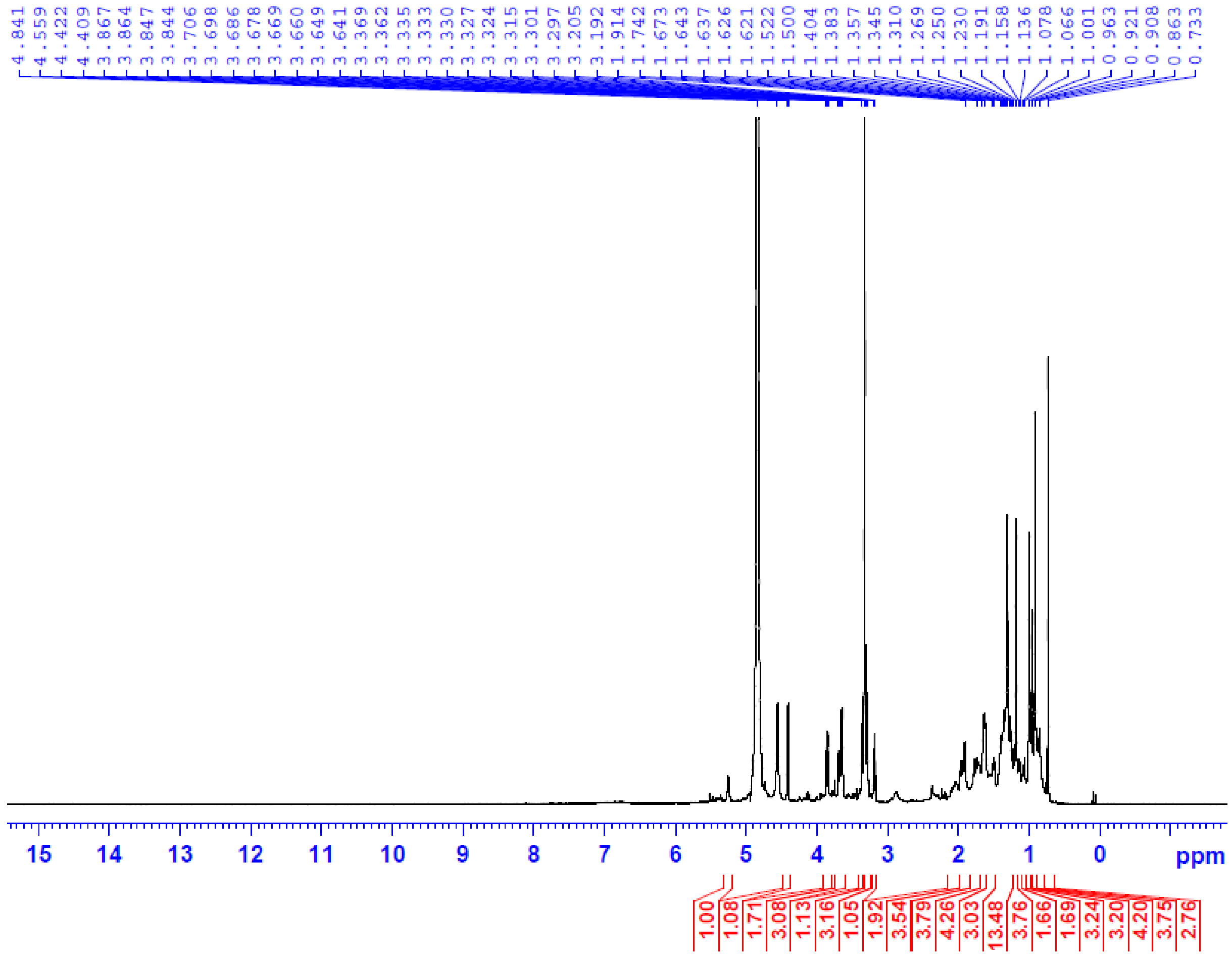

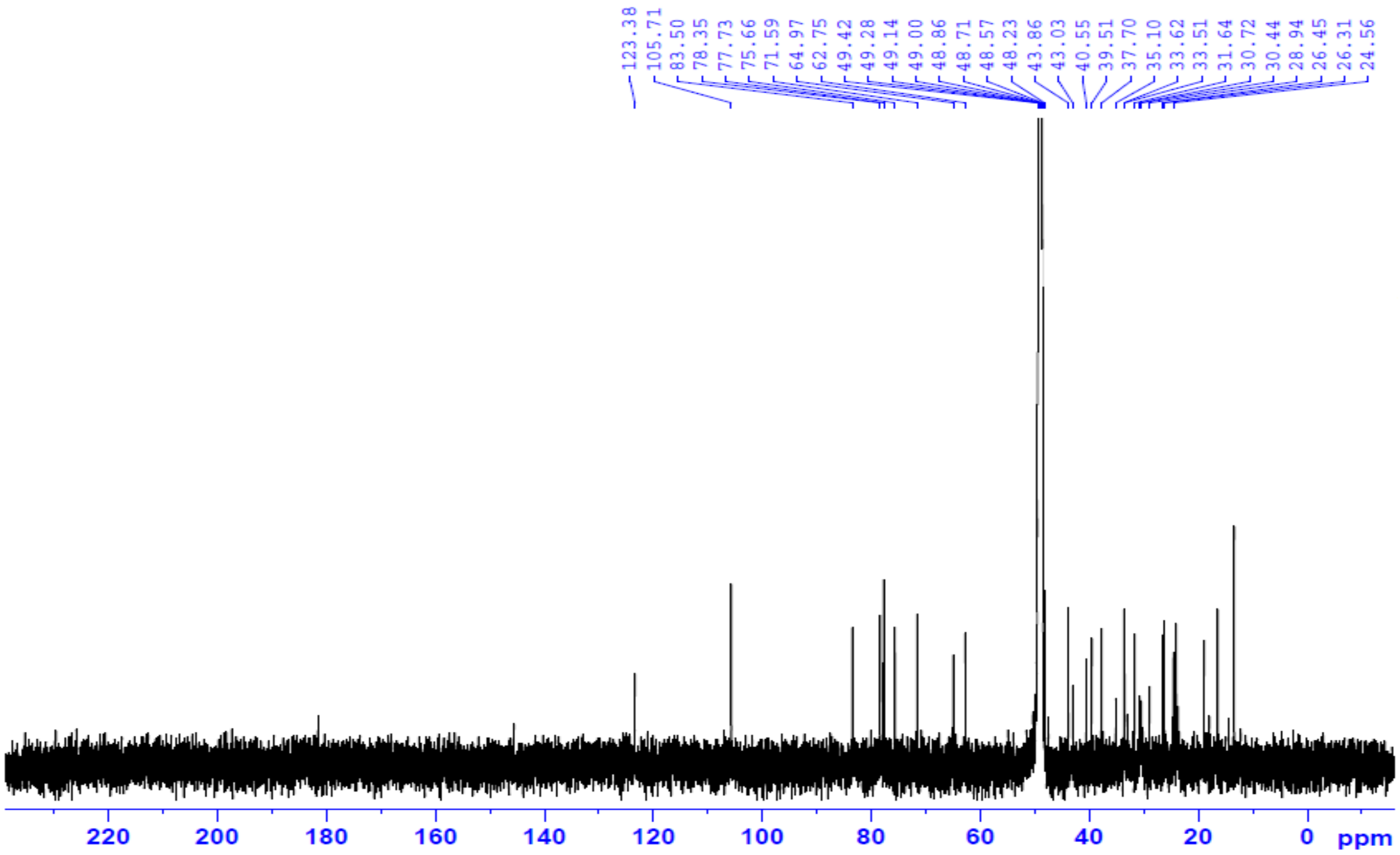

Figure S31. ^{1}H and ^{13}C NMR spectra for compound **4** in CD_3OD.

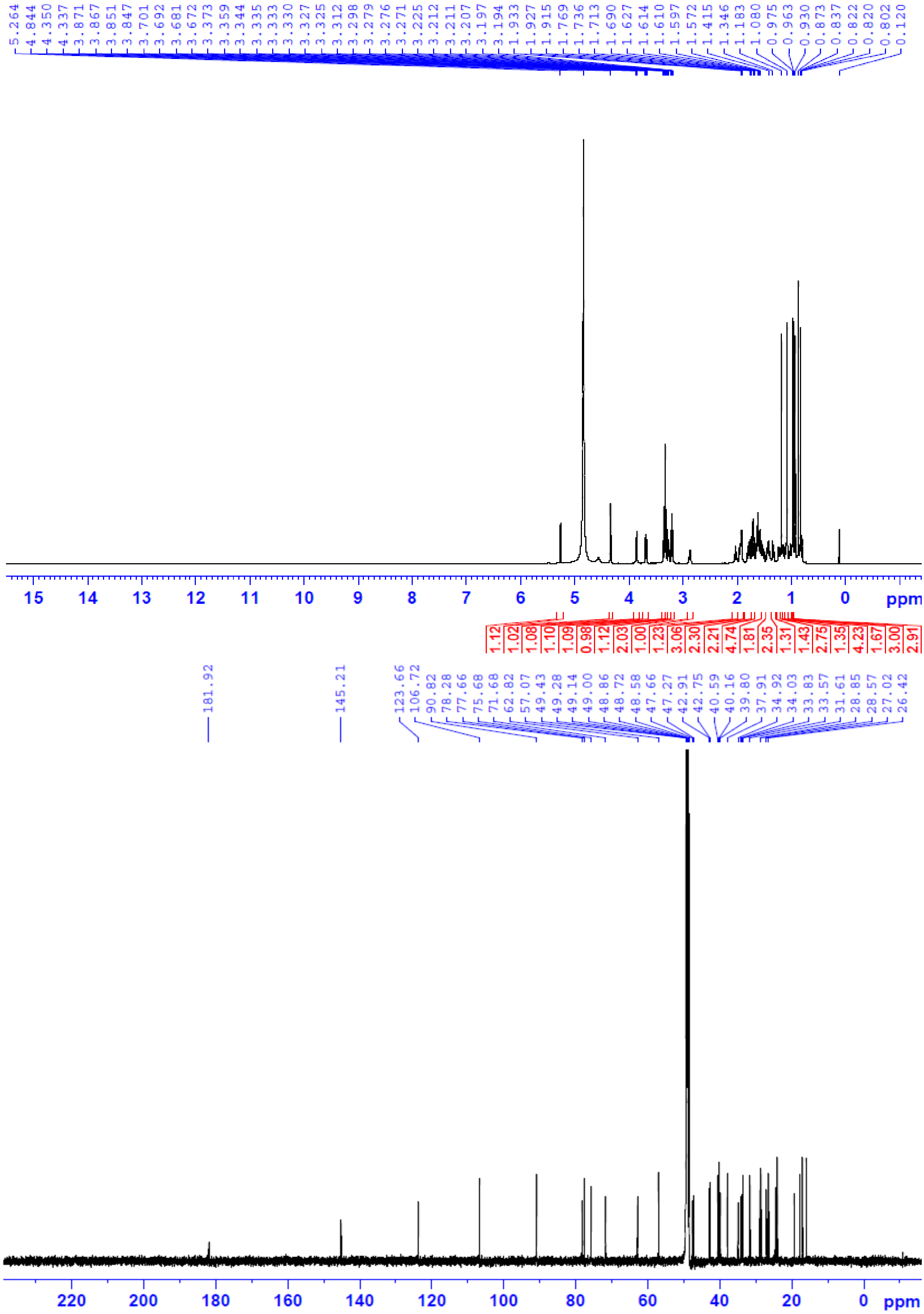

Figure S32. ^{1}H and ^{13}C NMR spectra for compound **5** in CD_3OD.

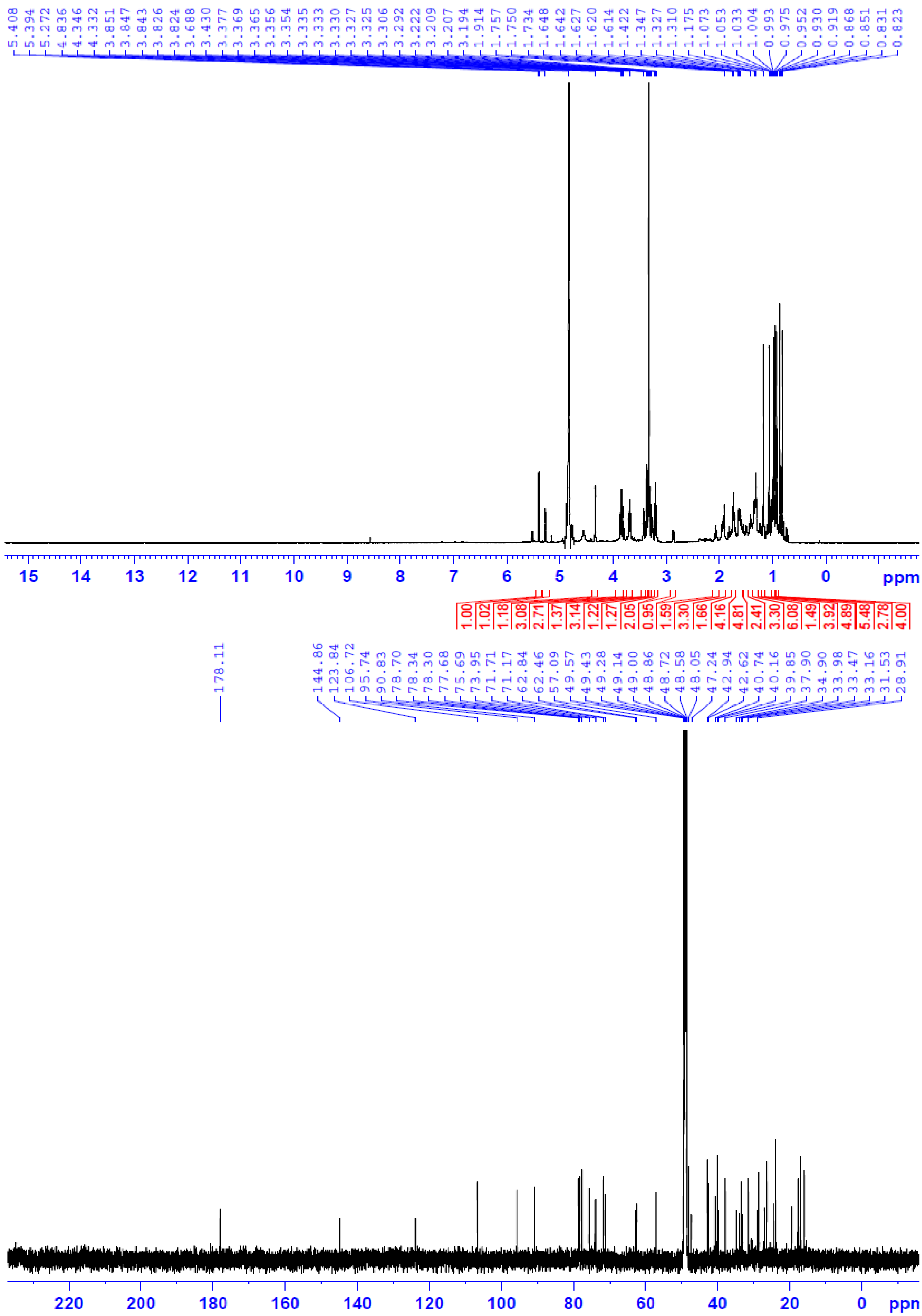

Figure S33. ^{1}H and ^{13}C NMR spectra for compound **6** in CD_3OD.

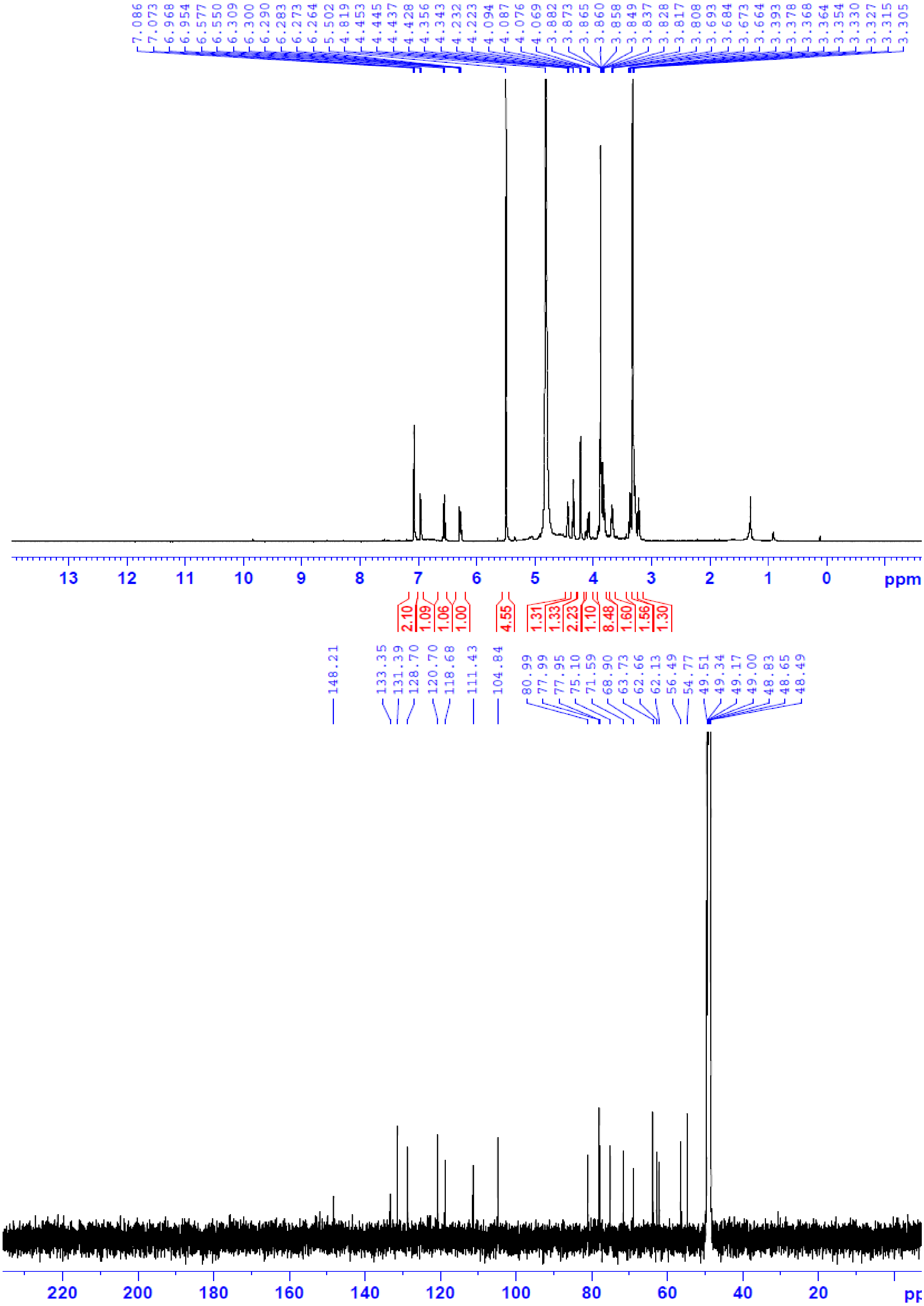

Figure S34. ^{1}H and ^{13}C NMR spectra for compound **7** in CD_3OD.

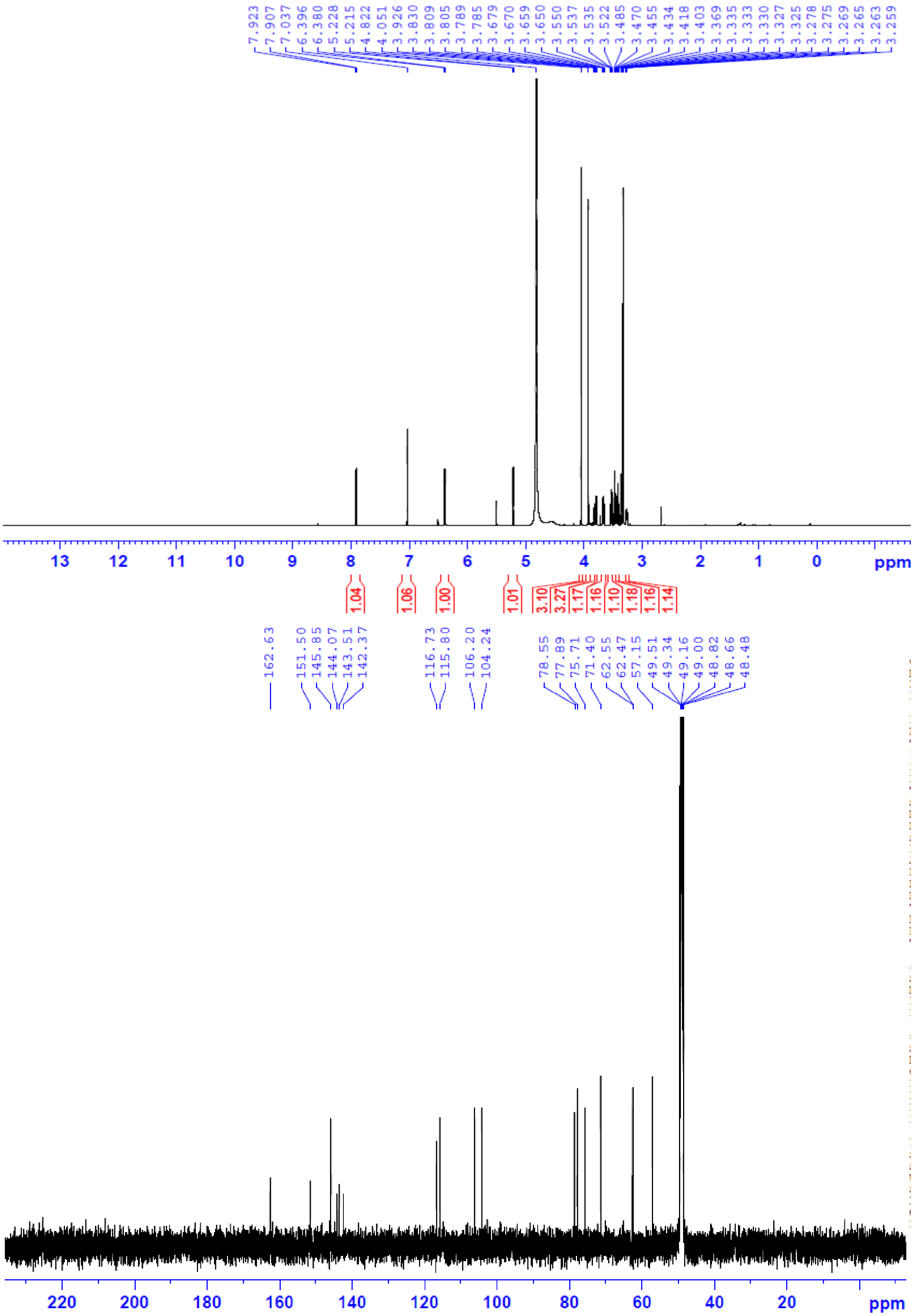

Figure S35. ^{1}H and ^{13}C NMR spectra for compound **8** in CD_3OD.

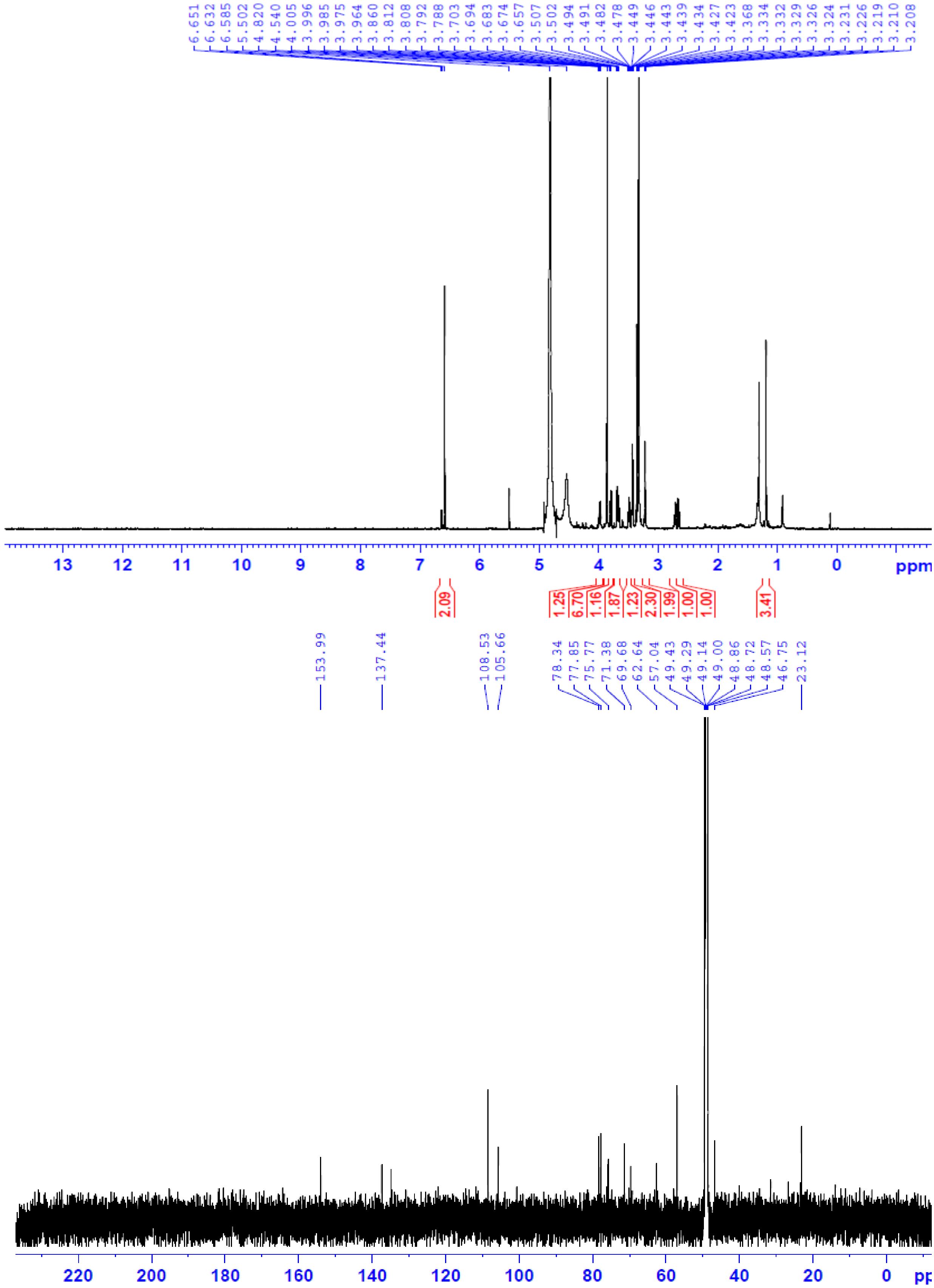

Figure S36. ^{1}H and ^{13}C NMR spectra for compound **9** in CD_3OD.

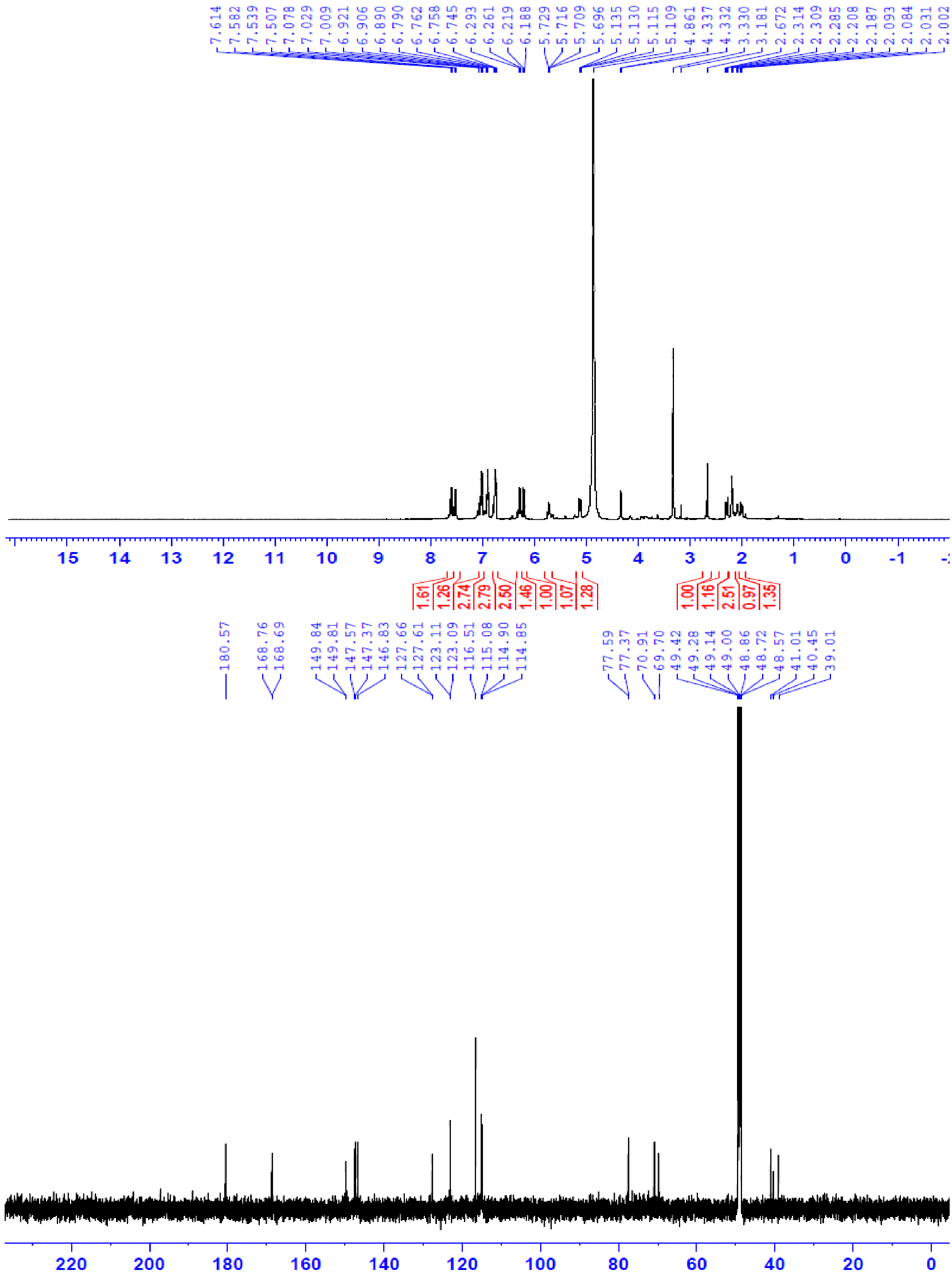

Figure S37. ^{1}H and ^{13}C NMR spectra for compound **10** in CD_3OD.

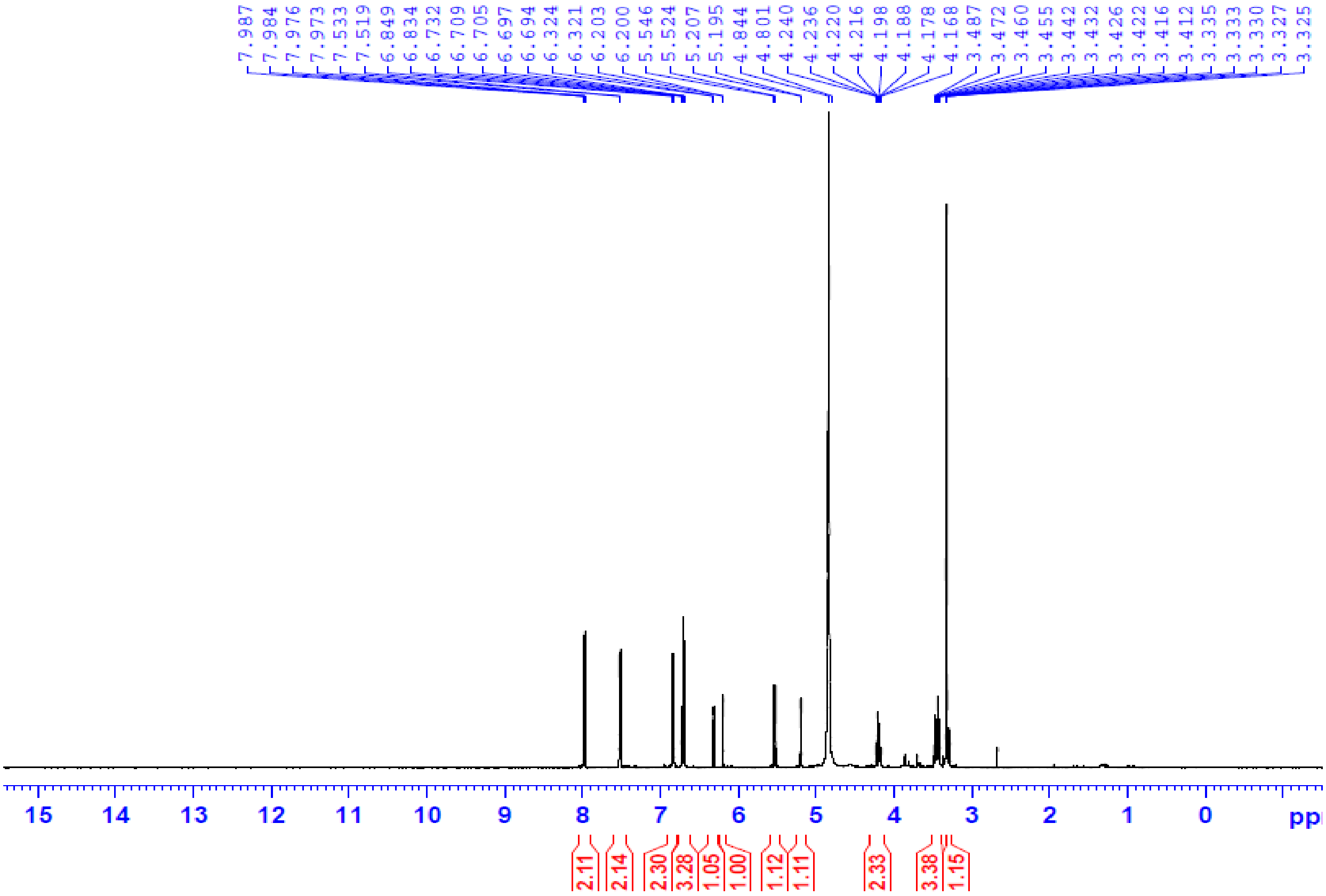

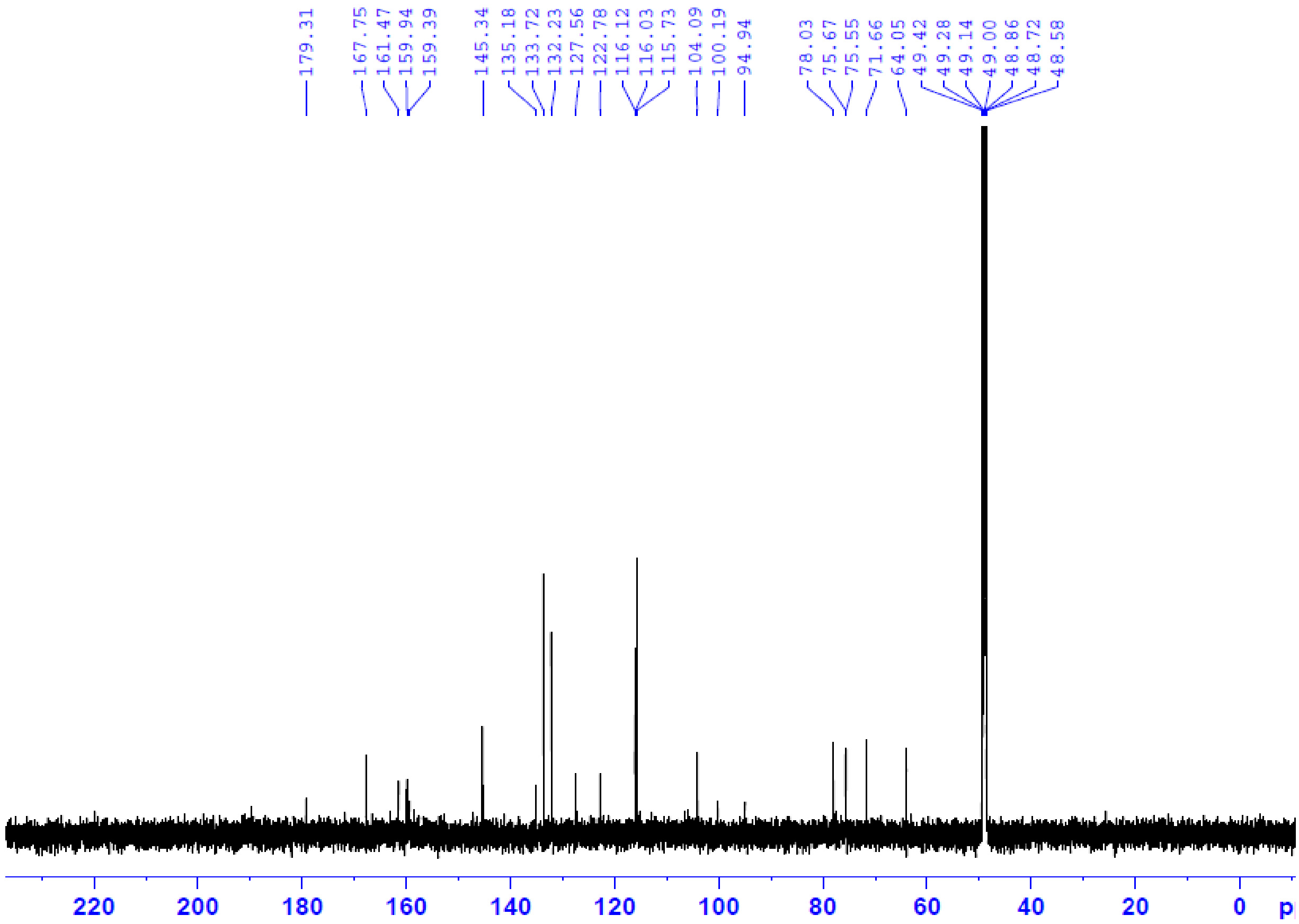

Figure S38. ^{1}H and ^{13}C NMR spectra for compound **11** in CD_3OD.

Table S2. ^{1}H and ^{13}C NMR spectroscopic data for compound **4** in CD_3OD.

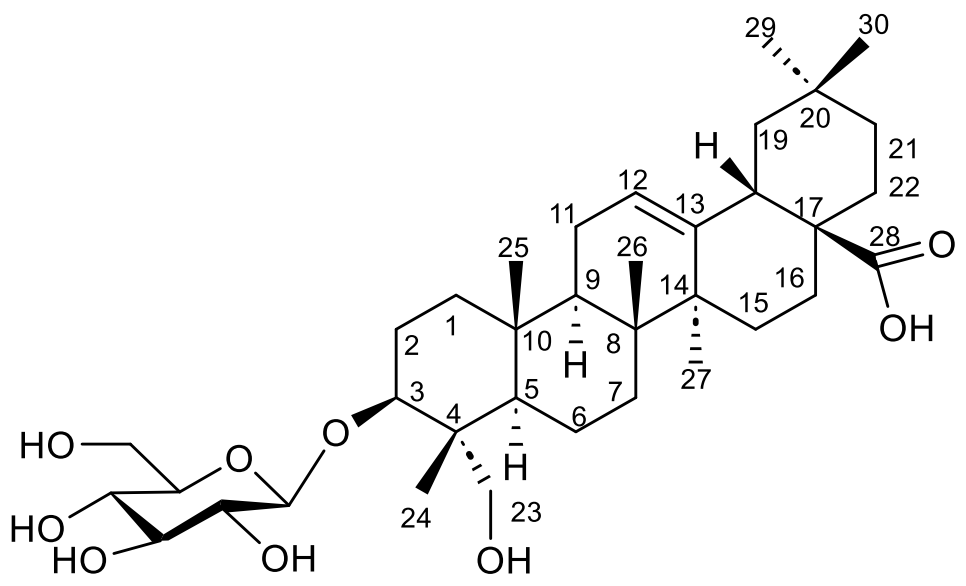

HN-saponin D1

Pos.	$^{a}\delta_C$	$^{b}\delta_H$ (mult., J in Hz)	Pos.	$^{a}\delta_C$	$^{b}\delta_H$ (mult., J in Hz)
1	39.5	1.00 (m)/1.63 (m)	3-O-glucose		
2	26.3	1.75 (m)/1.97 (m)	1′	105.7	4.42 (d, 7.8)
3	83.5	3.65 (dd, 9.0, 3.0)	2′	75.7	3.19 (dd, 9.0, 7.8)
4	43.9	-	3′	78.4	3.36 (t, 9.0)
5	48.2	1.26 (br d, 11.4)	4′	71.6	3.31 (t, 9.0)
6	18.9	1.40 (m)/1.52 (m)	5′	77.7	3.30 (m)
7	33.5	1.29 (m)/1.62 (m)	6′	62.8	3.69 (dd, 12.0, 5.4)<br>3.85 (dd, 12.0, 1.8)
8	40.6	-			
9	49.0	1.61*			
10	37.7	-			
11	24.6	1.89-1.92 (m)			
12	123.4	5.25 (br s)			
13	146.0	-			
14	43.0	-			
15	30.7	1.29 (m)/1.37 (m)			
16	23.7	2.00 (m)/1.60 (m)			
17	48.2	-			
18	43.0	2.88 (dd, 13.8, 4.2)			
19	47.5	1.70 (m)/1.13 (m)			
20	31.6	-			
21	35.1	1.22 (m)/1.40 (m)			
22	33.6	1.76 (m)/1.55 (m)			
23	65.0	3.65 (d, 12.0)/3.30 (d, 12.0)			
24	13.4	0.73 (s)			
25	16.1	1.00 (s)			
26	17.9	0.84 (s)			
27	26.5	1.19 (s)			
28	181.9	-			
29	33.6	0.92 (s)			
30	24.0	0.96 (s)			

[a)]150 MHz, [b)]600 MHz, [*)]Overlapped signal

Table S3. ^{1}H and ^{13}C NMR spectroscopic data for compound **5** in CD_3OD.

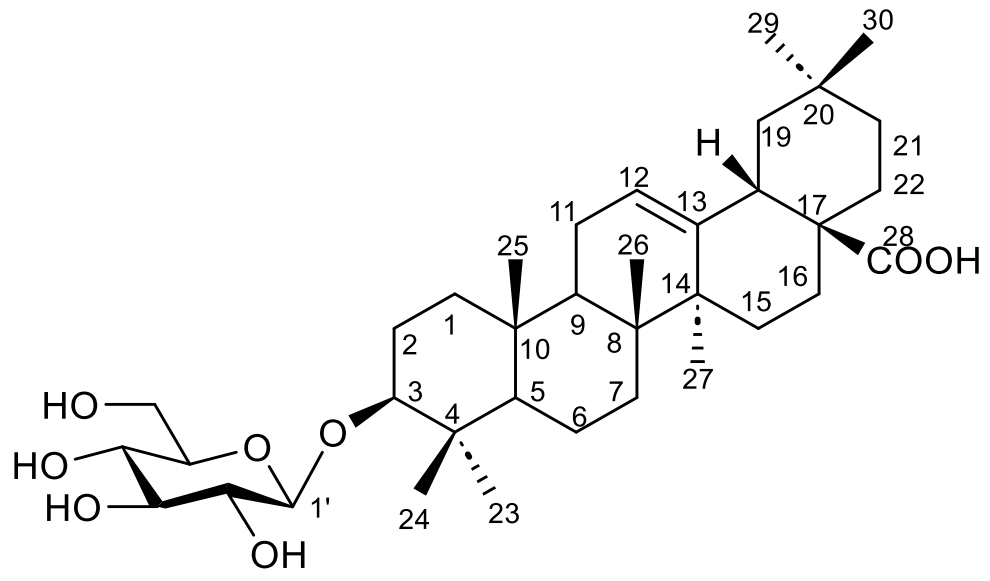

vitalboside A

Pos.	$^{a}\delta_C$	$^{b}\delta_H$ (mult., J in Hz)	Pos.	$^{a}\delta_C$	$^{b}\delta_H$ (mult., J in Hz)
1	39.8	1.02 (m)/1.63 (m)	3-O-glucose		
2	27.0	1.71 (m)/1.95 (m)	1′	106.7	4.34 (d, 7.8)
3	90.8	3.20 (dd, 11.4, 2.4)	2′	75.7	3.21 (dd, 9.0, 7.8)
4	40.2	-	3′	78.3	3.36 (t, 9.0)
5	57.1	0.82 (br d, 11.4)	4′	71.7	3.32 (t, 9.0)
6	19.3	1.42 (m)/1.58 (m)	5′	77.7	3.27 (ddd, 9.0, 5.4, 1.8)
7	34.0	1.34 (m)/1.55 (m)	6′	62.8	3.69 (dd, 12.0, 5.4)<br>3.86 (dd, 12.0, 1.8)
8	40.6	-			
9	49.0	1.61*			
10	37.9	-			
11	24.6	1.89-1.92 (m)			
12	123.7	5.29 (t, 3.6)			
13	145.2	-			
14	42.9	-			
15	28.9	1.10 (m)/1.80 (m)			
16	24.1	2.43 (td, 14.4, 3.6)/1.60 (m)			
17	47.7	-			
18	42.7	2.87 (dd, 13.8, 4.2)			
19	47.3	1.72 (t, 13.8)/1.14 (dd, 13.8, 4.2)			
20	31.6	-			
21	34.9	1.22 (m)/1.40 (m)			
22	33.8	1.77 (m)/1.55 (m)			
23	28.6	1.08 (s)			
24	17.0	0.87 (s)			
25	16.0	1.00 (s)			
26	17.8	0.84 (s)			
27	26.4	1.18 (s)			
28	181.9	-			
29	33.6	0.93 (s)			
30	24.0	0.98 (s)			

[a)]150 MHz, [b)]600 MHz, [*)]Overlapped signal

Table S4. ^{1}H and ^{13}C NMR spectroscopic data for compound **6** in CD_3OD.

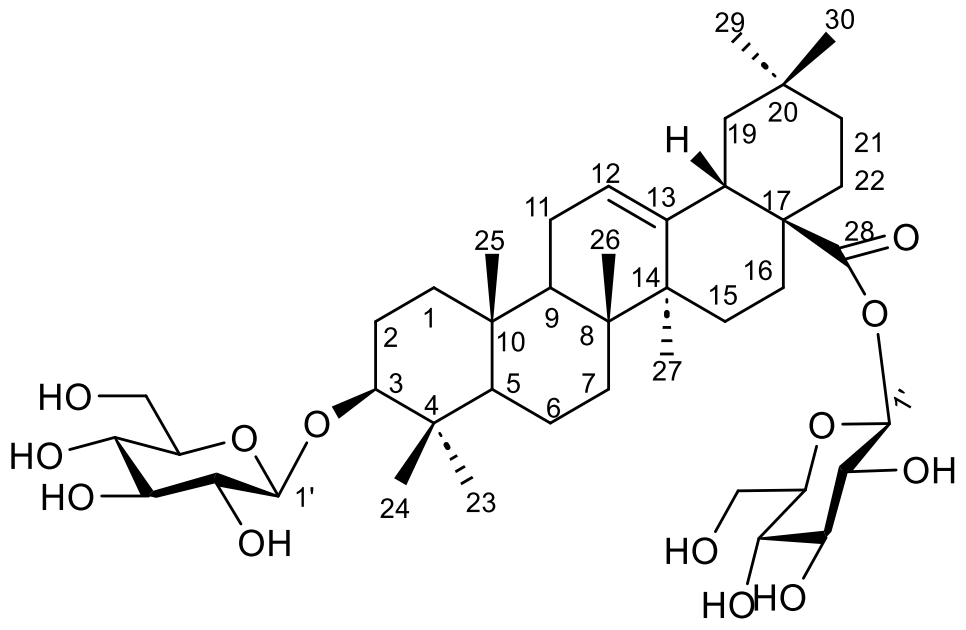

Lucyoside H

Pos.	$^a\delta_C$	$^b\delta_H$ (mult., J in Hz)	Pos.	$^a\delta_C$	$^b\delta_H$ (mult., J in Hz)
1	39.8	1.00 (m)/1.63 (m)	3-O-glucose		
2	27.0	1.71 (m)/1.95 (m)	1′	106.7	4.33 (d, 7.2)
3	90.8	3.20 (dd, 11.4, 2.4)	2′	75.7	3.21 (dd, 9.0, 7.2)
4	40.2	-	3′	78.3	3.36 (t, 9.0)
5	57.1	0.80 (br d, 11.4)	4′	71.7	3.30 (t, 9.0)
6	19.3	1.42 (m)/1.55 (m)	5′	77.7	3.26 (m)
7	34.0	1.33 (m)/1.48 (m)	6′	62.8	3.69 (dd, 12.0, 5.4)<br>3.82 (dd, 12.0, 1.8)
8	40.7	-	28-O-glucose		
9	49.0	1.60*	1″	95.7	5.40 (d, 8.4)
10	37.9	-	2″	74.2	3.33 (*)
11	24.6	1.91 (m)	3″	78.7	3.42 (t, 9.0)
12	123.8	5.27 (t, 3.6)	4″	71.2	3.37 (t, 9.0)
13	144.9	-	5″	78.9	3.34 (m)
14	42.9	-	6″	62.5	3.69 (dd, 12.0, 5.4)<br>3.82 (dd, 12.0, 1.8)
15	28.9	1.10 (m)/1.82 (m)			
16	24.0	2.07 (td, 14.4, 3.6)/1.73 (m)			
17	48.0	-			
18	42.6	2.88 (dd, 13.8, 4.2)			
19	47.2	1.72 (m)/1.17 (m)			
20	31.5	-			
21	34.9	1.22 (m)/1.42 (m)			
22	33.2	1.75 (m)/1.62 (m)			
23	28.5	1.07 (s)			
24	17.0	0.87 (s)			
25	16.0	0.98 (s)			
26	17.7	0.82 (s)			
27	26.3	1.17 (s)			
28	178.1	-			
29	33.5	0.92 (s)			
30	24.1	0.95 (s)			

a)150 MHz, b)600 MHz, *)Overlapped signal

Table S5. ^{1}H and ^{13}C NMR spectroscopic data for compound **7** in CD_3OD

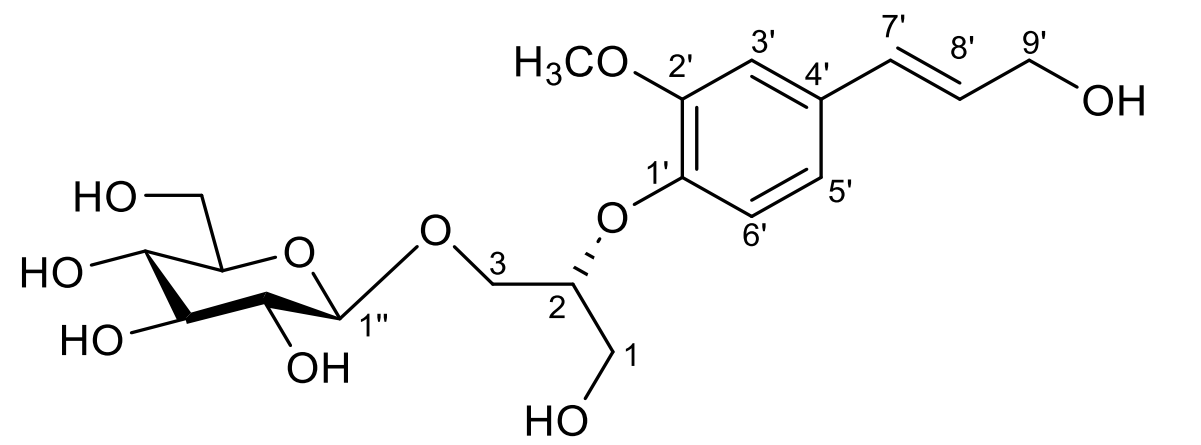

3-Hydroxy-2-[4-[(1E)-3-hydroxy-1-propen-1-yl]-2-methoxyphenoxy]propyl β-D-glucopyranoside

C	$^{a}\delta_C$	$^{b}\delta_H$ (mult., *J* in Hz)0
1	62.1	3.29-3.88*
2	81.0	4.44 (m)
3	68.9	3.86*/4.08 (dd, 10.8, 3.0)
1′	148.2	-
2′	151.5	-
3′	111.4	7.08*
4′	133.4	-
5′	120.7	6.97 (br d, 6.97)
6′	118.7	7.08*
7′	131.4	6.57 (d, 16.2)
8′	128.7	6.29 (dt, 16.2, 5.4)
9′	63.7	4.23 (d, 5.4)
2′-OCH_3	56.4	3.88 (s)
7-O-Glc		
1′	104.8	4.35 (d, 7.8)
2′	75.4	3.23 (dd, 9.0, 7.8)
3′	78.0	3.36*
4′	71.6	3.30*
5′	78.0	3.30*
6′	62.7	3.68 (dd, 12.0, 5.4)
		3.85*

[a)]150 MHz, [b)]600 MHz, [*)]Overlapped signal

Table S6. ^{1}H and ^{13}C NMR spectroscopic data for compound **8** in CD_3OD

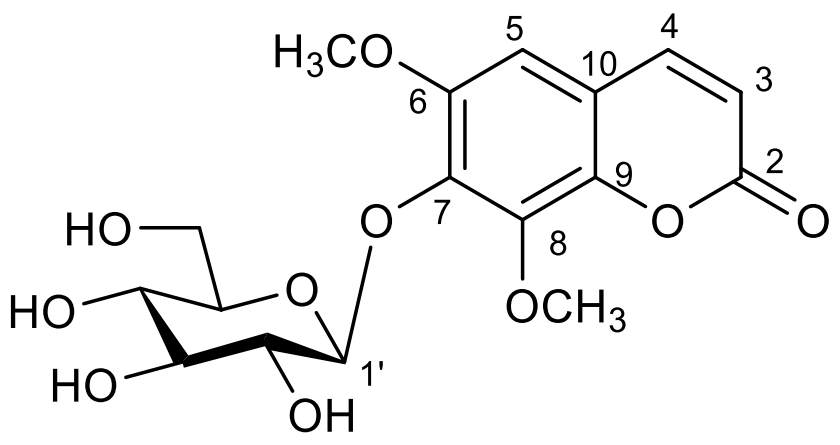

Isofraxidin 7-O-β-D-glucopyranoside

C	$^{a}\delta_C$	$^{b}\delta_H$ (mult., *J* in Hz)
2	162.6	-
3	115.8	6.39 (d, 9.6)
4	145.9	7.92 (d, 9.6)
5	106.2	7.04 (s)
6	151.5	-
7	143.6	-
8	142.4	-
9	144.1	-
10	116.7	-
6-OCH_3	57.2	3.93 (s)
8-OCH_3	62.6	4.05 (s)
7-O-Glc		
1′	104.2	5.22 (d, 7.8)
2′	75.7	3.54 (đ, 9.0, 7.8)
3′	78.0	3.46 (t, 9.0)
4′	71.4	3.42 (dd, 9.6, 9.0)
5′	78.6	3.26 (ddd, 9.6, 5.4, 2.4)
6′	62.5	3.66 (dd, 12.0, 5.4)
		3.80 (dd, 12.0, 2.4)

[a)]150 MHz, [b)]600 MHz

Table S7. ^{1}H and ^{13}C NMR spectroscopic data for compound **9** in CD_3OD

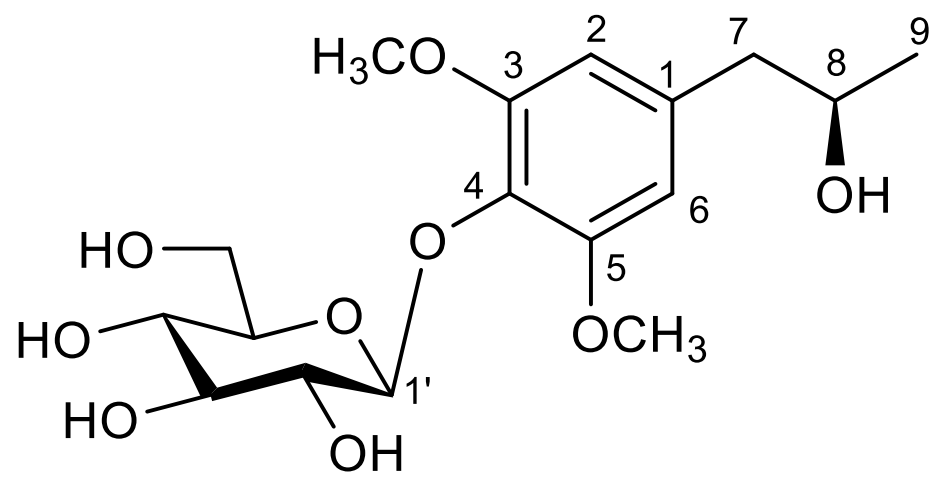

Pipercroside A

C	$^{a}\delta_C$	$^{b}\delta_H$ (mult., J in Hz)
1	137.4	-
2	108.5	6.59 (s)
3	154.0	-
4	134.7	-
5	154.0	-
6	108.5	-
7	46.8	2.56 (dd, 13.8, 5.4)
		2.72 (dd, 13.8, 6.6)
8	69.7	3.96-3.99 (m)
9	23.1	1.89 (d, 6.0)
3-OCH_3	57.0	3.87 (s)
5-OCH_3	57.0	3.87 (s)
4-O-Glc		
1′	105.7	4.80*
2′	75.8	3.48 (dd, 9.0, 7.8)
3′	77.9	3.43*
4′	71.4	3.43*
5′	78.3	3.22 (m)
6′	62.6	3.69 (dd, 12.0, 5.4)
		3.80 (dd, 12.0, 1.8)

a)150 MHz, b)600 MHz, *)Overlapped signal

Table S8. ^{1}H and ^{13}C NMR spectroscopic data for compound **10** in CD_3OD

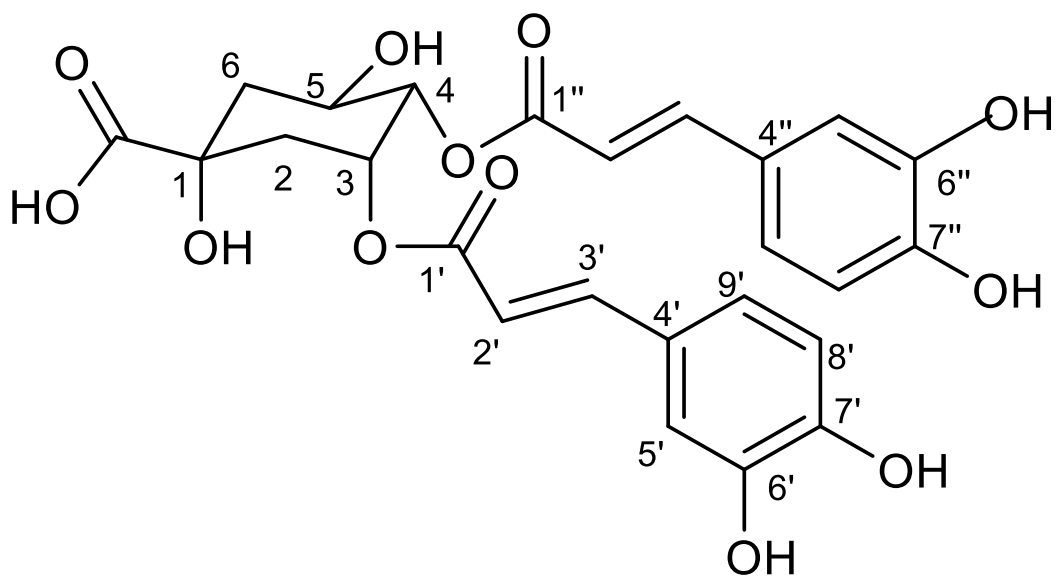

3,4-Di-O-caffeoylquinic acid

C	a,b δ_C	a,c δ_H (mult., *J* in Hz)
1	77.6	-
2	41.0	2.29 (dd, 9.5, 2.5)/2.20*
3	69.7	5.71 (m)
4	77.4	5.13 (dd, 10.0, 2.5)
5	70.9	4.80*
6	39.0	2.20*/2.03 (m)
7	180.6	-
1′	168.8	-
2′	116.5	6.28 (d, 15.5)
3′	147.4	7.60 (d, 15.5)
4′	127.6	-
5′	114.9	6.75 (d, 2.0)
6′	146.8	-
7′	149.8	-
8′	116.5	7.07 (d, 8.0)
9′	123.1	6.92 (dd, 8.0, 2.0)
1″	168.7	-
2″	116.5	6.21 (d, 15.5)
3″	147.6	7.53 (d, 15.5)
4″	127.7	-
5″	114.9	6.76 (d, 2.0)
6″	146.8	-
7″	149.8	-
8″	116.5	7.07 (d, 8.0)
9″	123.1	6.90 (dd, 8.0, 2.0)

a)125 MHz, b)500 MHz, *Overlapped signal

Table S9. ^{1}H and ^{13}C NMR spectroscopic data for compound **11** in CD_3OD

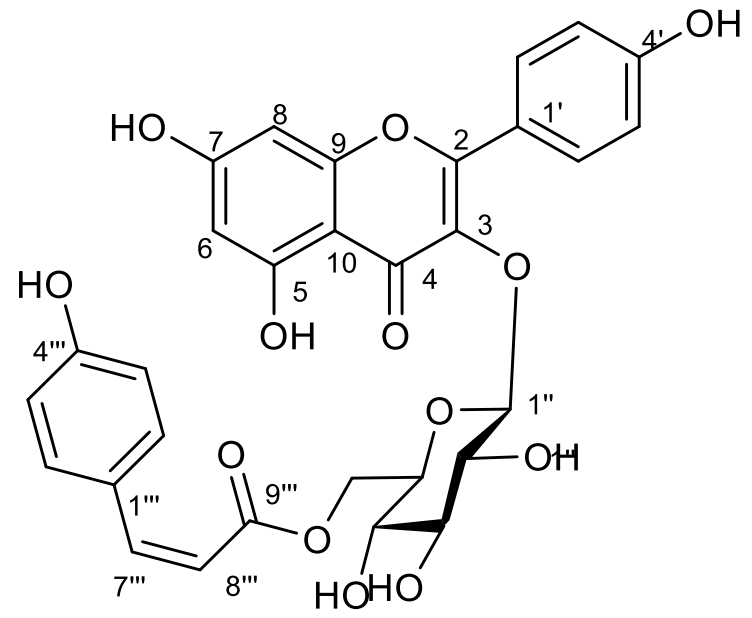

***Cis*-tiliroside**

C	[a,b]δ_C	[a,c]δ_H (mult., *J* in Hz)
2	159.3	-
3	135.2	-
4	179.3	-
5	163.0	-
6	100.2	6.20 (d, 1.8)
7	167.8	-
8	94.9	6.32 (d, 1.8)
9	158.6	-
10	104.1	-
1′	122.8	-
2′, 6′	132.2	8.00 (d, 8.4)
3′, 5′	115.7	6.84 (d, 8.4)
4′	161.5	-
3-O-Glucose		
1″	104.1	5.20 (d, 7.2)
2″	75.6	3.29 (dd, 9.0, 7.2)
3″	78.0	3.47 (t, 9.0)
4″	71.7	3.46 (t, 9.0)
5″	75.8	3.42 (m)
6″	64.1	4.17 (dd, 12.0, 6.0)
		4.23 (dd, 12.0, 2.4)
6″-O-Cumaroyl		
1‴	127.6	-
2‴, 6‴	135.2	7.53 (d, 8.4)
3‴, 5‴	116.0	6.70 (d, 8.4)
4‴	159.9	-
7‴	145.3	6.72 (d, 13.2)
8‴	114.8	6.09 (d, 13.2)
9‴	167.8	-

[a)]150 MHz, [b)]600 MHz
